# Predicting new-onset post-stroke depression from real-world data using machine learning algorithm

**DOI:** 10.3389/fpsyt.2023.1195586

**Published:** 2023-06-19

**Authors:** Yu-Ming Chen, Po-Cheng Chen, Wei-Che Lin, Kuo-Chuan Hung, Yang-Chieh Brian Chen, Chi-Fa Hung, Liang-Jen Wang, Ching-Nung Wu, Chih-Wei Hsu, Hung-Yu Kao

**Affiliations:** ^1^Department of Psychiatry, Kaohsiung Chang Gung Memorial Hospital, Chang Gung University College of Medicine, Kaohsiung, Taiwan; ^2^Department of Physical Medicine and Rehabilitation, Kaohsiung Chang Gung Memorial Hospital, Chang Gung University College of Medicine, Kaohsiung, Taiwan; ^3^Department of Diagnostic Radiology, Kaohsiung Chang Gung Memorial Hospital, Chang Gung University College of Medicine, Kaohsiung, Taiwan; ^4^Department of Anesthesiology, Chi Mei Medical Center, Tainan City, Taiwan; ^5^Department of Hospital and Health Care Administration, College of Recreation and Health Management, Chia Nan University of Pharmacy and Science, Tainan City, Taiwan; ^6^School of Medicine, College of Medicine, National Sun Yat-sen University, Kaohsiung, Taiwan; ^7^College of Humanities and Social Sciences, National Pingtung University of Science and Technology, Pingtung, Taiwan; ^8^Department of Child and Adolescent Psychiatry, Kaohsiung Chang Gung Memorial Hospital, Chang Gung University College of Medicine, Kaohsiung, Taiwan; ^9^Department of Otolaryngology, Kaohsiung Chang Gung Memorial Hospital, Chang Gung University College of Medicine, Kaohsiung, Taiwan; ^10^Department of Public Health, College of Medicine, National Cheng Kung University, Tainan City, Taiwan; ^11^Department of Computer Science and Information Engineering, National Cheng Kung University, Tainan City, Taiwan

**Keywords:** artificial intelligence, depressive disorder, electronic medical record, feature importance, prediction

## Abstract

**Introduction:**

Post-stroke depression (PSD) is a serious mental disorder after ischemic stroke. Early detection is important for clinical practice. This research aims to develop machine learning models to predict new-onset PSD using real-world data.

**Methods:**

We collected data for ischemic stroke patients from multiple medical institutions in Taiwan between 2001 and 2019. We developed models from 61,460 patients and used 15,366 independent patients to test the models’ performance by evaluating their specificities and sensitivities. The predicted targets were whether PSD occurred at 30, 90, 180, and 365 days post-stroke. We ranked the important clinical features in these models.

**Results:**

In the study’s database sample, 1.3% of patients were diagnosed with PSD. The average specificity and sensitivity of these four models were 0.83–0.91 and 0.30–0.48, respectively. Ten features were listed as important features related to PSD at different time points, namely old age, high height, low weight post-stroke, higher diastolic blood pressure after stroke, no pre-stroke hypertension but post-stroke hypertension (new-onset hypertension), post-stroke sleep-wake disorders, post-stroke anxiety disorders, post-stroke hemiplegia, and lower blood urea nitrogen during stroke.

**Discussion:**

Machine learning models can provide as potential predictive tools for PSD and important factors are identified to alert clinicians for early detection of depression in high-risk stroke patients.

## 1. Introduction

Ischemic stroke, which accounts for 87% of all strokes, is a severe neurological condition that results from the disturbance of blood supply to the brain, arising due to embolism or thrombosis (1). A total of 13.7 million people suffered from strokes in 2016, making it the second major cause of death and disability worldwide (2). Complications after ischemic stroke are common, and affective symptoms such as depression, mania, and other mental disturbances (3), may be a group of common symptoms that are underestimated (4). Among them, post-stroke depression (PSD) is a very severe mental disorder following a stroke that emerges early and contributes to the prolonged declined quality of life of a patient (5, 6). Therefore, early detection and diagnosis of PSD may be an important step in the timely treatment of stroke patients and the improvement of patients’ prognoses.

Clinicians have traditionally often used screening tests to identify PSD at an early stage. A prior study evaluated the Montgomery and Asberg Depression Rating Scale (MADRS) and Hospital Anxiety and Depression Scale (HADS) of stroke patients, and the tools demonstrated moderate performance (MADRS: sensitivity 70%, HADS: sensitivity 32%) (7). Another study compared the performance of four depression screening tests in post-stroke patients, and the result showed that the Whooley questions had the highest sensitivity (89%), followed by the Center for Epidemiologic Studies Depression Scale (80%), the Patient Health Questionnaire with 2-item (79%), and the Patient Health Questionnaire with 9-item (32%) (8). A prospective multicenter observational study reported a reliable scale to detect PSD with moderate sensitivity (65%) and specificity (74%) (9). Despite adequate performance demonstrated by the depression screening tools, they may be too time-consuming when being used by clinicians for PSD screening in clinical practice.

Machine learning models present as a possibly more efficient way to identify PSD. It is a novel method of processing and analyzing data that has been applied in many areas of psychiatry, such as predicting treatment outcomes in depression (10), managing treatment-resistant depression (11), differentiating between clinical anxiety and depression disorders (12), and the prediction of postpartum depression (13). eXtreme Gradient Boosting (XGBoost) is a machine learning algorithm with the technique to process big data efficiently and to assemble several weak classifiers to form a strong classifier (14). Furthermore, XGBoost can also generate the ranking for importance of the predictor features (15).

This study aimed to develop a machine learning-trained model to predict PSD. We accessed a Taiwanese multicenter electronic medical record database and selected the XGBoost algorithm to train the predictive model. We also ranked the importance of features in these machine learning models to further explain the models.

## 2. Materials and methods

### 2.1. Data collection and study subjects

**FIGURE 1:**
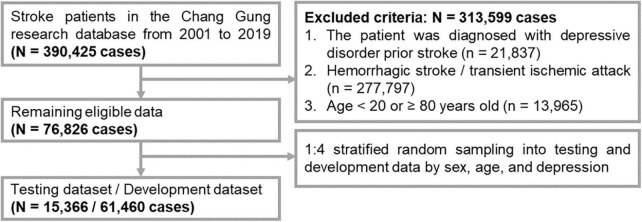
Flowchart of patient selection for this research.

The study protocol was approved by the Institutional Review Board of Chang Gung Memorial Hospital (No. 202002296B0). The flowchart for the selection of the subjects is shown in Figure 1. We collected the patients’ data from the Chang Gung Research Database (CGRD) from 1 January 2001 to 31 December 2020. The CGRD is a multicenter electronic medical record database for seven medical institutes in Taiwan, and contains de-identified personal data on medical visits (inpatient and outpatient), background information, diseases [diagnosed by the International Classification of Diseases (ICDs), such as ICD-9/ICD-10], medication records (type and dosage), and laboratory examinations (hematology tests and biochemistry tests). The CGRD database covered 14% of patients with mental illness Taiwan’s total population from 1997 to 2010 (16). We included patient records according to the following criteria: (1) first-time stroke (ICD-9: 430–438; ICD-10: I6, G45, and G46); and (2) observation period of at least 1 year after stroke. We excluded records with the following: (1) a diagnosis of depressive disorder prior to stroke; (2) hemorrhagic stroke (ICD-9: 430–432; ICD-10: I60–I62) or transient ischemic attack (ICD9: 435; ICD-10: I6784, G450, G451, G452, G458, G459, G460, G461, and G462); and (3) age <20 or ≥80 years.

To acquire a prediction model with good generalizability, the data were divided into a dataset for external examination (testing), and a dataset for internal development (training and validation). First, we performed 1:4 stratified random sampling according to age and sex to obtain an external dataset (for testing). Second, the remaining data was used as the developmental dataset (for training and validation) to develop the prediction model by the machine learning method and for data validation (17). Finally, we included 76,826 subjects for data processing.

### 2.2. Definition of study outcomes and model features

We defined PSD, our primary outcome, as at least one diagnosis of depressive disorders (ICD-9: 296.2, 296.3, 296.9, 300.4, and 311; ICD-10: F32, F33, F34.8, F34.9, and F39) following an ischemic stroke during either outpatient or inpatient care. The CGRD used ICD-9 codes for diagnoses from 2001 to 2015 and ICD-10 codes from 2016 to 2020 in this study. We retrieved the data at different time points to detect whether depression occurred within 1 month (0–30 days), one season (0–90 days), half a year (0–180 days), or 1 year (0–365 days).

To survey for candidate features to predict PSD, we extracted different features from inpatient and outpatient services for analysis, including demographic data (sex and age) during stroke, basic clinical information (height, weight, and blood pressure) during and after stroke, actively/poorly controlled comorbid mental disorders or medical diseases before and after stroke, concomitant medications after stroke, and laboratory data during and after stroke. Previous studies have shown that some patients’ data, including white blood cell counts and high blood pressure, are associated with PSD (18, 19). Therefore, we collected the above data at different time points (before, during, and after), which we defined as 1 year before stroke (before), 29 days before stroke to 1 day after stroke (during), and 29 days before time cutoff (or depression onset) to 1 day after (after). Because there may be multiple records at different time points during the study period (time-dependent variables), such as basic clinical information or laboratory data, if multiple records exist within the same time period, we used the average of these values as a single feature in our model. Detailed information on all features are provided in Supplementary Table 1.

### 2.3. Machine learning model and interpretation

We used XGBoost to predict the binary outcome (PSD or no PSD). XGBoost applies the decision tree by repetitively centering on harder to predict subunits of the training data (15). We used the XGBoost algorithm with 100 trees in a depth of six layers, and performed fivefold cross-validation to complete the XGBoost prediction model. Finally, we evaluated model performance using the testing dataset and reported the different parameters for each model, including specificity, sensitivity, and the area under the curve for receiver operator characteristic (AUC-ROC).

We used Shapley additive explanation (SHAP) to present the interpretability of the XGBoost model. SHAP was developed to give each feature an importance value for the prediction of the database. Each SHAP value of a particular feature indicated the contribution of the feature to the outcomes. In this study, a higher absolute value of SHAP indicates greater importance of the feature (top feature) in the predictive model. A positive SHAP value of a feature demonstrated an increased risk of depression for the patient and vice versa. The SHAP value of the variables are additive, which means we can convert the contribution of each variable into a part of the output grouping probability (20). Then, we re-ranked the top 10 ensemble features selected from the feature importance ranking results of the four machine learning models to investigate common important features. The method of finding ensemble features was used in our previous work (14). We performed the statistical analyses with the SAS software (SAS Institute Inc., Cary, NC, USA). The statistical significance was identified at *p*-value <0.05. The machine learning models were processed with Windows Python 3.8 (scikit-learn package v. 1.0.2).

## 3. Results

A total of 61,460 and 15,366 patients were divided into the development and test datasets, respectively. Approximately 1.3% of patients had PSD (development dataset: 775; test dataset: 194). In both datasets, the mean age was 63 years, 40% were female, the mean systolic/diastolic blood pressure were 135/77 mmHg, and the mean height/weight were 161–162 cm/63–64 kg. Among stroke patients, 6% had sleep-wake disorder, 2% had anxiety disorders, 39% had hypertension, and 3% had hemiplegia, all of which were actively or poorly controlled after stroke. The characteristics of the patient are presented in Table 1.

**TABLE 1:** Characteristics of subjects included in the development and test datasets.

Character	Development (*n* = 61,460)	Test (*n* = 15,366)
**Basic information (during)**		
Age	63.4 (11.7)	63.4 (11.6)
Sex, female	24,644 (40.1)	6,161 (40.1)
**Clinical characteristics (after)**		
SBP (mmHg)	135 (16)	135 (16)
DBP (mmHg)	77 (9)	77 (9)
Height (cm)	161.5 (6.9)	161.3 (6.8)
Weight (kg)	63.3 (9.0)	63.6 (8.8)
**Comorbidity (after)**		
Sleep-wake disorders	3,477 (5.7)	837 (5.5)
Anxiety disorders	1,336 (2.2)	306 (2.0)
Hypertension	23,890 (38.9)	5,916 (38.5)
Hemiplegia	1,836 (3.0)	439 (2.9)
**Laboratory data (during)**		
Blood urea nitrogen (mg/dl)	17.8 (12.7)	17.8 (12.7)
Serum creatinine (mg/dl)	1.2 (1.2)	1.2 (1.2)

Data are expressed as *N* (%) or mean (SD). DBP, diastolic blood pressure; SBP, systolic blood pressure.

Table 2 shows the model performance of XGBoost for predicting PSD at different time points. The overall prediction models had specificity between 0.83 and 0.91 and sensitivity between 0.30 and 0.48. The 30-day prediction model had the highest specificity (0.91) but the lowest sensitivity (0.30). The 365-day prediction model predicted PSD over time with the highest sensitivity (0.48) but the lowest specificity (0.83). Furthermore, the AUC-ROC of the four prediction models ranged from 0.64 to 0.71.

**TABLE 2:** Model performance of the XGBoost algorithm in predicting post stroke depression disorder.

	30 days	90 days	180 days	365 days
Specificity	0.91 (0.90–0.91)	0.88 (0.88–0.88)	0.86 (0.85–0.86)	0.83 (0.82–0.83)
Sensitivity	0.30 (0.28–0.32)	0.36 (0.35–0.38)	0.38 (0.37–0.40)	0.48 (0.47–0.48)
Accuracy	0.90 (0.89–0.90)	0.87 (0.87–0.87)	0.85 (0.84–0.85)	0.81 (0.81–0.82)
AUC-ROC	0.64 (0.63–0.64)	0.67 (0.67–0.68)	0.68 (0.67–0.69)	0.71 (0.71–0.72)

AUC-ROC, area under the curve of receiver operator characteristic; XGBoost, extreme gradient boosting.

Table 3 and Supplementary Figures 1–4 show the top 10 features in the four prediction models obtained by the XGBoost algorithm. For the ensemble features from all four models, old age, high height, low weight after stroke, higher diastolic blood pressure after stroke, new onset hypertension (no pre-stroke hypertension, but post-stroke hypertension), post-stroke sleep-wake disorders, post-stroke anxiety disorders, post-stroke hemiplegia, and lower blood urea nitrogen during stroke were associated with the occurrence of PSD. Among them, sleep-wake disorders after stroke ranked first in all four prediction models. All features used in the four models are detailed in Supplementary Table 2.

**TABLE 3:** Top 10 features predicting post-stroke depression at different time points.

	Ensemble	30 days	90 days	180 days	365 days
Top 1	Sleep-wake disorders (after)	Sleep-wake disorders (after)	Sleep-wake disorders (after)	Sleep-wake disorders (after)	Sleep-wake disorders (after)
Top 2	Hypertension (after)	Blood urea nitrogen (during)	Blood urea nitrogen (during)	Weight (after)	Weight (after)
Top 3	DBP (after)	Hypertension (after)	DBP (after)	SBP (after)	DBP (after)
Top 4	Age (during)	Hypertension (before)	Hypertension (after)	Hypertension (after)	SBP (after)
Top 5	Hypertension (before)	Bleed (before)	DBP (during)	DBP (after)	Hypertension (after)
Top 6	Hemiplegia (after)	White blood cell (during)	Weight (after)	Hemiplegia (after)	Hemiplegia (after)
Top 7	Blood urea nitrogen (during)	Mean corpuscular hemoglobin concentration (during)	Hypertension (before)	Anxiety disorders (after)	Age (during)
Top 8	Weight (after)	Age (during)	White blood cell (during)	White blood cell-lymphocyte (during)	Red blood cell (during)
Top 9	Anxiety disorders (after)	DBP (after)	Hemiplegia (after)	Weight (during)	DBP (during)
Top 10	Height (during)	White blood cell-lymphocyte (during)	Mean corpuscular hemoglobin concentration (during)	Age (during)	Anxiety disorders (after)

DBP, diastolic blood pressure; SBP: systolic blood pressure.

## 4. Discussion

This study developed 30, 90, 180, and 365-day PSD prediction models with the XGBoost algorithm using real data from inpatient and outpatient electronic medical records. In these four models, specificity, sensitivity, accuracy, and AUC-ROC were 83–91, 30–48, 81–90, and 64–71%, respectively. Moreover, we found that the top 10 features in these predictive models included: old age, high height, low weight after stroke, new-onset hypertension (especially higher diastolic blood pressure), post-stroke sleep-wake disorders, post-stroke hemiplegia, post-stroke anxiety disorders, and lower blood urea nitrogen.

Only 1.3% of the patients developed new-onset PSD in our dataset. The prevalence is lower than previous results. A meta-analysis reported the prevalence of depression was 18% in post-stroke patients (21). The discrepancy in prevalence may be attributed to two possible reasons. First, we excluded all patients with a history of depressive disorder prior to stroke (Figure 1, *n* = 21,837), which may further reduce the incidence of new-onset PSD in this study, as previous depression is an important risk factor for PSD (22). Second, cultural stoicism, as noted in prior epidemiological research in Taiwan, may contribute to a lower prevalence of major depressive disorder in the Taiwanese population (1.2%) compared to their counterparts in Western countries (23). Regarding the performance of our models compared to previous research, a prospective observational study using the Melancholy index of the Hamilton Depression Rating Scale (HDRS) ≥1.5 as a predictor found an association with PSD at 3-month follow-up with a specificity of 90% and a sensitivity of 53% (24). In comparison, the predictive models in this study showed comparability (specificity 83–91%, sensitivity 30–48%). The relatively low sensitivity observed in our models may be attributed to differences in features compared to those found in depression assessment scales such as HDRS. Our model does not include emotion-related features like depressive mood, loss of interest, or suicidal ideation, which are typically present in these scales. Instead, our model focuses more on somatic features, such as sleep disorders and body weight, and incorporates patient background factors, such as age and hypertension. Nonetheless, our model offers greater clinical feasibility advantages in real-world practice. As our predictive model only requires access to existing medical records, eliminating the need for a new time-consuming interview, it presents an opportunity for integration into hospital systems in the future. By utilizing the background information of stroke patients, our model can provide PSD predictions. This could act as an alert for non-psychiatric healthcare professionals, facilitating early referrals to psychiatric specialists for prompt intervention and management. Another issue is the optimal time points for follow-up of PSD. Our study found that the AUC-ROC of the four models increased over time after stroke, and the 365-day cutoff had the best predictive performance, with an AUC-ROC of 71%. Current machine learning algorithms appear to be better at predicting PSD at long-term follow-up (1 year) compared to predicting depression in the acute phase after stroke (1 month). These findings are similar to those of previous studies. One prospective study showed that significant predictors of PSD were found at 12-month follow-up but not at 3-month follow-up (25). Another study found that aphasia 6 months after stroke and related problems 18 months after stroke were associated with depression (26).

Post-stroke sleep-wake disorders was the most influential feature for the prediction of PSD. A meta-analysis reported a 38% prevalence of post-stroke insomnia (27). Numerous studies have found an association between sleep and depression (28). One retrospective study indicated that total sleep time shorter than 6 h could predict PSD (29), and another randomized controlled trial found that interventions to improve sleep quality was able to reduce symptoms of depression (30). The underlying relationships between sleep-wake disorders and depression may have some biochemical causes, such as serotonin and proinflammatory cytokines. First, brain lesions can disrupt ascending projections from the midbrain and brainstem to the frontal cortex, reducing serotonin bioavailability. This neurotransmitter, when released into the diencephalon and cerebrum, may inhibit sleep promotion. The raphe nuclei contain 80% of all brain serotonin neurons, and serotonin was initially believed to be a key neuromodulator of sleep and mood, as its depletion in the raphe system led to insomnia and depression (31). Second, sleep disturbances may elevate inflammatory cytokines like interleukin-6 and tumor necrosis factor (32). This inflammation could, in turn, raise the likelihood of developing depression (33). This potential connection helps explain why sleep-wake disorders are crucial in predicting PSD. Furthermore, post-stroke anxiety was also a relevant feature for predicting PSD in this study. Anxiety symptoms after stroke are common, and a meta-analysis showed a 29% pooled prevalence of post-stroke anxiety disorder (34). One study showed a significant association between anxiety and depression in the post-stroke period (35), while another study found a significant association between post-stroke anxiety and sleep disturbance (reduced daytime and nighttime sleep time) (36).

Post-stroke hypertension and higher diastolic blood pressure on post-stroke physical examination were associated with PSD in our predictive models. One prior study demonstrated that hypertension plays a role in predicting 3-month PSD (37). Another study reported that a longer duration of hypertension was also associated with new-onset depression after stroke (38). Another survey examined multiple vascular risk factors (hypertension, diabetes, hyperlipidemia, smoking, and obesity), and found that only hypertension was an independent predictor of PSD (18). The vascular depression hypothesis postulates a role for vascular lesions in PSD (39). Hypertension demonstrates a classic vascular risk factor and is associated with white matter hyperintensities, which may be a possible pathophysiology of depression in later life (40). Additionally, our model found no association between pre-stroke hypertension and PSD. Combined with the above findings, new-onset hypertension (no pre-stroke hypertension, but post-stroke hypertension) and uncontrolled hypertension after stroke may have greater impact on PSD.

Older age was a predictor of PSD in our model, which supports previous studies. In patients with lacunar stroke/small vessel diseases, elderly patients are more likely to develop depression than younger patients (41), and frontal periventricular age-related white matter hyperintensity is associated with early-onset PSD (42). Moreover, our results also showed that low weight after stroke was associated with PSD. This may be due to poor appetite, a symptom of depressive disorder, leading to lower body weight. As for the association of higher height with PSD, it might be more informative to consider it in conjunction with weight. At the same weight, higher height might represent a lower body mass index, which could indicate malnutrition. Poor nutritional status could be a consequence of depression (due to decreased appetite) (43). Hemiplegia was also an influential feature. Hemiplegia is a severe neurological deficit that negatively affects the patient’s daily life. A prospective study demonstrated that stroke patients with hemiplegia had lower quality of life and more depressive symptoms (44). In respect of functional outcomes of stroke survivors, one research indicated that hemiparesis was associated with self-reported general health and subjective feeling of depression (45). A prospective study using the Barthel index reported that severe functional impairment was a predictor of PSD at 12-month follow-up (25). In this study, the top 10 features predicting PSD were different at different time points. For example, hemiplegia revealed a valid feature (top 10) for predicting depression in the 90, 180, and 365-day models, with increasing ranking over time, but not among the top 10 features in the 30-day model (Table 3). Functional impairment due to hemiplegia may worsen depressive symptoms. A longitudinal study noted that severe depression was associated with higher levels of functional impairment 6 months after stroke compared with 48 h after stroke (46).

This study has some advantages. First, the model was developed from a real-world electronic medical record database and it represents the characteristics of local patients. The medical staff can use this clinical tool to predict PSD conveniently without complex evaluation and facilitate prompt subsequent treatment of depression to improve patients’ quality of life. There are several limitations in the interpretation of the data of this study. First, the database included all stroke patients with depression-related diagnoses, and the severity of depression was not analyzed in the prediction model, which may reduce the test validity. Second, individuals with a history of prior traumatic events or psychosocial factors were not analyzed in this study. Third, the effect of ongoing/no treatment of depression after stroke was not considered in the data analysis. Fourth, information regarding stroke severity and the location of brain lesions was not available in CGRD. Epilepsy, multiple sclerosis, dementia, or other neurological problems that could exacerbate depressive symptoms after stroke were not excluded during model development. These factors may also influence the occurrence of PSD. Fifth, the impact of education level was not tested in the prediction model. Sixth, the patient population in this study consisted of individuals aged between 20 and 79 years; therefore, we might not be able to generalize the predictive model to younger or older individuals. Seventh, depressive disorders identified in the CGRD were evaluated by different clinicians and used two different diagnostic systems (ICD-9 and ICD-10) across different periods of time (from 2001 to 2019) (47, 48). Under these circumstances, CGRD has not yet demonstrated the validity or reliability of these depression diagnoses. However, a *post hoc* analysis indicated that all depression patients in our study were diagnosed by psychiatrists at least once, and psychiatrists in Taiwan are well-trained by the Taiwanese Society of Psychiatry to ensure standard and consistent coding behaviors. Accordingly, we believe that the diagnosis of depressive disorder we defined should be relatively sound.

This study collected real-world electronic medical records from multicenter medical centers and developed PSD prediction models for different time periods. Overall specificity, sensitivity, accuracy, and AUC-ROC were 83–91, 30–48, 81–90, and 64–71%, respectively. The models revealed the top 10 important features, such as post-stroke sleep-wake disorder, uncontrolled blood pressure after stroke, and old age. The model handles complex real-world clinical records and provides a potential utility for predicting PSD.

## Data availability statement

The original contributions presented in this study are included in the article/Supplementary material, further inquiries can be directed to the corresponding author.

## Ethics statement

The studies involving human participants were reviewed and approved by the Institutional Review Board of Chang Gung Memorial Hospital (No. 202002296B0). Written informed consent for participation was not required for this study in accordance with the national legislation and the institutional requirements.

## Author contributions

C-WH conceived the research idea for the study, contributed to data acquisition and extraction, and performed the statistical analysis. C-WH led the study design, with Y-MC, P-CC, and H-YK. Y-MC verified the underlying data and drafted the manuscript first. Y-MC, P-CC, W-CL, K-CH, Y-CC, C-FH, L-JW, C-NW, C-WH, and H-YK revised the manuscript. All authors contributed important intellectual content during manuscript revision, had full access to all the data in the study, and accepted responsibility to submit for publication.
