# Supplementary material for: Predicting new-onset post-stroke depression from real-world data using machine learning algorithm
Source: Front Psychiatry. 2023 Jun 19;14:1195586. doi: 10.3389/fpsyt.2023.1195586 (PMC10315461; doi:10.3389/fpsyt.2023.1195586)
Supplement: Supplementary file 1 [file Data_Sheet_1.docx]

**Supplementary information**

**Predicting new-onset post-stroke depression from real-world data using machine learning algorithm**

**Running Title**: post-stroke depression prediction

Yu-Ming Chen^1,†^, Po-Cheng Chen^2,†^, Wei-Che Lin^3^, Kuo-Chuan Hung^4,5^, Yang-Chieh Brian Chen^1^, Chi-Fa Hung^1,6,7^, Liang-Jen Wang^8^, Ching-Nung Wu^9,10^, Chih-Wei Hsu^1,11,*^, Hung-Yu Kao^11^

^1^ Department of Psychiatry, Kaohsiung Chang Gung Memorial Hospital and Chang Gung University College of Medicine, Kaohsiung, Taiwan

^2^ Department of Physical Medicine and Rehabilitation, Kaohsiung Chang Gung Memorial Hospital, College of Medicine, Chang Gung University, Kaohsiung, Taiwan

^3^ Department of Diagnostic Radiology, Kaohsiung Chang Gung Memorial Hospital, Chang Gung University College of Medicine, Kaohsiung, Taiwan

^4^ Department of Anesthesiology, Chi Mei Medical Center, Tainan city, Taiwan

^5^ Department of Hospital and Health Care Administration, College of Recreation and Health Management, Chia Nan University of Pharmacy and Science, Tainan city, Taiwan

^6^ School of Medicine, College of Medicine, National Sun Yat-Sen University, Kaohsiung, Taiwan

^7^ College of Humanities and Social Sciences, National Pintung University of Science and Technology, Pingtung, Taiwan

^8^ Department of Child and Adolescent Psychiatry, Kaohsiung Chang Gung Memorial Hospital and Chang Gung University College of Medicine, Kaohsiung, Taiwan

^9^ Department of Otolaryngology, Kaohsiung Chang Gung Memorial Hospital and Chang Gung University College of Medicine, Kaohsiung, Taiwan

^10^ Department of Public Health, College of Medicine, National Cheng Kung University, Tainan, Taiwan

^11^ Department of Computer Science and Information Engineering, National Cheng Kung University, Tainan, Taiwan

^†^ Equal contribution and first authorship

* Corresponding author

| **Content** | **Page** |
| --- | --- |
| **Supplementary Table 1**. Detailed diagnostic codes for mental disorders or medical diseases and drug name with codes | 3-26 |
| **Supplementary Table 2.** All feature importance ranking results of the 4 different machine learning algorithms | 27-63 |
| **Supplementary Figures 1.** Shapley additive explanations method for selecting the top 10 features in extreme gradient boost, 30-day | 64 |
| **Supplementary Figures 2.** Shapley additive explanations method for selecting the top 10 features in extreme gradient boost, 90-day | 65 |
| **Supplementary Figures 3.** Shapley additive explanations method for selecting the top 10 features in extreme gradient boost, 180-day | 66 |
| **Supplementary Figures 4.** Shapley additive explanations method for selecting the top 10 features in extreme gradient boost, 365-day | 67 |

**Supplementary Table 1**. Detailed diagnostic codes for mental disorders or medical diseases and drug name with codes

| Mental disorders / Medical diseases | The International Classification of Diseases 9^th^ (ICD-9) or 10^th^ (ICD-10) |
| --- | --- |
| Mental disorders |  |
| Neurodevelopmental disorders | 299, 307.2, 307.3, 307.9, 314, 315, 317, 318, 319, F44.4, F63.3, F70, F71, F72, F73, F78, F79, F80.0, F80.1, F80.2, F80.4, F80.89, F80.9, F81, F82, F84.0, F84.3, F84.5, F84.8, F84.9, F88, F89, F90.1, F90.2, F90.8, F90.9, F95, F98.4, F98.8, H93.25, R41.840, R41.841, R41.843, R41.844, R45.1, R45.81, R45.82, R48.0 |
| Disruptive, impulse-control, and conduct disorders | 312.32, 312.33, 312.34, 312.8, 312.9, 313.81, F63.1, F63.2, F63.81, F9 |
| Schizophrenia spectrum and other psychotic disorders | 295, 297.0, 297.1, 297.2, 298.3, 298.4, 298.8, 298.9, F20, F22, F23, F25, F28, F2 |
| Bipolar and related disorders | 296.0, 296.1, 296.4, 296.5, 296.6, 296.7, 296.80, 296.89, 301.13, F30.1, F30.2, F30.3, F30.4, F30.9, F31, F34 |
| Depressive disorders | 296.2, 296.3, 296.99, 300.4, 311, 625.4, F32.0, F32.1, F32.2, F32.3, F32.4, F32.5, F32.9, F33, F34.1, F34.8, F34.9, G43.82, G43.83, N94 |
| Anxiety disorders | 300.00, 300.01, 300.02, 300.09, 300.2, 309.21, 313.23, F40, F41, F93.0, F94 |
| Obsessive-compulsive and related disorders | 300.3, 300.7, 312.39, 698.4, F42, F45.2, F63.3, F63.89, L98 |
| Somatic symptom and related disorders | 300.11, 300.19, 300.7, 300.8, 316, F44.2, F44.4, F44.5, F44.6, F44.7, F45.0, F45.1, F45.2, F45.8, F45.9, F48.8, F54, F68 |
| Elimination disorders | 307.6, 307.7, 787.60, 788.30, 788.39, F98.0, F98.1, N39.490, N39.498, R15, R32, R39.8 |
| Sleep-wake disorders | 307.4, 327, 347, 780.5, F51, G47.0, G47.1, G47.2, G47.3, G47.4, G47.5, G47.61, G47.69, G47.8, G47 |
| Sexual dysfunctions | 302.7, F52.0, F52.1, F52.2, F52.3, F52.4, F52.6, F52.8, F52.9, R3 |
| Substance-related and addictive disorders | 291.4, 291.81, 292.0, 292.2, 303, 304, 305, 312.31, F10.10, F10.120, F10.129, F10.20, F10.21, F10.220, F10.229, F10.230, F10.232, F10.239, F10.920, F10.929, F11.10, F11.120, F11.129, F11.20, F11.21, F11.220, F11.229, F11.23, F11.90, F11.920, F11.929, F11.93, F12.10, F12.120, F12.129, F12.20, F12.21, F12.220, F12.229, F12.90, F12.920, F12.929, F13.10, F13.120, F13.129, F13.20, F13.21, F13.220, F13.229, F13.23, F13.90, F13.920, F13.929, F13.93, F14.10, F14.120, F14.129, F14.20, F14.21, F14.220, F14.229, F14.23, F14.90, F14.920, F14.929, F15.10, F15.120, F15.129, F15.20, F15.21, F15.220, F15.229, F15.23, F15.90, F15.920, F15.929, F15.93, F16.10, F16.120, F16.129, F16.20, F16.21, F16.220, F16.229, F16.90, F16.920, F16.929, F17.200, F17.201, F17.203, F17.210, F17.211, F17.213, F17.220, F17.221, F17.223, F17.290, F17.291, F17.293, F18.10, F18.120, F18.129, F18.20, F18.21, F18.220, F18.229, F18.90, F18.920, F18.929, F19.10, F19.120, F19.129, F19.20, F19.21, F19.220, F19.229, F19.23, F19.90, F19.920, F19.929, F19.93, F55, F63 |
| Neurocognitive disorders (Except vascular dementia) | F01, F02, F05, G30, G31, G31.0, G31.1, G93.7, 290.1, 290.2, 290.3, 294, 331.0, 331.1, 331.2, 331.8, 331.9, F03.90, G31.83, G31.85, G31.89, F03.90, G31.83, G31.85, G31.8 |
| Personality disorders | 301.0, 301.20, 301.22, 301.4, 301.50, 301.6, 301.7, 301.81, 301.82, 301.83, 301.89, 301.9, F21, F60, F6 |
| Trauma- and stressor-related disorders | 308.3, 309.0, 309.24, 309.28, 309.3, 309.4, 309.81, 309.89, 309.9, 313.89, F43.0, F43.1, F43.21, F43.22, F43.23, F43.24, F43.25, F43.8, F43.9, F93.8, F94.1, F94.2, F94.9, F98 |
| Dissociative disorders | 300.12, 300.13, 300.14, 300.15, 300.6, F44.0, F44.1, F44.81, F44.9, F48 |
| Feeding and eating disorders | 307.1, 307.5, F50, F98.2, F98 |
| Gender dysphoria | 302.6, 302.85, F64.1, F64.2, F64.8, F64 |
| Paraphilic disorders | 302.2, 302.3, 302.4, 302.81, 302.82, 302.83, 302.84, 302.89, 302.9, F65, F6 |
| Medical diseases |  |
| Myocardial infarct | 410, 412, I21, I22, I25 |
| Congestive heart failure | 398.91, 402.01, 402.11, 402.91, 404.01, 404.03, 404.11, 404.13, 404.91, 404.93, 425.4, 425.5, 425.6, 425.7, 425.8, 425.9, 428, I5 |
| Peripheral vascular diseases | 047.1, 093.0, 437.3, 440, 441, 443.1, 443.2, 443.3, 443.4, 443.5, 443.6, 443.7, 443.8, 443.9, 557.1, 557.9, I71, I73.9, I79.0, R02, Z95.8, Z95 |
| Cerebrovascular diseases | 362.34, 430, 431, 432, 433, 434, 435, 436, 437, 438, G45.0, G45.1, G45.2, G45.4, G45.8, G45.9, G46, I60, I61, I62, I63, I64, I65, I66, I67.0, I67.1, I67.2, I67.4, I67.5, I67.6, I67.7, I67.8, I67.9, I68.1, I68.2, I68.8, I6 |
| Hemiplegia | 334.1, 342, 344.0, 344.1, 344.2, 344.3, 344.4, 344.5, 344.6, 344.9, G04.1, G81, G82.0, G82.1, G82 |
| Chronic pulmonary diseases | 416.8, 416.9, 490, 491, 492, 493, 494, 495, 496, 497, 498, 499, 500, 501, 502, 503, 504, 505, 506.4, 508.1, 508.8, J40, J41, J42, J43, J44, J44, J45, J46, J47, J60, J61, J62, J63, J64, J65, J66, J6 |
| Peptic ulcer diseases | 531, 532, 533, 534, K25, K26, K27, K28 |
| Liver diseases without sequelae | 070.22, 070.23, 070.32, 070.33, 070.44, 070.54, 070.6, 070.9, 570, 571, 573.3, 573.4, 573.8, K70.2, K70.3, K71.7, K73, K74.0, K74.2, K74.3, K74.4, K74.5, K74.6, V42 |
| Liver diseases with sequelae | 456.0, 456.1, 456.2, 572.2, 572.3, 572.4, 572.5, 572.6, 572.7, 572.8, K72.1, K72.9, K76.6, K76 |
| Renal diseases | 403.01, 403.11, 403.91, 404.02, 404.03, 404.12, 404.13, 404.92, 404.93, 582, 583.0, 583.1, 583.2, 583.3, 583.4, 583.5, 583.6, 583.7, 585, 586, 588.0, N01, N03, N05.2, N05.3, N05.4, N05.5, N05.6, N07.2, N07.3, N07.4, N18, N19, N25, V42.0, V45.1, V5 |
| Connective tissue diseases | 446.5, 710.0, 710.1, 710.2, 710.3, 710.4, 725, M05.0, M05.1, M05.2, M05.3, M05.8, M05.9, M06.0, M06.3, M06.9, M32, M33.2, M34, M35 |
| Any cancer | 140, 141, 142, 143, 144, 145, 146, 147, 148, 149, 150, 151, 152, 153, 154, 155, 156, 157, 158, 159, 160, 161, 162, 163, 164, 165, 166, 167, 168, 169, 170, 171, 172, 174, 175, 176, 177, 178, 179, 180, 181, 182, 183, 184, 185, 186, 187, 188, 189, 190, 191, 192, 193, 194, 195, 195.0, 195.1, 195.2, 195.3, 195.4, 195.5, 195.6, 195.7, 195.8, 200, 201, 202, 203, 204, 205, 206, 207, 208, 238.6, C0, C1, C2, C3, C40, C41, C43, C45, C46, C47, C48, C49, C5, C6, C70, C71, C72, C73, C74, C75, C76, C80, C81, C82, C83, C84, C85, C88.3, C88.7, C88.9, C90.0, C90.1, C91, C92, C93, C94.0, C94.1, C94.2, C94.3, C94.51, C94.7, C95, C9 |
| Metastatic solid tumor | 196, 197, 198, 199, C77, C78, C79, C8 |
| Acquired immune deficiency syndrome | 042, 043, 044, B20, B21, B22, B23, B2 |
| Hypertension | 401, 402, 403, 404, 405, I10, I11, I12, I13, I15, N26 |
| Hyperlipidemia | 272, E75.2, E75.3, E75.5, E75.6, E77, E78, E88.1, E88.2, E88.8 |
| Diabetes mellitus without end organ damage | 250.0, 250.1, 250.2, 250.3, 250.8, 250.9, E10.1, E10.5, E10.9, E11.1, E11.5, E11.9, E13.1, E13.5, E13.9, E14.1, E14.5, E14 |
| Diabetes mellitus with end organ damage | 250.4, 250.5, 250.6, 250.7, E10.2, E10.3, E10.4, E11.2, E11.3, E11.4, E13.2, E13.3, E13.4, E14.2, E14.3, E14 |
| Intracranial bleeding | 430, 431, 432.0, 432.1, 432.9, 852.0, 852.2, 852.4, 853.0, I60, I61, I62, S06.340A, S06.341A, S06.342A, S06.343A, S06.344A, S06.345A, S06.346A, S06.347A, S06.348A, S06.349A, S06.350A, S06.351A, S06.352A, S06.353A, S06.354A, S06.355A, S06.356A, S06.357A, S06.358A, S06.359A, S06.360A, S06.361A, S06.362A, S06.363A, S06.364A, S06.365A, S06.366A, S06.367A, S06.368A, S06.369A, S06.4x0A, S06.4x1A, S06.4x2A, S06.4x3A, S06.4x4A, S06.4x5A, S06.4x6A, S06.4x7A, S06.4x8A, S06.4x9A, S06.5x0A, S06.5x1A, S06.5x2A, S06.5x3A, S06.5x4A, S06.5x5A, S06.5x6A, S06.5x7A, S06.5x8A, S06.5x9A, S06.6x0A, S06.6x1A, S06.6x2A, S06.6x3A, S06.6x4A, S06.6x5A, S06.6x6A, S06.6x7A, S06.6x8A, S06.6x9 |
| Ocular bleeding | 360.43, 362.81, 363.6, 364.4, 372.72, 376.32, 377.42, 379.23, H05.23, H11.3, H21.0, H31.3, H35.6, H43.1, H44.8, H47.0 |
| Intraabdominal bleeding | 568.81, 866.01, 866.02, 866.11, 866.12, K66.1, S31.001A, S37.011 |
| Hemopericardium or pericardial bleeding | 423.0, I31 |
| Hematoma or compartment syndrome | 599.7, M79.A, N30.01, N30.21, N30.31, N30.81, N30.91, R3 |
| Gastrointestinal bleeding | 530.7, 531, 531.2, 531.4, 531.6, 532, 533, 534, 535.01, 535.11, 535.21, 535.31, 535.41, 535.51, 535.61, 535.71, 537.83, 537.84, 562.02, 562.03, 562.12, 562.13, 569.3, 569.85, 578, K22.6, K25, K26, K27, K28, K29.01, K29.21, K29.31, K29.41, K29.51, K29.61, K29.71, K29.81, K29.91, K31.811, K31.82, K52.81, K55.21, K56.60, K56.60, K57.01, K57.11, K57.13, K57.21, K57.31, K57.33, K57.81, K57.91, K57.93, K62.5, K92.0, K92.1, K92 |
| Other bleeding | 302.7, F52.0, F52.1, F52.2, F52.3, F52.4, F52.6, F52.8, F52.9, R3 |
|  |  |
| Chlorhexidine | A01AB03 |
| Triamcinolone | A01AC01 |
| Dexamethasone | A01AC02 |
| Hydrocortisone | A01AC03 |
| A01AC91 | A01AC91 |
| Epinephrine | A01AD01 |
| Magnesium oxide | A02AA02 |
| Aluminium compounds | A02AB |
| Aluminium hydroxide | A02AB01 |
| Ordinary salt combinations | A02AD01 |
| Hydrotalcite | A02AD04 |
| Ordinary salt combinations and antiflatulents | A02AF02 |
| Antacids with antispasmodics | A02AG |
| Antacids with sodium bicarbonate | A02AH |
| Antacids, other combinations | A02AX |
| Cimetidine | A02BA01 |
| Ranitidine | A02BA02 |
| Famotidine | A02BA03 |
| Misoprostol | A02BB01 |
| Omeprazole | A02BC01 |
| Pantoprazole | A02BC02 |
| Lansoprazole | A02BC03 |
| Rabeprazole | A02BC04 |
| Esomeprazole | A02BC05 |
| Dexlansoprazole | A02BC06 |
| Sucralfate | A02BX02 |
| Alginic acid | A02BX13 |
| Mebeverine | A03AA04 |
| Glycopyrronium bromide | A03AB02 |
| Propantheline | A03AB05 |
| Otilonium bromide | A03AB06 |
| Mepenzolate | A03AB12 |
| Papaverine | A03AD01 |
| Pinaverium | A03AX04 |
| Alverine | A03AX08 |
| A03AX95 | A03AX95 |
| Atropine | A03BA01 |
| Butylscopolamine | A03BB01 |
| Metoclopramide | A03FA01 |
| Cisapride | A03FA02 |
| Domperidone | A03FA03 |
| Mosapride | A03FA09 |
| Ondansetron | A04AA01 |
| Granisetron | A04AA02 |
| Tropisetron | A04AA03 |
| Palonosetron | A04AA05 |
| Palonosetron, combinations | A04AA55 |
| Aprepitant | A04AD12 |
| Chenodeoxycholic acid | A05AA01 |
| Ursodeoxycholic acid | A05AA02 |
| Other drugs for bile therapy | A05AX |
| Liver therapy | A05BA |
| Silymarin | A05BA03 |
| Bisacodyl | A06AB02 |
| Senna glycosides | A06AB06 |
| Contact laxatives in combination | A06AB20 |
| Ispaghula (psylla seeds) | A06AC01 |
| Polycarbophil calcium | A06AC08 |
| Sterculia, combinations | A06AC53 |
| Mineral salts in combination | A06AD10 |
| Lactulose | A06AD11 |
| Lactitol | A06AD12 |
| Macrogol | A06AD15 |
| Sodium phosphate | A06AD17 |
| Nystatin | A07AA02 |
| Vancomycin | A07AA09 |
| Fidaxomicin | A07AA12 |
| Medicinal charcoal | A07BA01 |
| Diosmectite | A07BC05 |
| Combinations | A07BC30 |
| Oral rehydration salt formulations | A07CA |
| Loperamide | A07DA03 |
| Budesonide | A07EA06 |
| Sulfasalazine | A07EC01 |
| Mesalazine | A07EC02 |
| Lactic acid producing organisms, combinations | A07FA51 |
| Racecadotril | A07XA04 |
| Multienzymes (lipase, protease etc.) | A09AA02 |
| Tilactase | A09AA04 |
| Multienzymes and acid preparations | A09AC02 |
| Insulin (human) | A10AB01 |
| Insulin lispro | A10AB04 |
| Insulin aspart | A10AB05 |
| Insulin glulisine | A10AB06 |
| Insulin (human) | A10AC01 |
| Insulin (human) | A10AD01 |
| Insulin lispro | A10AD04 |
| Insulin aspart | A10AD05 |
| Insulin (human) | A10AE01 |
| Insulin glargine | A10AE04 |
| Insulin detemir | A10AE05 |
| Insulin degludec | A10AE06 |
| Insulin glargine and lixisenatide | A10AE54 |
| Metformin | A10BA02 |
| Chlorpropamide | A10BB02 |
| Glipizide | A10BB07 |
| Gliclazide | A10BB09 |
| Glimepiride | A10BB12 |
| Metformin and sulfonylureas | A10BD02 |
| Metformin and rosiglitazone | A10BD03 |
| Metformin and pioglitazone | A10BD05 |
| Metformin and sitagliptin | A10BD07 |
| Metformin and vildagliptin | A10BD08 |
| Pioglitazone and alogliptin | A10BD09 |
| Metformin and saxagliptin | A10BD10 |
| Metformin and linagliptin | A10BD11 |
| Metformin and dapagliflozin | A10BD15 |
| Linagliptin and empagliflozin | A10BD19 |
| Metformin and empagliflozin | A10BD20 |
| Saxagliptin and dapagliflozin | A10BD21 |
| Acarbose | A10BF01 |
| Rosiglitazone | A10BG02 |
| Pioglitazone | A10BG03 |
| Sitagliptin | A10BH01 |
| Vildagliptin | A10BH02 |
| Saxagliptin | A10BH03 |
| Alogliptin | A10BH04 |
| Linagliptin | A10BH05 |
| Exenatide | A10BJ01 |
| Liraglutide | A10BJ02 |
| Dulaglutide | A10BJ05 |
| Dapagliflozin | A10BK01 |
| Canagliflozin | A10BK02 |
| Empagliflozin | A10BK03 |
| Guar gum | A10BX01 |
| Repaglinide | A10BX02 |
| Nateglinide | A10BX03 |
| Mitiglinide | A10BX08 |
| Multivitamins and iron | A11AA01 |
| Multivitamins and other minerals, incl. Combinations | A11AA03 |
| Calcitriol | A11CC04 |
| Thiamine (vit B1) | A11DA01 |
| Vitamin B1 in combination with vitamin B6 and/or vitamin B12 | A11DB |
| Vitamin B-complex, other combinations | A11EX |
| Ascorbic acid (vit C) | A11GA01 |
| Pyridoxine (vit B6) | A11HA02 |
| Tocopherol (vit E) | A11HA03 |
| Pyridoxal phosphate | A11HA06 |
| Calcium gluconate | A12AA03 |
| Calcium carbonate | A12AA04 |
| Calcium (different salts in combination) | A12AA20 |
| A12AA91 | A12AA91 |
| Calcium, combinations with vitamin D and/or other drugs | A12AX |
| Potassium chloride | A12BA01 |
| Potassium gluconate | A12BA05 |
| Potassium chloride, combinations | A12BA51 |
| Zinc gluconate | A12CB02 |
| Fluoride, combinations | A12CD51 |
| Nandrolone | A14AB01 |
| Levocarnitine | A16AA01 |
| Imiglucerase | A16AB02 |
| Agalsidase alfa | A16AB03 |
| Agalsidase beta | A16AB04 |
| Sodium phenylbutyrate | A16AX03 |
| Zinc acetate | A16AX05 |
| Miglustat | A16AX06 |
| Trientine | A16AX12 |
| A16AX95 | A16AX95 |
| A16AX96 | A16AX96 |
| Warfarin | B01AA03 |
| Heparin | B01AB01 |
| Enoxaparin | B01AB05 |
| Nadroparin | B01AB06 |
| Tinzaparin | B01AB10 |
| Clopidogrel | B01AC04 |
| Ticlopidine | B01AC05 |
| Acetylsalicylic acid | B01AC06 |
| Dipyridamole | B01AC07 |
| Iloprost | B01AC11 |
| Tirofiban | B01AC17 |
| Treprostinil | B01AC21 |
| Prasugrel | B01AC22 |
| Cilostazol | B01AC23 |
| Ticagrelor | B01AC24 |
| Selexipag | B01AC27 |
| Combinations | B01AC30 |
| Streptokinase | B01AD01 |
| Alteplase | B01AD02 |
| Urokinase | B01AD04 |
| Drotrecogin alfa (activated) | B01AD10 |
| Dabigatran etexilate | B01AE07 |
| Rivaroxaban | B01AF01 |
| Apixaban | B01AF02 |
| Edoxaban | B01AF03 |
| Fondaparinux | B01AX05 |
| Tranexamic acid | B02AA02 |
| Aprotinin | B02AB01 |
| B02AB91 | B02AB91 |
| Phytomenadione | B02BA01 |
| Blood coagulation factors | B02BD |
| Coagulation factor VIII | B02BD02 |
| Factor VIII inhibitor bypassing activity | B02BD03 |
| Coagulation factor IX | B02BD04 |
| Coagulation factor viia | B02BD08 |
| Romiplostim | B02BX04 |
| Eltrombopag | B02BX05 |
| Saccharated iron oxide | B03AB02 |
| Ferric hydroxide | B03AB04 |
| Iron, parenteral preparations | B03AC |
| Iron, multivitamins and folic acid | B03AE02 |
| Iron and multivitamins | B03AE03 |
| Various combinations | B03AE10 |
| Hydroxocobalamin | B03BA03 |
| Cobamamide | B03BA04 |
| Mecobalamin | B03BA05 |
| Folic acid | B03BB01 |
| Erythropoietin | B03XA01 |
| Darbepoetin alfa | B03XA02 |
| Methoxy polyethylene glycol-epoetin beta | B03XA03 |
| Albumin | B05AA01 |
| Dextran | B05AA05 |
| Gelatin agents | B05AA06 |
| Hydroxyethylstarch | B05AA07 |
| Amino acids | B05BA01 |
| Fat emulsions | B05BA02 |
| Carbohydrates | B05BA03 |
| Combinations | B05BA10 |
| Electrolytes | B05BB01 |
| Electrolytes with carbohydrates | B05BB02 |
| Solutions producing osmotic diuresis | B05BC |
| Mannitol | B05BC01 |
| B05BC92 | B05BC92 |
| Sodium chloride | B05CB01 |
| Combinations | B05CB10 |
| Hypertonic solutions | B05DB |
| Potassium chloride | B05XA01 |
| Sodium bicarbonate | B05XA02 |
| Sodium chloride | B05XA03 |
| Magnesium sulfate | B05XA05 |
| Potassium phosphate, incl. Combinations with other potassium salts | B05XA06 |
| Calcium chloride | B05XA07 |
| Electrolytes in combination with other drugs | B05XA31 |
| Vitamins | B05XC |
| Hemodialytics, concentrates | B05ZA |
| Hemin | B06AB01 |
| Digoxin | C01AA05 |
| Quinidine | C01BA01 |
| Procainamide | C01BA02 |
| Disopyramide | C01BA03 |
| Lidocaine | C01BB01 |
| Mexiletine | C01BB02 |
| Propafenone | C01BC03 |
| Flecainide | C01BC04 |
| Amiodarone | C01BD01 |
| Dronedarone | C01BD07 |
| Etilefrine | C01CA01 |
| Isoprenaline | C01CA02 |
| Norepinephrine | C01CA03 |
| Dopamine | C01CA04 |
| Phenylephrine | C01CA06 |
| Dobutamine | C01CA07 |
| Midodrine | C01CA17 |
| Epinephrine | C01CA24 |
| Milrinone | C01CE02 |
| Glyceryl trinitrate | C01DA02 |
| Isosorbide dinitrate | C01DA08 |
| Isosorbide mononitrate | C01DA14 |
| Nicorandil | C01DX16 |
| Alprostadil | C01EA01 |
| Ubidecarenone | C01EB09 |
| Adenosine | C01EB10 |
| Ivabradine | C01EB17 |
| Methyldopa (racemic) | C02AB02 |
| Clonidine | C02AC01 |
| Prazosin | C02CA01 |
| Doxazosin | C02CA04 |
| Hydralazine | C02DB02 |
| Minoxidil | C02DC01 |
| Nitroprusside | C02DD01 |
| Bosentan | C02KX01 |
| Ambrisentan | C02KX02 |
| Macitentan | C02KX04 |
| Riociguat | C02KX05 |
| Thiazides, plain | C03AA |
| Indapamide | C03BA11 |
| Furosemide | C03CA01 |
| Bumetanide | C03CA02 |
| Spironolactone | C03DA01 |
| Eplerenone | C03DA04 |
| Hydrochlorothiazide and potassium-sparing agents | C03EA01 |
| Tolvaptan | C03XA01 |
| Phentolamine | C04AB01 |
| Nicotinic acid | C04AC01 |
| Xantinol nicotinate | C04AD02 |
| Pentoxifylline | C04AD03 |
| Ergoloid mesylates | C04AE01 |
| Nicergoline | C04AE02 |
| Phenoxybenzamine | C04AX02 |
| Hydrocortisone | C05AA01 |
| Betamethasone | C05AA05 |
| Local anesthetics | C05AD |
| Cinchocaine | C05AD04 |
| Organo-heparinoid | C05BA01 |
| Heparinoid, combinations | C05BA51 |
| C05CX93 | C05CX93 |
| Propranolol | C07AA05 |
| Sotalol | C07AA07 |
| Nadolol | C07AA12 |
| Metoprolol | C07AB02 |
| Atenolol | C07AB03 |
| Bisoprolol | C07AB07 |
| Esmolol | C07AB09 |
| Nebivolol | C07AB12 |
| Labetalol | C07AG01 |
| Carvedilol | C07AG02 |
| Amlodipine | C08CA01 |
| Felodipine | C08CA02 |
| Nicardipine | C08CA04 |
| Nifedipine | C08CA05 |
| Nimodipine | C08CA06 |
| Lercanidipine | C08CA13 |
| Verapamil | C08DA01 |
| Diltiazem | C08DB01 |
| Captopril | C09AA01 |
| Enalapril | C09AA02 |
| Ramipril | C09AA05 |
| Fosinopril | C09AA09 |
| ACE inhibitors and calcium channel blockers | C09BB |
| Losartan | C09CA01 |
| Valsartan | C09CA03 |
| Irbesartan | C09CA04 |
| Candesartan | C09CA06 |
| Telmisartan | C09CA07 |
| Olmesartan medoxomil | C09CA08 |
| Azilsartan medoxomil | C09CA09 |
| Losartan and diuretics | C09DA01 |
| Valsartan and diuretics | C09DA03 |
| Irbesartan and diuretics | C09DA04 |
| Candesartan and diuretics | C09DA06 |
| Telmisartan and diuretics | C09DA07 |
| Valsartan and amlodipine | C09DB01 |
| Olmesartan medoxomil and amlodipine | C09DB02 |
| Telmisartan and amlodipine | C09DB04 |
| Candesartan and amlodipine | C09DB07 |
| Valsartan, amlodipine and hydrochlorothiazide | C09DX01 |
| Olmesartan medoxomil, amlodipine and hydrochlorothiazide | C09DX03 |
| Valsartan and sacubitril | C09DX04 |
| Aliskiren | C09XA02 |
| Simvastatin | C10AA01 |
| Fluvastatin | C10AA04 |
| Atorvastatin | C10AA05 |
| Rosuvastatin | C10AA07 |
| Pitavastatin | C10AA08 |
| Gemfibrozil | C10AB04 |
| Fenofibrate | C10AB05 |
| Colestyramine | C10AC01 |
| Ezetimibe | C10AX09 |
| Evolocumab | C10AX13 |
| Alirocumab | C10AX14 |
| Lovastatin and nicotinic acid | C10BA01 |
| Simvastatin and ezetimibe | C10BA02 |
| Atorvastatin and amlodipine | C10BX03 |
| Clotrimazole | D01AC01 |
| Ketoconazole | D01AC08 |
| Sulconazole | D01AC09 |
| Oxiconazole | D01AC11 |
| Sertaconazole | D01AC14 |
| Ciclopirox | D01AE14 |
| Combinations | D01AE20 |
| Naftifine | D01AE22 |
| Butenafine | D01AE23 |
| Griseofulvin | D01BA01 |
| Terbinafine | D01BA02 |
| Zinc products | D02AB |
| Carbamide | D02AE01 |
| Cadexomer iodine | D03AX01 |
| Centella asiatica herba | D03AX14 |
| Diphenhydramine | D04AA32 |
| Lidocaine | D04AB01 |
| D04AX91 | D04AX91 |
| Tars | D05AA |
| Calcipotriol | D05AX02 |
| Calcitriol | D05AX03 |
| Tazarotene | D05AX05 |
| Calcipotriol, combinations | D05AX52 |
| Trioxysalen | D05BA01 |
| Methoxsalen | D05BA02 |
| Acitretin | D05BB02 |
| Fusidic acid | D06AX01 |
| Neomycin | D06AX04 |
| Gentamicin | D06AX07 |
| Mupirocin | D06AX09 |
| Silver sulfadiazine | D06BA01 |
| Silver sulfadiazine, combinations | D06BA51 |
| Tromantadine | D06BB02 |
| Aciclovir | D06BB03 |
| Podophyllotoxin | D06BB04 |
| Metronidazole | D06BX01 |
| Hydrocortisone | D07AA02 |
| Clobetasone | D07AB01 |
| Triamcinolone | D07AB09 |
| Betamethasone | D07AC01 |
| Fluocinolone acetonide | D07AC04 |
| Diflucortolone | D07AC06 |
| Fluocinonide | D07AC08 |
| Mometasone | D07AC13 |
| Fluticasone | D07AC17 |
| Clobetasol | D07AD01 |
| Hydrocortisone and antibiotics | D07CA01 |
| Betamethasone and antibiotics | D07CC01 |
| Fluocinolone acetonide and antibiotics | D07CC02 |
| Hydrocortisone | D07XA01 |
| Flumetasone | D07XB01 |
| Mometasone | D07XC03 |
| Nitrofural | D08AF01 |
| Povidone-iodine | D08AG02 |
| Cetrimide | D08AJ04 |
| Sulfur | D10AB02 |
| Tretinoin | D10AD01 |
| Adapalene | D10AD03 |
| Benzoyl peroxide | D10AE01 |
| Clindamycin | D10AF01 |
| Azelaic acid | D10AX03 |
| Isotretinoin | D10BA01 |
| Wart and anti-corn preparations | D11AF |
| Tacrolimus | D11AH01 |
| Dupilumab | D11AH05 |
| Antibiotics | G01AA |
| Nystatin | G01AA01 |
| Metronidazole | G01AF01 |
| Clotrimazole | G01AF02 |
| Fenticonazole | G01AF12 |
| Sertaconazole | G01AF19 |
| Policresulen | G01AX03 |
| Methylergometrine | G02AB01 |
| Ergometrine | G02AB03 |
| Dinoprostone | G02AD02 |
| Ritodrine | G02CA01 |
| Bromocriptine | G02CB01 |
| Cabergoline | G02CB03 |
| Levonorgestrel | G03AC03 |
| Methyltestosterone | G03BA02 |
| Testosterone | G03BA03 |
| Estradiol | G03CA03 |
| Estriol | G03CA04 |
| Conjugated estrogens | G03CA57 |
| Medroxyprogesterone | G03DA02 |
| Progesterone | G03DA04 |
| Dydrogesterone | G03DB01 |
| Dienogest | G03DB08 |
| Norethisterone | G03DC02 |
| Testosterone and estrogen | G03EA02 |
| Androgen, progestogen and estrogen in combination | G03EB |
| Norethisterone and estrogen | G03FA01 |
| Progesterone and estrogen | G03FA04 |
| Medroxyprogesterone and estrogen | G03FA12 |
| Chorionic gonadotrophin | G03GA01 |
| Clomifene | G03GB02 |
| Cyproterone | G03HA01 |
| Cyproterone and estrogen | G03HB01 |
| Danazol | G03XA01 |
| Gestrinone | G03XA02 |
| Raloxifene | G03XC01 |
| Bazedoxifene | G03XC02 |
| Urinary concrement solvents | G04BC |
| Flavoxate | G04BD02 |
| Oxybutynin | G04BD04 |
| Propiverine | G04BD06 |
| Tolterodine | G04BD07 |
| Solifenacin | G04BD08 |
| Mirabegron | G04BD12 |
| Alprostadil | G04BE01 |
| Sildenafil | G04BE03 |
| Phenazopyridine | G04BX06 |
| Pentosan polysulfate sodium | G04BX15 |
| Alfuzosin | G04CA01 |
| Tamsulosin | G04CA02 |
| Terazosin | G04CA03 |
| Silodosin | G04CA04 |
| Tamsulosin and dutasteride | G04CA52 |
| Finasteride | G04CB01 |
| Dutasteride | G04CB02 |
| Tetracosactide | H01AA02 |
| Thyrotropin alfa | H01AB01 |
| Somatropin | H01AC01 |
| Vasopressin (argipressin) | H01BA01 |
| Desmopressin | H01BA02 |
| Terlipressin | H01BA04 |
| Oxytocin | H01BB02 |
| Gonadorelin | H01CA01 |
| Somatostatin | H01CB01 |
| Octreotide | H01CB02 |
| Lanreotide | H01CB03 |
| Fludrocortisone | H02AA02 |
| Glucocorticoids | H02AB |
| Dexamethasone | H02AB02 |
| Methylprednisolone | H02AB04 |
| Prednisolone | H02AB06 |
| Triamcinolone | H02AB08 |
| Hydrocortisone | H02AB09 |
| Cortisone | H02AB10 |
| Levothyroxine sodium | H03AA01 |
| Propylthiouracil | H03BA02 |
| Carbimazole | H03BB01 |
| Thiamazole | H03BB02 |
| Glucagon | H04AA01 |
| Teriparatide | H05AA02 |
| Calcitonin (salmon synthetic) | H05BA01 |
| Doxycycline | J01AA02 |
| Oxytetracycline | J01AA06 |
| Tetracycline | J01AA07 |
| Minocycline | J01AA08 |
| Tigecycline | J01AA12 |
| Chloramphenicol | J01BA01 |
| Ampicillin | J01CA01 |
| Amoxicillin | J01CA04 |
| Piperacillin | J01CA12 |
| Benzylpenicillin | J01CE01 |
| Phenoxymethylpenicillin | J01CE02 |
| Benzathine benzylpenicillin | J01CE08 |
| Dicloxacillin | J01CF01 |
| Oxacillin | J01CF04 |
| Sulbactam | J01CG01 |
| Ampicillin and beta-lactamase inhibitor | J01CR01 |
| Amoxicillin and beta-lactamase inhibitor | J01CR02 |
| Piperacillin and beta-lactamase inhibitor | J01CR05 |
| Cefalexin | J01DB01 |
| Cefalotin | J01DB03 |
| Cefazolin | J01DB04 |
| Cefadroxil | J01DB05 |
| Cefradine | J01DB09 |
| Cefoxitin | J01DC01 |
| Cefuroxime | J01DC02 |
| Cefaclor | J01DC04 |
| Flomoxef | J01DC14 |
| Cefotaxime | J01DD01 |
| Ceftazidime | J01DD02 |
| Ceftriaxone | J01DD04 |
| Ceftizoxime | J01DD07 |
| Cefixime | J01DD08 |
| Ceftibuten | J01DD14 |
| Ceftazidime and beta-lactamase inhibitor | J01DD52 |
| Cefoperazone and beta-lactamase inhibitor | J01DD62 |
| Cefepime | J01DE01 |
| Cefpirome | J01DE02 |
| Aztreonam | J01DF01 |
| Meropenem | J01DH02 |
| Ertapenem | J01DH03 |
| Doripenem | J01DH04 |
| Imipenem and cilastatin | J01DH51 |
| Ceftaroline fosamil | J01DI02 |
| Sulfamethoxazole and trimethoprim | J01EE01 |
| Erythromycin | J01FA01 |
| Clarithromycin | J01FA09 |
| Azithromycin | J01FA10 |
| Clindamycin | J01FF01 |
| Streptomycin | J01GA01 |
| Gentamicin | J01GB03 |
| Kanamycin | J01GB04 |
| Neomycin | J01GB05 |
| Amikacin | J01GB06 |
| Netilmicin | J01GB07 |
| Isepamicin | J01GB11 |
| Ciprofloxacin | J01MA02 |
| Levofloxacin | J01MA12 |
| Moxifloxacin | J01MA14 |
| Gemifloxacin | J01MA15 |
| Nalidixic acid | J01MB02 |
| Vancomycin | J01XA01 |
| Teicoplanin | J01XA02 |
| Colistin | J01XB01 |
| Fusidic acid | J01XC01 |
| Metronidazole | J01XD01 |
| Nitrofurantoin | J01XE01 |
| Fosfomycin | J01XX01 |
| Linezolid | J01XX08 |
| Daptomycin | J01XX09 |
| Amphotericin B | J02AA01 |
| Ketoconazole | J02AB02 |
| Fluconazole | J02AC01 |
| Itraconazole | J02AC02 |
| Voriconazole | J02AC03 |
| Posaconazole | J02AC04 |
| Flucytosine | J02AX01 |
| Caspofungin | J02AX04 |
| Micafungin | J02AX05 |
| Anidulafungin | J02AX06 |
| Calcium aminosalicylate | J04AA03 |
| Cycloserine | J04AB01 |
| Rifampicin | J04AB02 |
| Rifamycin | J04AB03 |
| Rifabutin | J04AB04 |
| Isoniazid | J04AC01 |
| Pyrazinamide | J04AK01 |
| Ethambutol | J04AK02 |
| Rifampicin and isoniazid | J04AM02 |
| Rifampicin, pyrazinamide and isoniazid | J04AM05 |
| Rifampicin, pyrazinamide, ethambutol and isoniazid | J04AM06 |
| Rifampicin, ethambutol and isoniazid | J04AM07 |
| Aciclovir | J05AB01 |
| Ganciclovir | J05AB06 |
| Famciclovir | J05AB09 |
| Valaciclovir | J05AB11 |
| Valganciclovir | J05AB14 |
| Ritonavir | J05AE03 |
| Atazanavir | J05AE08 |
| Darunavir | J05AE10 |
| Zidovudine | J05AF01 |
| Didanosine | J05AF02 |
| Stavudine | J05AF04 |
| Lamivudine | J05AF05 |
| Abacavir | J05AF06 |
| Tenofovir disoproxil | J05AF07 |
| Adefovir dipivoxil | J05AF08 |
| Entecavir | J05AF10 |
| Telbivudine | J05AF11 |
| Tenofovir alafenamide | J05AF13 |
| Nevirapine | J05AG01 |
| Efavirenz | J05AG03 |
| Etravirine | J05AG04 |
| Rilpivirine | J05AG05 |
| Oseltamivir | J05AH02 |
| Raltegravir | J05AJ01 |
| Dolutegravir | J05AJ03 |
| Antivirals for treatment of HCV infections | J05AP |
| Ribavirin | J05AP01 |
| Asunaprevir | J05AP06 |
| Daclatasvir | J05AP07 |
| Sofosbuvir | J05AP08 |
| Dasabuvir | J05AP09 |
| Sofosbuvir and ledipasvir | J05AP51 |
| Ombitasvir, paritaprevir and ritonavir | J05AP53 |
| Elbasvir and grazoprevir | J05AP54 |
| Sofosbuvir and velpatasvir | J05AP55 |
| Glecaprevir and pibrentasvir | J05AP57 |
| Zidovudine and lamivudine | J05AR01 |
| Lamivudine and abacavir | J05AR02 |
| Tenofovir disoproxil and emtricitabine | J05AR03 |
| Emtricitabine, tenofovir disoproxil and efavirenz | J05AR06 |
| Emtricitabine, tenofovir disoproxil and rilpivirine | J05AR08 |
| Lopinavir and ritonavir | J05AR10 |
| Lamivudine, abacavir and dolutegravir | J05AR13 |
| Darunavir and cobicistat | J05AR14 |
| Emtricitabine, tenofovir alafenamide, elvitegravir and cobicistat | J05AR18 |
| Emtricitabine, tenofovir alafenamide and rilpivirine | J05AR19 |
| Emtricitabine, tenofovir alafenamide and bictegravir | J05AR20 |
| Dolutegravir and rilpivirine | J05AR21 |
| Emtricitabine, tenofovir alafenamide, darunavir and cobicistat | J05AR22 |
| Letermovir | J05AX18 |
| Tetanus antitoxin | J06AA02 |
| Snake venom antiserum | J06AA03 |
| Immunoglobulins, normal human, for intravascular adm. | J06BA02 |
| J06BA03 | J06BA03 |
| Anti-D (rh) immunoglobulin | J06BB01 |
| Tetanus immunoglobulin | J06BB02 |
| Hepatitis B immunoglobulin | J06BB04 |
| Cytomegalovirus immunoglobulin | J06BB09 |
| Palivizumab | J06BD01 |
| Tetanus toxoid | J07AM01 |
| Rabies, inactivated, whole virus | J07BG01 |
| Cyclophosphamide | L01AA01 |
| Chlorambucil | L01AA02 |
| Melphalan | L01AA03 |
| Ifosfamide | L01AA06 |
| Bendamustine | L01AA09 |
| Busulfan | L01AB01 |
| Carmustine | L01AD01 |
| Temozolomide | L01AX03 |
| Dacarbazine | L01AX04 |
| Methotrexate | L01BA01 |
| Pemetrexed | L01BA04 |
| Pralatrexate | L01BA05 |
| Mercaptopurine | L01BB02 |
| Tioguanine | L01BB03 |
| Cladribine | L01BB04 |
| Fludarabine | L01BB05 |
| Cytarabine | L01BC01 |
| Fluorouracil | L01BC02 |
| Tegafur | L01BC03 |
| Gemcitabine | L01BC05 |
| Capecitabine | L01BC06 |
| Azacitidine | L01BC07 |
| Tegafur, combinations | L01BC53 |
| Trifluridine, combinations | L01BC59 |
| Vinblastine | L01CA01 |
| Vincristine | L01CA02 |
| Vinorelbine | L01CA04 |
| Etoposide | L01CB01 |
| Paclitaxel | L01CD01 |
| Docetaxel | L01CD02 |
| Topotecan | L01CE01 |
| Irinotecan | L01CE02 |
| Dactinomycin | L01DA01 |
| Doxorubicin | L01DB01 |
| Daunorubicin | L01DB02 |
| Epirubicin | L01DB03 |
| Idarubicin | L01DB06 |
| Mitoxantrone | L01DB07 |
| Bleomycin | L01DC01 |
| Mitomycin | L01DC03 |
| Imatinib | L01EA01 |
| Dasatinib | L01EA02 |
| Nilotinib | L01EA03 |
| Ponatinib | L01EA05 |
| Gefitinib | L01EB01 |
| Erlotinib | L01EB02 |
| Afatinib | L01EB03 |
| Osimertinib | L01EB04 |
| Vemurafenib | L01EC01 |
| Crizotinib | L01ED01 |
| Ceritinib | L01ED02 |
| Alectinib | L01ED03 |
| Lorlatinib | L01ED05 |
| Palbociclib | L01EF01 |
| Ribociclib | L01EF02 |
| Temsirolimus | L01EG01 |
| Everolimus | L01EG02 |
| Lapatinib | L01EH01 |
| Ruxolitinib | L01EJ01 |
| Axitinib | L01EK01 |
| Ibrutinib | L01EL01 |
| Sunitinib | L01EX01 |
| Sorafenib | L01EX02 |
| Pazopanib | L01EX03 |
| Regorafenib | L01EX05 |
| Cabozantinib | L01EX07 |
| Lenvatinib | L01EX08 |
| Nintedanib | L01EX09 |
| Midostaurin | L01EX10 |
| Rituximab | L01FA01 |
| Obinutuzumab | L01FA03 |
| Daratumumab | L01FC01 |
| Trastuzumab | L01FD01 |
| Pertuzumab | L01FD02 |
| Cetuximab | L01FE01 |
| Panitumumab | L01FE02 |
| Nivolumab | L01FF01 |
| Pembrolizumab | L01FF02 |
| Avelumab | L01FF04 |
| Atezolizumab | L01FF05 |
| Bevacizumab | L01FG01 |
| Brentuximab vedotin | L01FX05 |
| Cisplatin | L01XA01 |
| Carboplatin | L01XA02 |
| Oxaliplatin | L01XA03 |
| L01XC03 | L01XC03 |
| Tretinoin | L01XF01 |
| Bortezomib | L01XG01 |
| Carfilzomib | L01XG02 |
| Ixazomib | L01XG03 |
| Olaparib | L01XK01 |
| Other antineoplastic agents | L01XX |
| Asparaginase | L01XX02 |
| Hydroxycarbamide | L01XX05 |
| Estramustine | L01XX11 |
| Mitotane | L01XX23 |
| Arsenic trioxide | L01XX27 |
| Anagrelide | L01XX35 |
| Eribulin | L01XX41 |
| Diethylstilbestrol | L02AA01 |
| Megestrol | L02AB01 |
| Medroxyprogesterone | L02AB02 |
| Buserelin | L02AE01 |
| Leuprorelin | L02AE02 |
| Goserelin | L02AE03 |
| Triptorelin | L02AE04 |
| Tamoxifen | L02BA01 |
| Toremifene | L02BA02 |
| Flutamide | L02BB01 |
| Bicalutamide | L02BB03 |
| Enzalutamide | L02BB04 |
| Aminoglutethimide | L02BG01 |
| Anastrozole | L02BG03 |
| Letrozole | L02BG04 |
| Exemestane | L02BG06 |
| Degarelix | L02BX02 |
| Abiraterone | L02BX03 |
| Filgrastim | L03AA02 |
| Lenograstim | L03AA10 |
| Pegfilgrastim | L03AA13 |
| Interferon gamma | L03AB03 |
| Interferon alfa-2a | L03AB04 |
| Interferon alfa-2b | L03AB05 |
| Interferon beta-1a | L03AB07 |
| Interferon beta-1b | L03AB08 |
| Peginterferon alfa-2b | L03AB10 |
| Peginterferon alfa-2a | L03AB11 |
| Aldesleukin | L03AC01 |
| BCG vaccine | L03AX03 |
| Glatiramer acetate | L03AX13 |
| Plerixafor | L03AX16 |
| Antilymphocyte immunoglobulin (horse) | L04AA03 |
| Antithymocyte immunoglobulin (rabbit) | L04AA04 |
| Mycophenolic acid | L04AA06 |
| Sirolimus | L04AA10 |
| Leflunomide | L04AA13 |
| Everolimus | L04AA18 |
| Natalizumab | L04AA23 |
| Abatacept | L04AA24 |
| Eculizumab | L04AA25 |
| Fingolimod | L04AA27 |
| Tofacitinib | L04AA29 |
| Teriflunomide | L04AA31 |
| Vedolizumab | L04AA33 |
| Alemtuzumab | L04AA34 |
| Baricitinib | L04AA37 |
| Cladribine | L04AA40 |
| Etanercept | L04AB01 |
| Infliximab | L04AB02 |
| Adalimumab | L04AB04 |
| Certolizumab pegol | L04AB05 |
| Golimumab | L04AB06 |
| Ustekinumab | L04AC05 |
| Tocilizumab | L04AC07 |
| Secukinumab | L04AC10 |
| Brodalumab | L04AC12 |
| Ixekizumab | L04AC13 |
| Guselkumab | L04AC16 |
| Risankizumab | L04AC18 |
| Ciclosporin | L04AD01 |
| Tacrolimus | L04AD02 |
| Azathioprine | L04AX01 |
| Thalidomide | L04AX02 |
| Methotrexate | L04AX03 |
| Lenalidomide | L04AX04 |
| Pirfenidone | L04AX05 |
| Pomalidomide | L04AX06 |
| Dimethyl fumarate | L04AX07 |
| Indometacin | M01AB01 |
| Sulindac | M01AB02 |
| Diclofenac | M01AB05 |
| Etodolac | M01AB08 |
| Acemetacin | M01AB11 |
| Ketorolac | M01AB15 |
| Aceclofenac | M01AB16 |
| Piroxicam | M01AC01 |
| Meloxicam | M01AC06 |
| Ibuprofen | M01AE01 |
| Naproxen | M01AE02 |
| Ketoprofen | M01AE03 |
| Mefenamic acid | M01AG01 |
| Celecoxib | M01AH01 |
| Rofecoxib | M01AH02 |
| Etoricoxib | M01AH05 |
| Nabumetone | M01AX01 |
| Glucosamine | M01AX05 |
| M01AX91 | M01AX91 |
| Penicillamine | M01CC01 |
| Etofenamate | M02AA06 |
| Flurbiprofen | M02AA19 |
| Suxamethonium | M03AB01 |
| Pancuronium | M03AC01 |
| Vecuronium | M03AC03 |
| Atracurium | M03AC04 |
| Cisatracurium | M03AC11 |
| Botulinum toxin | M03AX01 |
| Carisoprodol | M03BA02 |
| Methocarbamol | M03BA03 |
| Baclofen | M03BX01 |
| Tizanidine | M03BX02 |
| Dantrolene | M03CA01 |
| Allopurinol | M04AA01 |
| Febuxostat | M04AA03 |
| Probenecid | M04AB01 |
| Sulfinpyrazone | M04AB02 |
| Benzbromarone | M04AB03 |
| Colchicine | M04AC01 |
| Clodronic acid | M05BA02 |
| Pamidronic acid | M05BA03 |
| Alendronic acid | M05BA04 |
| Ibandronic acid | M05BA06 |
| Risedronic acid | M05BA07 |
| Zoledronic acid | M05BA08 |
| Alendronic acid and colecalciferol | M05BB03 |
| Denosumab | M05BX04 |
| Fentanyl | N01AH01 |
| Alfentanil | N01AH02 |
| Propofol | N01AX10 |
| Lidocaine | N01BB02 |
| Morphine | N02AA01 |
| Opium | N02AA02 |
| Hydromorphone | N02AA03 |
| Oxycodone | N02AA05 |
| Pethidine | N02AB02 |
| Fentanyl | N02AB03 |
| Dextropropoxyphene, combinations excl. Psycholeptics | N02AC54 |
| Buprenorphine | N02AE01 |
| Nalbuphine | N02AF02 |
| Tramadol and paracetamol | N02AJ13 |
| Tramadol | N02AX02 |
| Acetylsalicylic acid | N02BA01 |
| Paracetamol | N02BE01 |
| Ergotamine, combinations excl. Psycholeptics | N02CA52 |
| Sumatriptan | N02CC01 |
| Rizatriptan | N02CC04 |
| Phenobarbital | N03AA02 |
| Phenytoin | N03AB02 |
| Phenytoin, combinations | N03AB52 |
| Clonazepam | N03AE01 |
| Carbamazepine | N03AF01 |
| Oxcarbazepine | N03AF02 |
| Rufinamide | N03AF03 |
| Valproic acid | N03AG01 |
| Vigabatrin | N03AG04 |
| Tiagabine | N03AG06 |
| Lamotrigine | N03AX09 |
| Topiramate | N03AX11 |
| Gabapentin | N03AX12 |
| Levetiracetam | N03AX14 |
| Zonisamide | N03AX15 |
| Pregabalin | N03AX16 |
| Lacosamide | N03AX18 |
| Perampanel | N03AX22 |
| Trihexyphenidyl | N04AA01 |
| Biperiden | N04AA02 |
| Levodopa and decarboxylase inhibitor | N04BA02 |
| Levodopa, decarboxylase inhibitor and COMT inhibitor | N04BA03 |
| Amantadine | N04BB01 |
| Pergolide | N04BC02 |
| Ropinirole | N04BC04 |
| Pramipexole | N04BC05 |
| Apomorphine | N04BC07 |
| Rotigotine | N04BC09 |
| Selegiline | N04BD01 |
| Rasagiline | N04BD02 |
| Entacapone | N04BX02 |
| Chlorpromazine | N05AA01 |
| Fluphenazine | N05AB02 |
| Perphenazine | N05AB03 |
| Prochlorperazine | N05AB04 |
| Trifluoperazine | N05AB06 |
| Thioridazine | N05AC02 |
| Haloperidol | N05AD01 |
| Ziprasidone | N05AE04 |
| Lurasidone | N05AE05 |
| Flupentixol | N05AF01 |
| Tiotixene | N05AF04 |
| Loxapine | N05AH01 |
| Clozapine | N05AH02 |
| Olanzapine | N05AH03 |
| Quetiapine | N05AH04 |
| Sulpiride | N05AL01 |
| Amisulpride | N05AL05 |
| Lithium | N05AN01 |
| Risperidone | N05AX08 |
| Zotepine | N05AX11 |
| Aripiprazole | N05AX12 |
| Paliperidone | N05AX13 |
| Diazepam | N05BA01 |
| Chlordiazepoxide | N05BA02 |
| Lorazepam | N05BA06 |
| Clobazam | N05BA09 |
| Alprazolam | N05BA12 |
| Fludiazepam | N05BA17 |
| Hydroxyzine | N05BB01 |
| Buspirone | N05BE01 |
| Mephenoxalone | N05BX01 |
| Flurazepam | N05CD01 |
| Estazolam | N05CD04 |
| Triazolam | N05CD05 |
| Midazolam | N05CD08 |
| Zolpidem | N05CF02 |
| Zaleplon | N05CF03 |
| Dexmedetomidine | N05CM18 |
| Imipramine | N06AA02 |
| Clomipramine | N06AA04 |
| Amitriptyline | N06AA09 |
| Maprotiline | N06AA21 |
| Fluoxetine | N06AB03 |
| Paroxetine | N06AB05 |
| Sertraline | N06AB06 |
| Escitalopram | N06AB10 |
| Moclobemide | N06AG02 |
| Trazodone | N06AX05 |
| Mirtazapine | N06AX11 |
| Bupropion | N06AX12 |
| Venlafaxine | N06AX16 |
| Duloxetine | N06AX21 |
| Agomelatine | N06AX22 |
| Methylphenidate | N06BA04 |
| Modafinil | N06BA07 |
| Atomoxetine | N06BA09 |
| Piracetam | N06BX03 |
| Donepezil | N06DA02 |
| Rivastigmine | N06DA03 |
| Galantamine | N06DA04 |
| Memantine | N06DX01 |
| Ginkgo folium | N06DX02 |
| Neostigmine | N07AA01 |
| Pyridostigmine | N07AA02 |
| Bethanechol | N07AB02 |
| Pilocarpine | N07AX01 |
| Cevimeline | N07AX03 |
| Antivertigo preparations | N07CA |
| Betahistine | N07CA01 |
| Cinnarizine | N07CA02 |
| Flunarizine | N07CA03 |
| Riluzole | N07XX02 |
| Tetrabenazine | N07XX06 |
| Metronidazole | P01AB01 |
| Tinidazole | P01AB02 |
| Hydroxychloroquine | P01BA02 |
| Praziquantel | P02BA01 |
| Mebendazole | P02CA01 |
| Pyrantel | P02CC01 |
| Levamisole | P02CE01 |
| Ivermectin | P02CF01 |
| Lindane | P03AB02 |
| Permethrin | P03AC04 |
| Oxymetazoline | R01AA05 |
| Cromoglicic acid | R01AC01 |
| Azelastine | R01AC03 |
| Beclometasone | R01AD01 |
| Budesonide | R01AD05 |
| Fluticasone | R01AD08 |
| Mometasone | R01AD09 |
| Triamcinolone | R01AD11 |
| Fluticasone furoate | R01AD12 |
| Pseudoephedrine, combinations | R01BA52 |
| Salbutamol | R03AC02 |
| Terbutaline | R03AC03 |
| Fenoterol | R03AC04 |
| Salmeterol | R03AC12 |
| Formoterol | R03AC13 |
| Indacaterol | R03AC18 |
| Olodaterol | R03AC19 |
| Salmeterol and fluticasone | R03AK06 |
| Formoterol and budesonide | R03AK07 |
| Formoterol and beclometasone | R03AK08 |
| Vilanterol and fluticasone furoate | R03AK10 |
| Salbutamol and ipratropium bromide | R03AL02 |
| Vilanterol and umeclidinium bromide | R03AL03 |
| Indacaterol and glycopyrronium bromide | R03AL04 |
| Olodaterol and tiotropium bromide | R03AL06 |
| Vilanterol, umeclidinium bromide and fluticasone furoate | R03AL08 |
| Formoterol, glycopyrronium bromide and beclometasone | R03AL09 |
| Beclometasone | R03BA01 |
| Budesonide | R03BA02 |
| Fluticasone | R03BA05 |
| Ciclesonide | R03BA08 |
| Ipratropium bromide | R03BB01 |
| Tiotropium bromide | R03BB04 |
| Glycopyrronium bromide | R03BB06 |
| Umeclidinium bromide | R03BB07 |
| Cromoglicic acid | R03BC01 |
| Ephedrine | R03CA02 |
| Orciprenaline, combinations | R03CB53 |
| Salbutamol | R03CC02 |
| Fenoterol | R03CC04 |
| Procaterol | R03CC08 |
| Choline theophyllinate | R03DA02 |
| Theophylline | R03DA04 |
| Aminophylline | R03DA05 |
| Zafirlukast | R03DC01 |
| Montelukast | R03DC03 |
| Omalizumab | R03DX05 |
| Mepolizumab | R03DX09 |
| Benralizumab | R03DX10 |
| Guaifenesin | R05CA03 |
| Combinations | R05CA10 |
| R05CA92 | R05CA92 |
| Acetylcysteine | R05CB01 |
| Bromhexine | R05CB02 |
| Carbocisteine | R05CB03 |
| Eprazinone | R05CB04 |
| Ambroxol | R05CB06 |
| Codeine | R05DA04 |
| Dextromethorphan | R05DA09 |
| Benzonatate | R05DB01 |
| Pentoxyverine | R05DB05 |
| Butamirate | R05DB13 |
| Opium derivatives and expectorants | R05FA |
| Opium derivatives and mucolytics | R05FA01 |
| Opium derivatives and expectorants | R05FA02 |
| OTHER COLD PREPARATIONS | R05X |
| Diphenhydramine | R06AA02 |
| Clemastine | R06AA04 |
| Dexchlorpheniramine | R06AB02 |
| Chlorphenamine | R06AB04 |
| Promethazine | R06AD02 |
| Meclozine | R06AE05 |
| Oxatomide | R06AE06 |
| Cetirizine | R06AE07 |
| Levocetirizine | R06AE09 |
| Cyproheptadine | R06AX02 |
| Loratadine | R06AX13 |
| Ketotifen | R06AX17 |
| Ebastine | R06AX22 |
| Fexofenadine | R06AX26 |
| Desloratadine | R06AX27 |
| Combinations | R07AA30 |
| Chloramphenicol | S01AA01 |
| Tetracycline | S01AA09 |
| Natamycin | S01AA10 |
| Gentamicin | S01AA11 |
| Tobramycin | S01AA12 |
| Combinations of different antibiotics | S01AA30 |
| S01AB91 | S01AB91 |
| Aciclovir | S01AD03 |
| Norfloxacin | S01AE02 |
| Ciprofloxacin | S01AE03 |
| Levofloxacin | S01AE05 |
| Dexamethasone | S01BA01 |
| Prednisolone | S01BA04 |
| Betamethasone | S01BA06 |
| Fluorometholone | S01BA07 |
| Diclofenac | S01BC03 |
| Ketorolac | S01BC05 |
| Dexamethasone and antiinfectives | S01CA01 |
| Prednisolone and antiinfectives | S01CA02 |
| Betamethasone and antiinfectives | S01CA05 |
| Dipivefrine | S01EA02 |
| Brimonidine | S01EA05 |
| Pilocarpine | S01EB01 |
| Neostigmine | S01EB06 |
| Acetazolamide | S01EC01 |
| Dorzolamide | S01EC03 |
| Brinzolamide | S01EC04 |
| Brinzolamide, combinations | S01EC54 |
| Timolol | S01ED01 |
| Betaxolol | S01ED02 |
| Levobunolol | S01ED03 |
| Carteolol | S01ED05 |
| Timolol, combinations | S01ED51 |
| Latanoprost | S01EE01 |
| Bimatoprost | S01EE03 |
| Travoprost | S01EE04 |
| Tafluprost | S01EE05 |
| Atropine | S01FA01 |
| Cyclopentolate | S01FA04 |
| Tropicamide | S01FA06 |
| Phenylephrine | S01FB01 |
| Cromoglicic acid | S01GX01 |
| Emedastine | S01GX06 |
| Ketotifen | S01GX08 |
| Hypromellose | S01KA02 |
| Verteporfin | S01LA01 |
| Ranibizumab | S01LA04 |
| Aflibercept | S01LA05 |
| Other ophthalmologicals | S01XA |
| Ciclosporin | S01XA18 |
| Artificial tears and other indifferent preparations | S01XA20 |
| Ofloxacin | S02AA16 |
| Triamcinolone and antiinfectives | S02CA04 |
| Dexamethasone and antiinfectives | S02CA06 |
| Antidotes | V03AB |
| Pralidoxime | V03AB04 |
| Protamine | V03AB14 |
| Naloxone | V03AB15 |
| Acetylcysteine | V03AB23 |
| Flumazenil | V03AB25 |
| Idarucizumab | V03AB37 |
| V03AB92 | V03AB92 |
| Deferoxamine | V03AC01 |
| Deferiprone | V03AC02 |
| Deferasirox | V03AC03 |
| Polystyrene sulfonate | V03AE01 |
| Calcium acetate | V03AE07 |
| Mesna | V03AF01 |
| Calcium folinate | V03AF03 |
| Rasburicase | V03AF07 |
| Diazoxide | V03AH01 |
| Protirelin | V04CJ02 |
| Amino acids, incl. Combinations with polypeptides | V06DD |
| Radium (223 Ra) dichloride | V10XX03 |
| Description | ATC |
| Chlorhexidine | A01AB03 |
| Triamcinolone | A01AC01 |
| Dexamethasone | A01AC02 |
| Hydrocortisone | A01AC03 |
| A01AC91 | A01AC91 |
| Epinephrine | A01AD01 |
| Magnesium oxide | A02AA02 |
| Aluminium compounds | A02AB |
| Aluminium hydroxide | A02AB01 |
| Ordinary salt combinations | A02AD01 |
| Hydrotalcite | A02AD04 |
| Ordinary salt combinations and antiflatulents | A02AF02 |
| Antacids with antispasmodics | A02AG |
| Antacids with sodium bicarbonate | A02AH |
| Antacids, other combinations | A02AX |
| Cimetidine | A02BA01 |
| Ranitidine | A02BA02 |
| Famotidine | A02BA03 |
| Misoprostol | A02BB01 |
| Omeprazole | A02BC01 |
| Pantoprazole | A02BC02 |
| Lansoprazole | A02BC03 |
| Rabeprazole | A02BC04 |
| Esomeprazole | A02BC05 |
| Dexlansoprazole | A02BC06 |
| Sucralfate | A02BX02 |
| Alginic acid | A02BX13 |
| Mebeverine | A03AA04 |
| Glycopyrronium bromide | A03AB02 |
| Propantheline | A03AB05 |
| Otilonium bromide | A03AB06 |
| Mepenzolate | A03AB12 |
| Papaverine | A03AD01 |
| Pinaverium | A03AX04 |
| Alverine | A03AX08 |
| A03AX95 | A03AX95 |
| Atropine | A03BA01 |
| Butylscopolamine | A03BB01 |
| Metoclopramide | A03FA01 |
| Cisapride | A03FA02 |
| Domperidone | A03FA03 |
| Mosapride | A03FA09 |
| Ondansetron | A04AA01 |
| Granisetron | A04AA02 |

**Supplementary Table 2.** All feature importance ranking results of the 4 different machine learning algorithms

| **Rank** | **30 days** | **90 days** | **180 days** | **365 days** |
| --- | --- | --- | --- | --- |
| 1 | Sleep-wake disorders (after) | Sleep-wake disorders (after) | Sleep-wake disorders (after) | Sleep-wake disorders (after) |
| 2 | Blood urea nitrogen (during) | Blood urea nitrogen (during) | Weight (after) | Weight (after) |
| 3 | Hypertension (after) | DBP (after) | SBP (after) | DBP (after) |
| 4 | Hypertension (before) | Hypertension (after) | Hypertension (after) | SBP (after) |
| 5 | Bleed (before) | DBP (during) | DBP (after) | Hypertension (after) |
| 6 | White blood cell (during) | Weight (after) | Hemiplegia (after) | Hemiplegia (after) |
| 7 | Mean corpuscular hemoglobin concentration (during) | Hypertension (before) | Anxiety disorders (after) | Age (during) |
| 8 | Age (during) | White blood cell (during) | White blood cell-lymphocyte (during) | Red blood cell (during) |
| 9 | DBP (after) | Hemiplegia (after) | Weight (during) | DBP (during) |
| 10 | White blood cell-lymphocyte (during) | Mean corpuscular hemoglobin concentration (during) | Age (during) | Anxiety disorders (after) |
| 11 | Red blood cell (during) | Red blood cell (during) | SBP (during) | Height (after) |
| 12 | Height (during) | Platelet (during) | Hypertension (before) | White blood cell-eosinophil (during) |
| 13 | Potassium (during) | Age (during) | Height (after) | Weight (during) |
| 14 | Anxiety disorders (after) | SBP (after) | Height (during) | Hypertension (before) |
| 15 | Alanine aminotransferase (during) | Serum creatinine (during) | DM (after) | Potassium (during) |
| 16 | Platelet (during) | Weight (during) | Serum creatinine (during) | White blood cell (during) |
| 17 | Sleep-wake disorders (before) | Sleep-wake disorders (before) | Blood urea nitrogen (during) | White blood cell-basophil (during) |
| 18 | Hemiplegia (after) | White blood cell-eosinophil (during) | Sleep-wake disorders (before) | Blood urea nitrogen (during) |
| 19 | Weight (after) | White blood cell-monocyte (during) | White blood cell-eosinophil (during) | Bleed (before) |
| 20 | DM (after) | Natrium (during) | Natrium (during) | Height (during) |
| 21 | Hematocrit (during) | Anxiety disorders (after) | Platelet (during) | Alanine aminotransferase (during) |
| 22 | Serum creatinine (during) | Hematocrit (during) | Hemoglobin (during) | White blood cell-lymphocyte (during) |
| 23 | White blood cell-monocyte (during) | Height (during) | DBP (during) | SBP (during) |
| 24 | SBP (after) | Alanine aminotransferase (during) | White blood cell (during) | Serum creatinine (during) |
| 25 | White blood cell-eosinophil (during) | Height (after) | Potassium (during) | Red cell distribution width-standard deviation (during) |
| 26 | Red cell distribution width-standard deviation (during) | Red cell distribution width-standard deviation (during) | White blood cell-segment (during) | Sleep-wake disorders (before) |
| 27 | DBP (during) | Bleed (before) | Alanine aminotransferase (during) | Platelet (during) |
| 28 | Sex (during) | Hemoglobin (during) | Hemiplegia (before) | Natrium (during) |
| 29 | Weight (during) | Potassium (during) | Red cell distribution width-standard deviation (during) | Tumor (after) |
| 30 | SBP (during) | Mean corpuscular hemoglobin (during) | Red blood cell (during) | White blood cell-segment (during) |
| 31 | White blood cell-basophil (during) | White blood cell-lymphocyte (during) | White blood cell-monocyte (during) | DM (after) |
| 32 | Tumor (before) | White blood cell-basophil (during) | Mean corpuscular hemoglobin concentration (during) | White blood cell-monocyte (during) |
| 33 | Hemoglobin (during) | Mean corpuscular volume (during) | Mean corpuscular volume (during) | Hematocrit (during) |
| 34 | Natrium (during) | White blood cell-segment (during) | DM (before) | Mean corpuscular hemoglobin concentration (during) |
| 35 | Height (after) | Hemiplegia (before) | Hematocrit (during) | Hemiplegia (before) |
| 36 | Mean corpuscular hemoglobin (during) | SBP (during) | Mean corpuscular hemoglobin (during) | Tumor (before) |
| 37 | Mean corpuscular volume (during) | Myocardial infarct (before) | Bleed (before) | Mean corpuscular volume (during) |
| 38 | Heart (before) | Anxiety disorders (before) | Myocardial infarct (before) | Hemoglobin (during) |
| 39 | White blood cell-segment (during) | DM (after) | Tumor (after) | Sex (during) |
| 40 | Chronic pulmonary disease (after) | Schizophrenia spectrum and other psychotic disorders (after) | White blood cell-basophil (during) | Anxiety disorders (before) |
| 41 | Anxiety disorders (before) | DM (before) | Anxiety disorders (before) | Mean corpuscular hemoglobin (during) |
| 42 | Chronic pulmonary disease (before) | Renal disease (after) | Schizophrenia spectrum and other psychotic disorders (after) | Bleed (after) |
| 43 | Substance-related and addictive disorders (after) | Tumor (before) | Liver (after) | Hyperlipidemia (after) |
| 44 | Hyperlipidemia (before) | Tumor (after) | Sex (during) | Chronic pulmonary disease (after) |
| 45 | Liver (before) | Heart (before) | Renal disease (after) | Somatic symptom and related disorders (after) |
| 46 | Bleed (after) | Liver (after) | Chronic pulmonary disease (after) | Schizophrenia spectrum and other psychotic disorders (after) |
| 47 | Schizophrenia spectrum and other psychotic disorders (after) | Somatic symptom and related disorders (after) | Substance-related and addictive disorders (after) | Hyperlipidemia (before) |
| 48 | Hyperlipidemia (after) | Substance-related and addictive disorders (after) | Bleed (after) | Trauma- and stressor-related disorders (after) |
| 49 | DM (before) | Hyperlipidemia (before) | Hyperlipidemia (before) | DM (before) |
| 50 | Myocardial infarct (before) | Chronic pulmonary disease (after) | Tumor (before) | Liver (before) |
| 51 | Hemiplegia (before) | Hyperlipidemia (after) | Somatic symptom and related disorders (after) | Chronic pulmonary disease (before) |
| 52 | Heart (after) | Schizophrenia spectrum and other psychotic disorders (before) | Schizophrenia spectrum and other psychotic disorders (before) | Heart (after) |
| 53 | Renal disease (before) | Heart (after) | Hyperlipidemia (after) | Heart (before) |
| 54 | Liver (after) | Bleed (after) | Trauma- and stressor-related disorders (after) | Substance-related and addictive disorders (after) |
| 55 | Personality disorders (after) | Elimination disorders (before) | Sexual dysfunctions (after) | Myocardial infarct (after) |
| 56 | Myocardial infarct (after) | Sex (during) | Obsessive-compulsive and related disorders (after) | Sexual dysfunctions (after) |
| 57 | Rheumatic disease (before) | Chronic pulmonary disease (before) | Myocardial infarct (after) | Renal disease (after) |
| 58 | Obsessive-compulsive and related disorders (after) | Liver (before) | Heart (before) | Liver (after) |
| 59 | Schizophrenia spectrum and other psychotic disorders (before) | Obsessive-compulsive and related disorders (after) | Renal disease (before) | Elimination disorders (after) |
| 60 | bupropion (after) | Renal disease (before) | Elimination disorders (before) | Obsessive-compulsive and related disorders (after) |
| 61 | Sexual dysfunctions (before) | Trauma- and stressor-related disorders (before) | Liver (before) | Renal disease (before) |
| 62 | toremifene (after) | Rheumatic disease (before) | Chronic pulmonary disease (before) | Myocardial infarct (before) |
| 63 | enzalutamide (after) | Myocardial infarct (after) | Elimination disorders (after) | Trauma- and stressor-related disorders (before) |
| 64 | triptorelin (after) | Obsessive-compulsive and related disorders (before) | Substance-related and addictive disorders (before) | Sexual dysfunctions (before) |
| 65 | goserelin (after) | Sexual dysfunctions (after) | levamisole (after) | Neurodevelopmental disorders (after) |
| 66 | leuprorelin (after) | levamisole (after) | Rheumatic disease (before) | Substance-related and addictive disorders (before) |
| 67 | flutamide (after) | Personality disorders (after) | Rheumatic disease (after) | Parahilic disorders (before) |
| 68 | buserelin (after) | Somatic symptom and related disorders (before) | degarelix (after) | leuprorelin (after) |
| 69 | medroxyprogesterone (after) | Sexual dysfunctions (before) | exemestane (after) | medroxyprogesterone (after) |
| 70 | bicalutamide (after) | letrozole (after) | letrozole (after) | buserelin (after) |
| 71 | megestrol (after) | abiraterone (after) | anastrozole (after) | letrozole (after) |
| 72 | tamoxifen (after) | degarelix (after) | L01XC03 (after) | flutamide (after) |
| 73 | avelumab (after) | exemestane (after) | aminoglutethimide (after) | anastrozole (after) |
| 74 | aminoglutethimide (after) | carfilzomib (after) | enzalutamide (after) | aminoglutethimide (after) |
| 75 | interferon alfa-2b (after) | anastrozole (after) | bicalutamide (after) | goserelin (after) |
| 76 | antilymphocyte immunoglobulin (horse) (after) | aminoglutethimide (after) | flutamide (after) | triptorelin (after) |
| 77 | plerixafor (after) | enzalutamide (after) | toremifene (after) | enzalutamide (after) |
| 78 | glatiramer acetate (after) | flutamide (after) | triptorelin (after) | bicalutamide (after) |
| 79 | BCG vaccine (after) | toremifene (after) | goserelin (after) | tamoxifen (after) |
| 80 | aldesleukin (after) | tamoxifen (after) | leuprorelin (after) | toremifene (after) |
| 81 | peginterferon alfa-2a (after) | triptorelin (after) | buserelin (after) | exemestane (after) |
| 82 | peginterferon alfa-2b (after) | goserelin (after) | medroxyprogesterone (after) | pegfilgrastim (after) |
| 83 | interferon beta-1b (after) | leuprorelin (after) | tamoxifen (after) | degarelix (after) |
| 84 | interferon beta-1a (after) | buserelin (after) | pegfilgrastim (after) | abiraterone (after) |
| 85 | interferon alfa-2a (after) | bicalutamide (after) | abiraterone (after) | mycophenolic acid (after) |
| 86 | anastrozole (after) | pegfilgrastim (after) | filgrastim (after) | antithymocyte immunoglobulin (rabbit) (after) |
| 87 | interferon gamma (after) | filgrastim (after) | antithymocyte immunoglobulin (rabbit) (after) | antilymphocyte immunoglobulin (horse) (after) |
| 88 | pegfilgrastim (after) | lenograstim (after) | antilymphocyte immunoglobulin (horse) (after) | plerixafor (after) |
| 89 | lenograstim (after) | mycophenolic acid (after) | plerixafor (after) | glatiramer acetate (after) |
| 90 | filgrastim (after) | antithymocyte immunoglobulin (rabbit) (after) | glatiramer acetate (after) | BCG vaccine (after) |
| 91 | abiraterone (after) | antilymphocyte immunoglobulin (horse) (after) | BCG vaccine (after) | aldesleukin (after) |
| 92 | eribulin (after) | plerixafor (after) | aldesleukin (after) | peginterferon alfa-2a (after) |
| 93 | degarelix (after) | glatiramer acetate (after) | peginterferon alfa-2a (after) | peginterferon alfa-2b (after) |
| 94 | exemestane (after) | BCG vaccine (after) | peginterferon alfa-2b (after) | interferon beta-1b (after) |
| 95 | letrozole (after) | aldesleukin (after) | interferon beta-1b (after) | interferon beta-1a (after) |
| 96 | diethylstilbestrol (after) | peginterferon alfa-2a (after) | interferon beta-1a (after) | interferon alfa-2b (after) |
| 97 | midostaurin (after) | peginterferon alfa-2b (after) | interferon alfa-2b (after) | interferon alfa-2a (after) |
| 98 | anagrelide (after) | interferon beta-1b (after) | interferon alfa-2a (after) | interferon gamma (after) |
| 99 | arsenic trioxide (after) | interferon beta-1a (after) | interferon gamma (after) | diethylstilbestrol (after) |
| 100 | nivolumab (after) | interferon alfa-2b (after) | diethylstilbestrol (after) | lenograstim (after) |
| 101 | asparaginase (after) | interferon alfa-2a (after) | lenograstim (after) | filgrastim (after) |
| 102 | Other antineoplastic agents (after) | interferon gamma (after) | megestrol (after) | megestrol (after) |
| 103 | olaparib (after) | megestrol (after) | lenvatinib (after) | nintedanib (after) |
| 104 | pembrolizumab (after) | medroxyprogesterone (after) | eribulin (after) | eribulin (after) |
| 105 | ixazomib (after) | estramustine (after) | bevacizumab (after) | anagrelide (after) |
| 106 | carfilzomib (after) | diethylstilbestrol (after) | panitumumab (after) | nivolumab (after) |
| 107 | bortezomib (after) | leflunomide (after) | nivolumab (after) | leflunomide (after) |
| 108 | tretinoin (after) | cetuximab (after) | pembrolizumab (after) | pembrolizumab (after) |
| 109 | L01XC03 (after) | panitumumab (after) | sirolimus (after) | avelumab (after) |
| 110 | oxaliplatin (after) | nivolumab (after) | avelumab (after) | atezolizumab (after) |
| 111 | carboplatin (after) | pembrolizumab (after) | atezolizumab (after) | bevacizumab (after) |
| 112 | cisplatin (after) | avelumab (after) | brentuximab vedotin (after) | brentuximab vedotin (after) |
| 113 | brentuximab vedotin (after) | atezolizumab (after) | pertuzumab (after) | cisplatin (after) |
| 114 | bevacizumab (after) | bevacizumab (after) | cisplatin (after) | ixazomib (after) |
| 115 | panitumumab (after) | trastuzumab (after) | carboplatin (after) | carfilzomib (after) |
| 116 | hydroxycarbamide (after) | brentuximab vedotin (after) | oxaliplatin (after) | carboplatin (after) |
| 117 | cetuximab (after) | cisplatin (after) | ixazomib (after) | bortezomib (after) |
| 118 | lenvatinib (after) | carboplatin (after) | carfilzomib (after) | tretinoin (after) |
| 119 | mitotane (after) | oxaliplatin (after) | bortezomib (after) | L01XC03 (after) |
| 120 | estramustine (after) | L01XC03 (after) | cetuximab (after) | oxaliplatin (after) |
| 121 | sorafenib (after) | tretinoin (after) | trastuzumab (after) | panitumumab (after) |
| 122 | pazopanib (after) | pertuzumab (after) | anagrelide (after) | cetuximab (after) |
| 123 | regorafenib (after) | daratumumab (after) | Other antineoplastic agents (after) | pertuzumab (after) |
| 124 | cabozantinib (after) | eribulin (after) | arsenic trioxide (after) | Other antineoplastic agents (after) |
| 125 | nintedanib (after) | asparaginase (after) | mitotane (after) | arsenic trioxide (after) |
| 126 | mycophenolic acid (after) | anagrelide (after) | estramustine (after) | mitotane (after) |
| 127 | atezolizumab (after) | arsenic trioxide (after) | hydroxycarbamide (after) | regorafenib (after) |
| 128 | rituximab (after) | mitotane (after) | regorafenib (after) | estramustine (after) |
| 129 | obinutuzumab (after) | cabozantinib (after) | asparaginase (after) | hydroxycarbamide (after) |
| 130 | daratumumab (after) | bortezomib (after) | olaparib (after) | asparaginase (after) |
| 131 | trastuzumab (after) | hydroxycarbamide (after) | daratumumab (after) | olaparib (after) |
| 132 | pertuzumab (after) | Other antineoplastic agents (after) | cabozantinib (after) | trastuzumab (after) |
| 133 | antithymocyte immunoglobulin (rabbit) (after) | obinutuzumab (after) | tretinoin (after) | cabozantinib (after) |
| 134 | methotrexate (after) | olaparib (after) | nintedanib (after) | lenvatinib (after) |
| 135 | sirolimus (after) | ixazomib (after) | midostaurin (after) | midostaurin (after) |
| 136 | etodolac (after) | lenvatinib (after) | rituximab (after) | rituximab (after) |
| 137 | penicillamine (after) | nintedanib (after) | obinutuzumab (after) | obinutuzumab (after) |
| 138 | M01AX91 (after) | midostaurin (after) | mycophenolic acid (after) | daratumumab (after) |
| 139 | glucosamine (after) | rituximab (after) | dimethyl fumarate (after) | sirolimus (after) |
| 140 | nabumetone (after) | sirolimus (after) | leflunomide (after) | sulindac (after) |
| 141 | etoricoxib (after) | sulindac (after) | acemetacin (after) | everolimus (after) |
| 142 | rofecoxib (after) | everolimus (after) | etofenamate (after) | ketorolac (after) |
| 143 | celecoxib (after) | ketorolac (after) | penicillamine (after) | flurbiprofen (after) |
| 144 | mefenamic acid (after) | flurbiprofen (after) | M01AX91 (after) | etofenamate (after) |
| 145 | ketoprofen (after) | etofenamate (after) | glucosamine (after) | penicillamine (after) |
| 146 | naproxen (after) | penicillamine (after) | nabumetone (after) | M01AX91 (after) |
| 147 | ibuprofen (after) | M01AX91 (after) | etoricoxib (after) | glucosamine (after) |
| 148 | meloxicam (after) | glucosamine (after) | rofecoxib (after) | nabumetone (after) |
| 149 | piroxicam (after) | nabumetone (after) | celecoxib (after) | etoricoxib (after) |
| 150 | aceclofenac (after) | etoricoxib (after) | mefenamic acid (after) | rofecoxib (after) |
| 151 | ketorolac (after) | rofecoxib (after) | ketoprofen (after) | celecoxib (after) |
| 152 | etofenamate (after) | celecoxib (after) | naproxen (after) | mefenamic acid (after) |
| 153 | flurbiprofen (after) | mefenamic acid (after) | ibuprofen (after) | ketoprofen (after) |
| 154 | suxamethonium (after) | ketoprofen (after) | meloxicam (after) | naproxen (after) |
| 155 | tizanidine (after) | naproxen (after) | piroxicam (after) | ibuprofen (after) |
| 156 | benzbromarone (after) | ibuprofen (after) | aceclofenac (after) | meloxicam (after) |
| 157 | sulfinpyrazone (after) | meloxicam (after) | flurbiprofen (after) | piroxicam (after) |
| 158 | probenecid (after) | piroxicam (after) | suxamethonium (after) | suxamethonium (after) |
| 159 | febuxostat (after) | suxamethonium (after) | pancuronium (after) | pancuronium (after) |
| 160 | allopurinol (after) | pancuronium (after) | dantrolene (after) | vecuronium (after) |
| 161 | dantrolene (after) | vecuronium (after) | colchicine (after) | allopurinol (after) |
| 162 | baclofen (after) | allopurinol (after) | benzbromarone (after) | clodronic acid (after) |
| 163 | pancuronium (after) | clodronic acid (after) | sulfinpyrazone (after) | colchicine (after) |
| 164 | methocarbamol (after) | colchicine (after) | probenecid (after) | benzbromarone (after) |
| 165 | carisoprodol (after) | benzbromarone (after) | febuxostat (after) | sulfinpyrazone (after) |
| 166 | botulinum toxin (after) | sulfinpyrazone (after) | allopurinol (after) | probenecid (after) |
| 167 | cisatracurium (after) | probenecid (after) | tizanidine (after) | febuxostat (after) |
| 168 | atracurium (after) | febuxostat (after) | vecuronium (after) | dantrolene (after) |
| 169 | vecuronium (after) | dantrolene (after) | baclofen (after) | atracurium (after) |
| 170 | acemetacin (after) | atracurium (after) | methocarbamol (after) | tizanidine (after) |
| 171 | diclofenac (after) | tizanidine (after) | carisoprodol (after) | baclofen (after) |
| 172 | leflunomide (after) | baclofen (after) | botulinum toxin (after) | methocarbamol (after) |
| 173 | sulindac (after) | methocarbamol (after) | cisatracurium (after) | carisoprodol (after) |
| 174 | certolizumab pegol (after) | carisoprodol (after) | atracurium (after) | botulinum toxin (after) |
| 175 | adalimumab (after) | botulinum toxin (after) | ketorolac (after) | cisatracurium (after) |
| 176 | infliximab (after) | cisatracurium (after) | etodolac (after) | aceclofenac (after) |
| 177 | etanercept (after) | aceclofenac (after) | everolimus (after) | acemetacin (after) |
| 178 | cladribine (after) | acemetacin (after) | diclofenac (after) | natalizumab (after) |
| 179 | baricitinib (after) | natalizumab (after) | golimumab (after) | etodolac (after) |
| 180 | alemtuzumab (after) | etodolac (after) | certolizumab pegol (after) | ustekinumab (after) |
| 181 | vedolizumab (after) | ustekinumab (after) | adalimumab (after) | golimumab (after) |
| 182 | teriflunomide (after) | golimumab (after) | infliximab (after) | certolizumab pegol (after) |
| 183 | tofacitinib (after) | certolizumab pegol (after) | etanercept (after) | adalimumab (after) |
| 184 | fingolimod (after) | adalimumab (after) | cladribine (after) | infliximab (after) |
| 185 | eculizumab (after) | infliximab (after) | baricitinib (after) | etanercept (after) |
| 186 | abatacept (after) | etanercept (after) | alemtuzumab (after) | cladribine (after) |
| 187 | natalizumab (after) | cladribine (after) | vedolizumab (after) | baricitinib (after) |
| 188 | everolimus (after) | baricitinib (after) | teriflunomide (after) | alemtuzumab (after) |
| 189 | golimumab (after) | alemtuzumab (after) | tofacitinib (after) | vedolizumab (after) |
| 190 | ustekinumab (after) | vedolizumab (after) | fingolimod (after) | teriflunomide (after) |
| 191 | tocilizumab (after) | teriflunomide (after) | eculizumab (after) | tofacitinib (after) |
| 192 | thalidomide (after) | tofacitinib (after) | abatacept (after) | fingolimod (after) |
| 193 | indometacin (after) | fingolimod (after) | natalizumab (after) | eculizumab (after) |
| 194 | dimethyl fumarate (after) | eculizumab (after) | ustekinumab (after) | abatacept (after) |
| 195 | pomalidomide (after) | abatacept (after) | tocilizumab (after) | tocilizumab (after) |
| 196 | pirfenidone (after) | tocilizumab (after) | secukinumab (after) | secukinumab (after) |
| 197 | lenalidomide (after) | secukinumab (after) | methotrexate (after) | brodalumab (after) |
| 198 | ibrutinib (after) | brodalumab (after) | sulindac (after) | lenalidomide (after) |
| 199 | azathioprine (after) | lenalidomide (after) | indometacin (after) | diclofenac (after) |
| 200 | secukinumab (after) | diclofenac (after) | sorafenib (after) | sorafenib (after) |
| 201 | tacrolimus (after) | pazopanib (after) | pomalidomide (after) | indometacin (after) |
| 202 | ciclosporin (after) | indometacin (after) | pirfenidone (after) | dimethyl fumarate (after) |
| 203 | risankizumab (after) | dimethyl fumarate (after) | lenalidomide (after) | pomalidomide (after) |
| 204 | guselkumab (after) | pomalidomide (after) | thalidomide (after) | pirfenidone (after) |
| 205 | ixekizumab (after) | pirfenidone (after) | brodalumab (after) | methotrexate (after) |
| 206 | brodalumab (after) | methotrexate (after) | azathioprine (after) | ixekizumab (after) |
| 207 | sunitinib (after) | ixekizumab (after) | tacrolimus (after) | thalidomide (after) |
| 208 | topotecan (after) | thalidomide (after) | ciclosporin (after) | azathioprine (after) |
| 209 | axitinib (after) | azathioprine (after) | risankizumab (after) | tacrolimus (after) |
| 210 | asunaprevir (after) | tacrolimus (after) | guselkumab (after) | ciclosporin (after) |
| 211 | Antivirals for treatment of HCV infections (after) | ciclosporin (after) | ixekizumab (after) | risankizumab (after) |
| 212 | dolutegravir (after) | risankizumab (after) | pazopanib (after) | guselkumab (after) |
| 213 | raltegravir (after) | guselkumab (after) | docetaxel (after) | pazopanib (after) |
| 214 | oseltamivir (after) | regorafenib (after) | sunitinib (after) | paclitaxel (after) |
| 215 | rilpivirine (after) | docetaxel (after) | abacavir (after) | sunitinib (after) |
| 216 | etravirine (after) | sorafenib (after) | daclatasvir (after) | abacavir (after) |
| 217 | efavirenz (after) | tenofovir disoproxil (after) | asunaprevir (after) | daclatasvir (after) |
| 218 | nevirapine (after) | sofosbuvir (after) | ribavirin (after) | asunaprevir (after) |
| 219 | tenofovir alafenamide (after) | daclatasvir (after) | Antivirals for treatment of HCV infections (after) | ribavirin (after) |
| 220 | telbivudine (after) | asunaprevir (after) | dolutegravir (after) | Antivirals for treatment of HCV infections (after) |
| 221 | entecavir (after) | ribavirin (after) | raltegravir (after) | dolutegravir (after) |
| 222 | adefovir dipivoxil (after) | Antivirals for treatment of HCV infections (after) | oseltamivir (after) | raltegravir (after) |
| 223 | tenofovir disoproxil (after) | dolutegravir (after) | rilpivirine (after) | oseltamivir (after) |
| 224 | abacavir (after) | raltegravir (after) | etravirine (after) | rilpivirine (after) |
| 225 | lamivudine (after) | oseltamivir (after) | efavirenz (after) | etravirine (after) |
| 226 | ribavirin (after) | rilpivirine (after) | nevirapine (after) | efavirenz (after) |
| 227 | daclatasvir (after) | etravirine (after) | tenofovir alafenamide (after) | nevirapine (after) |
| 228 | ruxolitinib (after) | efavirenz (after) | telbivudine (after) | tenofovir alafenamide (after) |
| 229 | sofosbuvir (after) | nevirapine (after) | entecavir (after) | telbivudine (after) |
| 230 | emtricitabine, tenofovir alafenamide, elvitegravir and cobicistat (after) | tenofovir alafenamide (after) | adefovir dipivoxil (after) | entecavir (after) |
| 231 | darunavir and cobicistat (after) | telbivudine (after) | sofosbuvir (after) | adefovir dipivoxil (after) |
| 232 | lamivudine, abacavir and dolutegravir (after) | entecavir (after) | dasabuvir (after) | sofosbuvir (after) |
| 233 | lopinavir and ritonavir (after) | dasabuvir (after) | sofosbuvir and ledipasvir (after) | dasabuvir (after) |
| 234 | emtricitabine, tenofovir disoproxil and rilpivirine (after) | sofosbuvir and ledipasvir (after) | emtricitabine, tenofovir disoproxil and rilpivirine (after) | sofosbuvir and ledipasvir (after) |
| 235 | emtricitabine, tenofovir disoproxil and efavirenz (after) | ombitasvir, paritaprevir and ritonavir (after) | emtricitabine, tenofovir alafenamide and bictegravir (after) | emtricitabine, tenofovir disoproxil and rilpivirine (after) |
| 236 | tenofovir disoproxil and emtricitabine (after) | lopinavir and ritonavir (after) | emtricitabine, tenofovir alafenamide and rilpivirine (after) | emtricitabine, tenofovir alafenamide and bictegravir (after) |
| 237 | lamivudine and abacavir (after) | dolutegravir and rilpivirine (after) | emtricitabine, tenofovir alafenamide, elvitegravir and cobicistat (after) | emtricitabine, tenofovir alafenamide and rilpivirine (after) |
| 238 | zidovudine and lamivudine (after) | emtricitabine, tenofovir alafenamide and bictegravir (after) | darunavir and cobicistat (after) | emtricitabine, tenofovir alafenamide, elvitegravir and cobicistat (after) |
| 239 | glecaprevir and pibrentasvir (after) | emtricitabine, tenofovir alafenamide and rilpivirine (after) | lamivudine, abacavir and dolutegravir (after) | darunavir and cobicistat (after) |
| 240 | sofosbuvir and velpatasvir (after) | emtricitabine, tenofovir alafenamide, elvitegravir and cobicistat (after) | lopinavir and ritonavir (after) | lamivudine, abacavir and dolutegravir (after) |
| 241 | elbasvir and grazoprevir (after) | darunavir and cobicistat (after) | emtricitabine, tenofovir disoproxil and efavirenz (after) | lopinavir and ritonavir (after) |
| 242 | ombitasvir, paritaprevir and ritonavir (after) | lamivudine, abacavir and dolutegravir (after) | ombitasvir, paritaprevir and ritonavir (after) | emtricitabine, tenofovir disoproxil and efavirenz (after) |
| 243 | sofosbuvir and ledipasvir (after) | emtricitabine, tenofovir disoproxil and rilpivirine (after) | tenofovir disoproxil and emtricitabine (after) | ombitasvir, paritaprevir and ritonavir (after) |
| 244 | dasabuvir (after) | elbasvir and grazoprevir (after) | lamivudine and abacavir (after) | tenofovir disoproxil and emtricitabine (after) |
| 245 | stavudine (after) | emtricitabine, tenofovir disoproxil and efavirenz (after) | zidovudine and lamivudine (after) | lamivudine and abacavir (after) |
| 246 | didanosine (after) | tenofovir disoproxil and emtricitabine (after) | glecaprevir and pibrentasvir (after) | zidovudine and lamivudine (after) |
| 247 | zidovudine (after) | lamivudine and abacavir (after) | sofosbuvir and velpatasvir (after) | glecaprevir and pibrentasvir (after) |
| 248 | darunavir (after) | zidovudine and lamivudine (after) | elbasvir and grazoprevir (after) | sofosbuvir and velpatasvir (after) |
| 249 | calcium aminosalicylate (after) | glecaprevir and pibrentasvir (after) | tenofovir disoproxil (after) | elbasvir and grazoprevir (after) |
| 250 | anidulafungin (after) | sofosbuvir and velpatasvir (after) | lamivudine (after) | tenofovir disoproxil (after) |
| 251 | micafungin (after) | adefovir dipivoxil (after) | emtricitabine, tenofovir alafenamide, darunavir and cobicistat (after) | lamivudine (after) |
| 252 | caspofungin (after) | abacavir (after) | stavudine (after) | emtricitabine, tenofovir alafenamide, darunavir and cobicistat (after) |
| 253 | flucytosine (after) | letermovir (after) | rifamycin (after) | stavudine (after) |
| 254 | posaconazole (after) | lamivudine (after) | rifampicin (after) | rifamycin (after) |
| 255 | voriconazole (after) | rifabutin (after) | cycloserine (after) | rifampicin (after) |
| 256 | itraconazole (after) | rifamycin (after) | calcium aminosalicylate (after) | cycloserine (after) |
| 257 | fluconazole (after) | rifampicin (after) | anidulafungin (after) | calcium aminosalicylate (after) |
| 258 | ketoconazole (after) | cycloserine (after) | micafungin (after) | anidulafungin (after) |
| 259 | amphotericin B (after) | calcium aminosalicylate (after) | caspofungin (after) | micafungin (after) |
| 260 | daptomycin (after) | anidulafungin (after) | flucytosine (after) | caspofungin (after) |
| 261 | linezolid (after) | micafungin (after) | posaconazole (after) | flucytosine (after) |
| 262 | fosfomycin (after) | caspofungin (after) | voriconazole (after) | posaconazole (after) |
| 263 | nitrofurantoin (after) | flucytosine (after) | itraconazole (after) | voriconazole (after) |
| 264 | cycloserine (after) | posaconazole (after) | fluconazole (after) | itraconazole (after) |
| 265 | rifampicin (after) | voriconazole (after) | ketoconazole (after) | fluconazole (after) |
| 266 | rifamycin (after) | itraconazole (after) | amphotericin B (after) | ketoconazole (after) |
| 267 | aciclovir (after) | fluconazole (after) | daptomycin (after) | amphotericin B (after) |
| 268 | atazanavir (after) | ketoconazole (after) | rifabutin (after) | daptomycin (after) |
| 269 | ritonavir (after) | amphotericin B (after) | isoniazid (after) | rifabutin (after) |
| 270 | valganciclovir (after) | isoniazid (after) | pyrazinamide (after) | isoniazid (after) |
| 271 | valaciclovir (after) | pyrazinamide (after) | valaciclovir (after) | pyrazinamide (after) |
| 272 | famciclovir (after) | ethambutol (after) | didanosine (after) | valaciclovir (after) |
| 273 | ganciclovir (after) | valganciclovir (after) | zidovudine (after) | didanosine (after) |
| 274 | rifampicin, ethambutol and isoniazid (after) | stavudine (after) | darunavir (after) | zidovudine (after) |
| 275 | rifabutin (after) | didanosine (after) | atazanavir (after) | darunavir (after) |
| 276 | rifampicin, pyrazinamide, ethambutol and isoniazid (after) | zidovudine (after) | ritonavir (after) | atazanavir (after) |
| 277 | rifampicin, pyrazinamide and isoniazid (after) | darunavir (after) | valganciclovir (after) | ritonavir (after) |
| 278 | rifampicin and isoniazid (after) | atazanavir (after) | famciclovir (after) | valganciclovir (after) |
| 279 | ethambutol (after) | ritonavir (after) | ethambutol (after) | famciclovir (after) |
| 280 | pyrazinamide (after) | valaciclovir (after) | ganciclovir (after) | ethambutol (after) |
| 281 | isoniazid (after) | rifampicin and isoniazid (after) | aciclovir (after) | ganciclovir (after) |
| 282 | emtricitabine, tenofovir alafenamide and rilpivirine (after) | famciclovir (after) | rifampicin, ethambutol and isoniazid (after) | aciclovir (after) |
| 283 | emtricitabine, tenofovir alafenamide and bictegravir (after) | ganciclovir (after) | rifampicin, pyrazinamide, ethambutol and isoniazid (after) | rifampicin, ethambutol and isoniazid (after) |
| 284 | dolutegravir and rilpivirine (after) | aciclovir (after) | rifampicin, pyrazinamide and isoniazid (after) | rifampicin, pyrazinamide, ethambutol and isoniazid (after) |
| 285 | trifluridine, combinations (after) | rifampicin, ethambutol and isoniazid (after) | rifampicin and isoniazid (after) | rifampicin, pyrazinamide and isoniazid (after) |
| 286 | mitomycin (after) | rifampicin, pyrazinamide, ethambutol and isoniazid (after) | dolutegravir and rilpivirine (after) | rifampicin and isoniazid (after) |
| 287 | bleomycin (after) | rifampicin, pyrazinamide and isoniazid (after) | letermovir (after) | dolutegravir and rilpivirine (after) |
| 288 | mitoxantrone (after) | emtricitabine, tenofovir alafenamide, darunavir and cobicistat (after) | ibrutinib (after) | letermovir (after) |
| 289 | idarubicin (after) | tetanus antitoxin (after) | vincristine (after) | ibrutinib (after) |
| 290 | epirubicin (after) | sunitinib (after) | dasatinib (after) | vincristine (after) |
| 291 | daunorubicin (after) | vinorelbine (after) | imatinib (after) | dasatinib (after) |
| 292 | doxorubicin (after) | nilotinib (after) | mitomycin (after) | imatinib (after) |
| 293 | dactinomycin (after) | dasatinib (after) | bleomycin (after) | mitomycin (after) |
| 294 | irinotecan (after) | imatinib (after) | mitoxantrone (after) | bleomycin (after) |
| 295 | clodronic acid (after) | mitomycin (after) | idarubicin (after) | mitoxantrone (after) |
| 296 | docetaxel (after) | bleomycin (after) | epirubicin (after) | idarubicin (after) |
| 297 | paclitaxel (after) | mitoxantrone (after) | daunorubicin (after) | epirubicin (after) |
| 298 | etoposide (after) | idarubicin (after) | doxorubicin (after) | daunorubicin (after) |
| 299 | vinorelbine (after) | epirubicin (after) | dactinomycin (after) | doxorubicin (after) |
| 300 | vincristine (after) | daunorubicin (after) | irinotecan (after) | dactinomycin (after) |
| 301 | imatinib (after) | doxorubicin (after) | topotecan (after) | irinotecan (after) |
| 302 | dasatinib (after) | dactinomycin (after) | pamidronic acid (after) | topotecan (after) |
| 303 | nilotinib (after) | irinotecan (after) | paclitaxel (after) | docetaxel (after) |
| 304 | alectinib (after) | topotecan (after) | etoposide (after) | alendronic acid (after) |
| 305 | lapatinib (after) | alendronic acid (after) | nilotinib (after) | etoposide (after) |
| 306 | everolimus (after) | paclitaxel (after) | ponatinib (after) | nilotinib (after) |
| 307 | temsirolimus (after) | ponatinib (after) | gefitinib (after) | ponatinib (after) |
| 308 | ribociclib (after) | gefitinib (after) | palbociclib (after) | gefitinib (after) |
| 309 | palbociclib (after) | erlotinib (after) | axitinib (after) | palbociclib (after) |
| 310 | lorlatinib (after) | ribociclib (after) | ruxolitinib (after) | axitinib (after) |
| 311 | ceritinib (after) | ibrutinib (after) | lapatinib (after) | ruxolitinib (after) |
| 312 | ponatinib (after) | axitinib (after) | everolimus (after) | lapatinib (after) |
| 313 | crizotinib (after) | ruxolitinib (after) | temsirolimus (after) | everolimus (after) |
| 314 | vemurafenib (after) | lapatinib (after) | ribociclib (after) | temsirolimus (after) |
| 315 | osimertinib (after) | everolimus (after) | lorlatinib (after) | ribociclib (after) |
| 316 | afatinib (after) | temsirolimus (after) | erlotinib (after) | lorlatinib (after) |
| 317 | erlotinib (after) | palbociclib (after) | alectinib (after) | erlotinib (after) |
| 318 | gefitinib (after) | afatinib (after) | ceritinib (after) | alectinib (after) |
| 319 | vinblastine (after) | lorlatinib (after) | crizotinib (after) | ceritinib (after) |
| 320 | tegafur, combinations (after) | alectinib (after) | vemurafenib (after) | crizotinib (after) |
| 321 | emtricitabine, tenofovir alafenamide, darunavir and cobicistat (after) | ceritinib (after) | osimertinib (after) | vemurafenib (after) |
| 322 | azacitidine (after) | crizotinib (after) | afatinib (after) | osimertinib (after) |
| 323 | melphalan (after) | vemurafenib (after) | vinorelbine (after) | afatinib (after) |
| 324 | chlorambucil (after) | osimertinib (after) | vinblastine (after) | vinorelbine (after) |
| 325 | cyclophosphamide (after) | etoposide (after) | tetanus antitoxin (after) | vinblastine (after) |
| 326 | rabies, inactivated, whole virus (after) | vincristine (after) | trifluridine, combinations (after) | tetanus antitoxin (after) |
| 327 | tetanus toxoid (after) | snake venom antiserum (after) | bendamustine (after) | trifluridine, combinations (after) |
| 328 | palivizumab (after) | vinblastine (after) | ifosfamide (after) | bendamustine (after) |
| 329 | cytomegalovirus immunoglobulin (after) | busulfan (after) | melphalan (after) | ifosfamide (after) |
| 330 | hepatitis B immunoglobulin (after) | bendamustine (after) | chlorambucil (after) | melphalan (after) |
| 331 | tetanus immunoglobulin (after) | ifosfamide (after) | cyclophosphamide (after) | chlorambucil (after) |
| 332 | anti-D (rh) immunoglobulin (after) | melphalan (after) | rabies, inactivated, whole virus (after) | cyclophosphamide (after) |
| 333 | J06BA03 (after) | chlorambucil (after) | tetanus toxoid (after) | rabies, inactivated, whole virus (after) |
| 334 | immunoglobulins, normal human, for intravascular adm. (after) | cyclophosphamide (after) | palivizumab (after) | tetanus toxoid (after) |
| 335 | snake venom antiserum (after) | rabies, inactivated, whole virus (after) | cytomegalovirus immunoglobulin (after) | palivizumab (after) |
| 336 | tetanus antitoxin (after) | tetanus toxoid (after) | hepatitis B immunoglobulin (after) | cytomegalovirus immunoglobulin (after) |
| 337 | letermovir (after) | palivizumab (after) | tetanus immunoglobulin (after) | hepatitis B immunoglobulin (after) |
| 338 | ifosfamide (after) | cytomegalovirus immunoglobulin (after) | anti-D (rh) immunoglobulin (after) | tetanus immunoglobulin (after) |
| 339 | bendamustine (after) | hepatitis B immunoglobulin (after) | J06BA03 (after) | anti-D (rh) immunoglobulin (after) |
| 340 | busulfan (after) | tetanus immunoglobulin (after) | immunoglobulins, normal human, for intravascular adm. (after) | J06BA03 (after) |
| 341 | cladribine (after) | anti-D (rh) immunoglobulin (after) | snake venom antiserum (after) | immunoglobulins, normal human, for intravascular adm. (after) |
| 342 | capecitabine (after) | J06BA03 (after) | busulfan (after) | snake venom antiserum (after) |
| 343 | gemcitabine (after) | immunoglobulins, normal human, for intravascular adm. (after) | carmustine (after) | busulfan (after) |
| 344 | tegafur (after) | carmustine (after) | temozolomide (after) | carmustine (after) |
| 345 | fluorouracil (after) | temozolomide (after) | cytarabine (after) | temozolomide (after) |
| 346 | cytarabine (after) | dacarbazine (after) | tegafur, combinations (after) | cytarabine (after) |
| 347 | fludarabine (after) | fluorouracil (after) | azacitidine (after) | tegafur, combinations (after) |
| 348 | tioguanine (after) | trifluridine, combinations (after) | capecitabine (after) | azacitidine (after) |
| 349 | carmustine (after) | tegafur, combinations (after) | gemcitabine (after) | capecitabine (after) |
| 350 | mercaptopurine (after) | azacitidine (after) | tegafur (after) | gemcitabine (after) |
| 351 | pralatrexate (after) | capecitabine (after) | fluorouracil (after) | tegafur (after) |
| 352 | pemetrexed (after) | gemcitabine (after) | fludarabine (after) | fluorouracil (after) |
| 353 | methotrexate (after) | tegafur (after) | dacarbazine (after) | fludarabine (after) |
| 354 | dacarbazine (after) | cytarabine (after) | cladribine (after) | dacarbazine (after) |
| 355 | temozolomide (after) | methotrexate (after) | tioguanine (after) | cladribine (after) |
| 356 | colchicine (after) | fludarabine (after) | mercaptopurine (after) | tioguanine (after) |
| 357 | phenytoin, combinations (after) | cladribine (after) | pralatrexate (after) | mercaptopurine (after) |
| 358 | pamidronic acid (after) | tioguanine (after) | pemetrexed (after) | pralatrexate (after) |
| 359 | opium derivatives and mucolytics (after) | mercaptopurine (after) | methotrexate (after) | pemetrexed (after) |
| 360 | butamirate (after) | pralatrexate (after) | clodronic acid (after) | methotrexate (after) |
| 361 | pentoxyverine (after) | pemetrexed (after) | rufinamide (after) | pamidronic acid (after) |
| 362 | benzonatate (after) | pamidronic acid (after) | alendronic acid (after) | valproic acid (after) |
| 363 | dextromethorphan (after) | vigabatrin (after) | omalizumab (after) | ibandronic acid (after) |
| 364 | codeine (after) | ibandronic acid (after) | Opium derivatives and expectorants (after) | omalizumab (after) |
| 365 | ambroxol (after) | omalizumab (after) | butamirate (after) | Opium derivatives and expectorants (after) |
| 366 | eprazinone (after) | Opium derivatives and expectorants (after) | pentoxyverine (after) | butamirate (after) |
| 367 | carbocisteine (after) | butamirate (after) | benzonatate (after) | pentoxyverine (after) |
| 368 | bromhexine (after) | pentoxyverine (after) | dextromethorphan (after) | benzonatate (after) |
| 369 | acetylcysteine (after) | benzonatate (after) | codeine (after) | dextromethorphan (after) |
| 370 | R05CA92 (after) | dextromethorphan (after) | ambroxol (after) | codeine (after) |
| 371 | combinations (after) | codeine (after) | eprazinone (after) | ambroxol (after) |
| 372 | guaifenesin (after) | ambroxol (after) | carbocisteine (after) | eprazinone (after) |
| 373 | benralizumab (after) | eprazinone (after) | bromhexine (after) | carbocisteine (after) |
| 374 | mepolizumab (after) | carbocisteine (after) | acetylcysteine (after) | bromhexine (after) |
| 375 | Opium derivatives and expectorants (after) | bromhexine (after) | R05CA92 (after) | acetylcysteine (after) |
| 376 | opium derivatives and expectorants (after) | acetylcysteine (after) | combinations (after) | R05CA92 (after) |
| 377 | alendronic acid (after) | R05CA92 (after) | guaifenesin (after) | combinations (after) |
| 378 | OTHER COLD PREPARATIONS (after) | combinations (after) | benralizumab (after) | guaifenesin (after) |
| 379 | desloratadine (after) | guaifenesin (after) | opium derivatives and mucolytics (after) | benralizumab (after) |
| 380 | fexofenadine (after) | benralizumab (after) | opium derivatives and expectorants (after) | opium derivatives and mucolytics (after) |
| 381 | ebastine (after) | opium derivatives and mucolytics (after) | OTHER COLD PREPARATIONS (after) | opium derivatives and expectorants (after) |
| 382 | ketotifen (after) | opium derivatives and expectorants (after) | levocetirizine (after) | OTHER COLD PREPARATIONS (after) |
| 383 | loratadine (after) | OTHER COLD PREPARATIONS (after) | desloratadine (after) | levocetirizine (after) |
| 384 | cyproheptadine (after) | levocetirizine (after) | fexofenadine (after) | desloratadine (after) |
| 385 | levocetirizine (after) | desloratadine (after) | ebastine (after) | fexofenadine (after) |
| 386 | cetirizine (after) | fexofenadine (after) | ketotifen (after) | ebastine (after) |
| 387 | oxatomide (after) | ebastine (after) | loratadine (after) | ketotifen (after) |
| 388 | meclozine (after) | ketotifen (after) | cyproheptadine (after) | loratadine (after) |
| 389 | promethazine (after) | loratadine (after) | cetirizine (after) | cyproheptadine (after) |
| 390 | chlorphenamine (after) | cyproheptadine (after) | diphenhydramine (after) | cetirizine (after) |
| 391 | dexchlorpheniramine (after) | cetirizine (after) | oxatomide (after) | diphenhydramine (after) |
| 392 | clemastine (after) | diphenhydramine (after) | meclozine (after) | oxatomide (after) |
| 393 | diphenhydramine (after) | oxatomide (after) | promethazine (after) | meclozine (after) |
| 394 | omalizumab (after) | meclozine (after) | chlorphenamine (after) | promethazine (after) |
| 395 | montelukast (after) | promethazine (after) | dexchlorpheniramine (after) | chlorphenamine (after) |
| 396 | zafirlukast (after) | chlorphenamine (after) | clemastine (after) | dexchlorpheniramine (after) |
| 397 | aminophylline (after) | dexchlorpheniramine (after) | mepolizumab (after) | clemastine (after) |
| 398 | olodaterol and tiotropium bromide (after) | clemastine (after) | montelukast (after) | mepolizumab (after) |
| 399 | indacaterol and glycopyrronium bromide (after) | mepolizumab (after) | chloramphenicol (after) | montelukast (after) |
| 400 | vilanterol and umeclidinium bromide (after) | montelukast (after) | zafirlukast (after) | chloramphenicol (after) |
| 401 | salbutamol and ipratropium bromide (after) | chloramphenicol (after) | vilanterol, umeclidinium bromide and fluticasone furoate (after) | zafirlukast (after) |
| 402 | vilanterol and fluticasone furoate (after) | zafirlukast (after) | olodaterol and tiotropium bromide (after) | vilanterol, umeclidinium bromide and fluticasone furoate (after) |
| 403 | formoterol and beclometasone (after) | vilanterol, umeclidinium bromide and fluticasone furoate (after) | indacaterol and glycopyrronium bromide (after) | olodaterol and tiotropium bromide (after) |
| 404 | formoterol and budesonide (after) | olodaterol and tiotropium bromide (after) | vilanterol and umeclidinium bromide (after) | indacaterol and glycopyrronium bromide (after) |
| 405 | salmeterol and fluticasone (after) | indacaterol and glycopyrronium bromide (after) | salbutamol and ipratropium bromide (after) | vilanterol and umeclidinium bromide (after) |
| 406 | olodaterol (after) | vilanterol and umeclidinium bromide (after) | vilanterol and fluticasone furoate (after) | salbutamol and ipratropium bromide (after) |
| 407 | indacaterol (after) | salbutamol and ipratropium bromide (after) | formoterol and beclometasone (after) | vilanterol and fluticasone furoate (after) |
| 408 | formoterol (after) | vilanterol and fluticasone furoate (after) | formoterol and budesonide (after) | formoterol and beclometasone (after) |
| 409 | salmeterol (after) | formoterol and beclometasone (after) | salmeterol and fluticasone (after) | formoterol and budesonide (after) |
| 410 | fenoterol (after) | formoterol and budesonide (after) | olodaterol (after) | salmeterol and fluticasone (after) |
| 411 | terbutaline (after) | salmeterol and fluticasone (after) | indacaterol (after) | olodaterol (after) |
| 412 | salbutamol (after) | olodaterol (after) | formoterol (after) | indacaterol (after) |
| 413 | vilanterol, umeclidinium bromide and fluticasone furoate (after) | indacaterol (after) | salmeterol (after) | formoterol (after) |
| 414 | formoterol, glycopyrronium bromide and beclometasone (after) | formoterol (after) | fenoterol (after) | salmeterol (after) |
| 415 | beclometasone (after) | salmeterol (after) | terbutaline (after) | fenoterol (after) |
| 416 | ephedrine (after) | fenoterol (after) | formoterol, glycopyrronium bromide and beclometasone (after) | terbutaline (after) |
| 417 | theophylline (after) | terbutaline (after) | beclometasone (after) | formoterol, glycopyrronium bromide and beclometasone (after) |
| 418 | choline theophyllinate (after) | formoterol, glycopyrronium bromide and beclometasone (after) | budesonide (after) | beclometasone (after) |
| 419 | procaterol (after) | beclometasone (after) | orciprenaline, combinations (after) | budesonide (after) |
| 420 | fenoterol (after) | budesonide (after) | aminophylline (after) | orciprenaline, combinations (after) |
| 421 | salbutamol (after) | orciprenaline, combinations (after) | theophylline (after) | aminophylline (after) |
| 422 | orciprenaline, combinations (after) | aminophylline (after) | choline theophyllinate (after) | theophylline (after) |
| 423 | cromoglicic acid (after) | theophylline (after) | procaterol (after) | choline theophyllinate (after) |
| 424 | budesonide (after) | choline theophyllinate (after) | fenoterol (after) | procaterol (after) |
| 425 | umeclidinium bromide (after) | procaterol (after) | salbutamol (after) | fenoterol (after) |
| 426 | glycopyrronium bromide (after) | fenoterol (after) | ephedrine (after) | salbutamol (after) |
| 427 | tiotropium bromide (after) | salbutamol (after) | fluticasone (after) | ephedrine (after) |
| 428 | ipratropium bromide (after) | ephedrine (after) | cromoglicic acid (after) | fluticasone (after) |
| 429 | ciclesonide (after) | fluticasone (after) | umeclidinium bromide (after) | cromoglicic acid (after) |
| 430 | fluticasone (after) | cromoglicic acid (after) | glycopyrronium bromide (after) | umeclidinium bromide (after) |
| 431 | combinations (after) | umeclidinium bromide (after) | tiotropium bromide (after) | glycopyrronium bromide (after) |
| 432 | chloramphenicol (after) | glycopyrronium bromide (after) | ipratropium bromide (after) | tiotropium bromide (after) |
| 433 | tetracycline (after) | tiotropium bromide (after) | ciclesonide (after) | ipratropium bromide (after) |
| 434 | cyclopentolate (after) | ipratropium bromide (after) | combinations (after) | ciclesonide (after) |
| 435 | Antidotes (after) | ciclesonide (after) | tetracycline (after) | combinations (after) |
| 436 | dexamethasone and antiinfectives (after) | combinations (after) | pseudoephedrine, combinations (after) | tetracycline (after) |
| 437 | triamcinolone and antiinfectives (after) | tetracycline (after) | cyclopentolate (after) | pseudoephedrine, combinations (after) |
| 438 | ofloxacin (after) | risedronic acid (after) | Antidotes (after) | cyclopentolate (after) |
| 439 | artificial tears and other indifferent preparations (after) | cyclopentolate (after) | dexamethasone and antiinfectives (after) | Antidotes (after) |
| 440 | ciclosporin (after) | Antidotes (after) | triamcinolone and antiinfectives (after) | dexamethasone and antiinfectives (after) |
| 441 | Other ophthalmologicals (after) | dexamethasone and antiinfectives (after) | ofloxacin (after) | triamcinolone and antiinfectives (after) |
| 442 | aflibercept (after) | triamcinolone and antiinfectives (after) | artificial tears and other indifferent preparations (after) | ofloxacin (after) |
| 443 | ranibizumab (after) | ofloxacin (after) | ciclosporin (after) | artificial tears and other indifferent preparations (after) |
| 444 | verteporfin (after) | artificial tears and other indifferent preparations (after) | Other ophthalmologicals (after) | ciclosporin (after) |
| 445 | hypromellose (after) | ciclosporin (after) | aflibercept (after) | Other ophthalmologicals (after) |
| 446 | ketotifen (after) | Other ophthalmologicals (after) | ranibizumab (after) | aflibercept (after) |
| 447 | emedastine (after) | aflibercept (after) | verteporfin (after) | ranibizumab (after) |
| 448 | cromoglicic acid (after) | ranibizumab (after) | hypromellose (after) | verteporfin (after) |
| 449 | phenylephrine (after) | verteporfin (after) | ketotifen (after) | hypromellose (after) |
| 450 | pralidoxime (after) | hypromellose (after) | emedastine (after) | ketotifen (after) |
| 451 | protamine (after) | ketotifen (after) | cromoglicic acid (after) | emedastine (after) |
| 452 | naloxone (after) | emedastine (after) | phenylephrine (after) | cromoglicic acid (after) |
| 453 | calcium acetate (after) | cromoglicic acid (after) | pralidoxime (after) | phenylephrine (after) |
| 454 | Amino acids, incl. combinations with polypeptides (after) | phenylephrine (after) | protamine (after) | pralidoxime (after) |
| 455 | protirelin (after) | pralidoxime (after) | naloxone (after) | protamine (after) |
| 456 | diazoxide (after) | protamine (after) | calcium acetate (after) | naloxone (after) |
| 457 | rasburicase (after) | naloxone (after) | Amino acids, incl. combinations with polypeptides (after) | calcium acetate (after) |
| 458 | calcium folinate (after) | calcium acetate (after) | protirelin (after) | Amino acids, incl. combinations with polypeptides (after) |
| 459 | mesna (after) | Amino acids, incl. combinations with polypeptides (after) | diazoxide (after) | protirelin (after) |
| 460 | polystyrene sulfonate (after) | protirelin (after) | rasburicase (after) | diazoxide (after) |
| 461 | acetylcysteine (after) | diazoxide (after) | calcium folinate (after) | rasburicase (after) |
| 462 | deferasirox (after) | rasburicase (after) | mesna (after) | calcium folinate (after) |
| 463 | deferiprone (after) | calcium folinate (after) | polystyrene sulfonate (after) | mesna (after) |
| 464 | deferoxamine (after) | mesna (after) | acetylcysteine (after) | polystyrene sulfonate (after) |
| 465 | V03AB92 (after) | polystyrene sulfonate (after) | deferasirox (after) | acetylcysteine (after) |
| 466 | idarucizumab (after) | acetylcysteine (after) | deferiprone (after) | deferasirox (after) |
| 467 | flumazenil (after) | deferasirox (after) | deferoxamine (after) | deferiprone (after) |
| 468 | tropicamide (after) | deferiprone (after) | V03AB92 (after) | deferoxamine (after) |
| 469 | atropine (after) | deferoxamine (after) | idarucizumab (after) | V03AB92 (after) |
| 470 | natamycin (after) | V03AB92 (after) | flumazenil (after) | idarucizumab (after) |
| 471 | tafluprost (after) | idarucizumab (after) | tropicamide (after) | flumazenil (after) |
| 472 | dexamethasone and antiinfectives (after) | flumazenil (after) | atropine (after) | tropicamide (after) |
| 473 | ketorolac (after) | tropicamide (after) | natamycin (after) | atropine (after) |
| 474 | diclofenac (after) | atropine (after) | tafluprost (after) | natamycin (after) |
| 475 | fluorometholone (after) | natamycin (after) | dexamethasone and antiinfectives (after) | tafluprost (after) |
| 476 | betamethasone (after) | tafluprost (after) | ketorolac (after) | dexamethasone and antiinfectives (after) |
| 477 | prednisolone (after) | dexamethasone and antiinfectives (after) | diclofenac (after) | ketorolac (after) |
| 478 | dexamethasone (after) | ketorolac (after) | fluorometholone (after) | diclofenac (after) |
| 479 | levofloxacin (after) | diclofenac (after) | betamethasone (after) | fluorometholone (after) |
| 480 | ciprofloxacin (after) | fluorometholone (after) | prednisolone (after) | betamethasone (after) |
| 481 | norfloxacin (after) | betamethasone (after) | dexamethasone (after) | prednisolone (after) |
| 482 | aciclovir (after) | prednisolone (after) | levofloxacin (after) | dexamethasone (after) |
| 483 | S01AB91 (after) | dexamethasone (after) | ciprofloxacin (after) | levofloxacin (after) |
| 484 | combinations of different antibiotics (after) | levofloxacin (after) | norfloxacin (after) | ciprofloxacin (after) |
| 485 | tobramycin (after) | ciprofloxacin (after) | aciclovir (after) | norfloxacin (after) |
| 486 | gentamicin (after) | norfloxacin (after) | S01AB91 (after) | aciclovir (after) |
| 487 | prednisolone and antiinfectives (after) | aciclovir (after) | combinations of different antibiotics (after) | S01AB91 (after) |
| 488 | betamethasone and antiinfectives (after) | S01AB91 (after) | tobramycin (after) | combinations of different antibiotics (after) |
| 489 | dipivefrine (after) | combinations of different antibiotics (after) | gentamicin (after) | tobramycin (after) |
| 490 | betaxolol (after) | tobramycin (after) | prednisolone and antiinfectives (after) | gentamicin (after) |
| 491 | travoprost (after) | gentamicin (after) | betamethasone and antiinfectives (after) | prednisolone and antiinfectives (after) |
| 492 | bimatoprost (after) | prednisolone and antiinfectives (after) | dipivefrine (after) | betamethasone and antiinfectives (after) |
| 493 | latanoprost (after) | betamethasone and antiinfectives (after) | betaxolol (after) | dipivefrine (after) |
| 494 | timolol, combinations (after) | dipivefrine (after) | travoprost (after) | betaxolol (after) |
| 495 | carteolol (after) | betaxolol (after) | bimatoprost (after) | travoprost (after) |
| 496 | levobunolol (after) | travoprost (after) | latanoprost (after) | bimatoprost (after) |
| 497 | timolol (after) | bimatoprost (after) | timolol, combinations (after) | latanoprost (after) |
| 498 | brimonidine (after) | latanoprost (after) | carteolol (after) | timolol, combinations (after) |
| 499 | brinzolamide, combinations (after) | timolol, combinations (after) | levobunolol (after) | carteolol (after) |
| 500 | brinzolamide (after) | carteolol (after) | timolol (after) | levobunolol (after) |
| 501 | dorzolamide (after) | levobunolol (after) | brimonidine (after) | timolol (after) |
| 502 | acetazolamide (after) | timolol (after) | brinzolamide, combinations (after) | brimonidine (after) |
| 503 | neostigmine (after) | brimonidine (after) | brinzolamide (after) | brinzolamide, combinations (after) |
| 504 | pilocarpine (after) | brinzolamide, combinations (after) | dorzolamide (after) | brinzolamide (after) |
| 505 | pseudoephedrine, combinations (after) | brinzolamide (after) | acetazolamide (after) | dorzolamide (after) |
| 506 | fluticasone furoate (after) | dorzolamide (after) | neostigmine (after) | acetazolamide (after) |
| 507 | triamcinolone (after) | acetazolamide (after) | pilocarpine (after) | neostigmine (after) |
| 508 | lamotrigine (after) | neostigmine (after) | salbutamol (after) | pilocarpine (after) |
| 509 | apomorphine (after) | pilocarpine (after) | fluticasone furoate (after) | salbutamol (after) |
| 510 | pramipexole (after) | salbutamol (after) | ibandronic acid (after) | fluticasone furoate (after) |
| 511 | ropinirole (after) | pseudoephedrine, combinations (after) | topiramate (after) | risedronic acid (after) |
| 512 | pergolide (after) | fluticasone furoate (after) | rotigotine (after) | gabapentin (after) |
| 513 | amantadine (after) | gabapentin (after) | apomorphine (after) | selegiline (after) |
| 514 | levodopa, decarboxylase inhibitor and COMT inhibitor (after) | selegiline (after) | pramipexole (after) | rotigotine (after) |
| 515 | levodopa and decarboxylase inhibitor (after) | rotigotine (after) | ropinirole (after) | apomorphine (after) |
| 516 | biperiden (after) | apomorphine (after) | pergolide (after) | pramipexole (after) |
| 517 | trihexyphenidyl (after) | pramipexole (after) | amantadine (after) | ropinirole (after) |
| 518 | perampanel (after) | ropinirole (after) | levodopa, decarboxylase inhibitor and COMT inhibitor (after) | pergolide (after) |
| 519 | lacosamide (after) | pergolide (after) | levodopa and decarboxylase inhibitor (after) | amantadine (after) |
| 520 | pregabalin (after) | amantadine (after) | biperiden (after) | levodopa, decarboxylase inhibitor and COMT inhibitor (after) |
| 521 | zonisamide (after) | levodopa, decarboxylase inhibitor and COMT inhibitor (after) | trihexyphenidyl (after) | levodopa and decarboxylase inhibitor (after) |
| 522 | levetiracetam (after) | levodopa and decarboxylase inhibitor (after) | perampanel (after) | biperiden (after) |
| 523 | gabapentin (after) | biperiden (after) | lacosamide (after) | trihexyphenidyl (after) |
| 524 | rotigotine (after) | trihexyphenidyl (after) | pregabalin (after) | perampanel (after) |
| 525 | selegiline (after) | perampanel (after) | zonisamide (after) | lacosamide (after) |
| 526 | rasagiline (after) | lacosamide (after) | levetiracetam (after) | pregabalin (after) |
| 527 | ziprasidone (after) | pregabalin (after) | selegiline (after) | zonisamide (after) |
| 528 | olanzapine (after) | zonisamide (after) | rasagiline (after) | rasagiline (after) |
| 529 | clozapine (after) | rasagiline (after) | entacapone (after) | entacapone (after) |
| 530 | loxapine (after) | entacapone (after) | lurasidone (after) | chlorpromazine (after) |
| 531 | tiotixene (after) | chlorpromazine (after) | quetiapine (after) | flupentixol (after) |
| 532 | flupentixol (after) | flupentixol (after) | olanzapine (after) | sulpiride (after) |
| 533 | lurasidone (after) | sulpiride (after) | clozapine (after) | quetiapine (after) |
| 534 | haloperidol (after) | quetiapine (after) | loxapine (after) | olanzapine (after) |
| 535 | entacapone (after) | olanzapine (after) | tiotixene (after) | clozapine (after) |
| 536 | thioridazine (after) | clozapine (after) | flupentixol (after) | loxapine (after) |
| 537 | trifluoperazine (after) | loxapine (after) | ziprasidone (after) | tiotixene (after) |
| 538 | prochlorperazine (after) | tiotixene (after) | chlorpromazine (after) | lurasidone (after) |
| 539 | perphenazine (after) | lurasidone (after) | haloperidol (after) | fluphenazine (after) |
| 540 | fluphenazine (after) | fluphenazine (after) | thioridazine (after) | ziprasidone (after) |
| 541 | chlorpromazine (after) | ziprasidone (after) | trifluoperazine (after) | haloperidol (after) |
| 542 | topiramate (after) | haloperidol (after) | prochlorperazine (after) | thioridazine (after) |
| 543 | tiagabine (after) | thioridazine (after) | perphenazine (after) | trifluoperazine (after) |
| 544 | sulpiride (after) | trifluoperazine (after) | fluphenazine (after) | prochlorperazine (after) |
| 545 | vigabatrin (after) | prochlorperazine (after) | gabapentin (after) | perphenazine (after) |
| 546 | fentanyl (after) | perphenazine (after) | lamotrigine (after) | levetiracetam (after) |
| 547 | pethidine (after) | levetiracetam (after) | amisulpride (after) | topiramate (after) |
| 548 | oxycodone (after) | topiramate (after) | tiagabine (after) | lithium (after) |
| 549 | hydromorphone (after) | triamcinolone (after) | dextropropoxyphene, combinations excl. psycholeptics (after) | lamotrigine (after) |
| 550 | opium (after) | lamotrigine (after) | fentanyl (after) | buprenorphine (after) |
| 551 | morphine (after) | buprenorphine (after) | pethidine (after) | dextropropoxyphene, combinations excl. psycholeptics (after) |
| 552 | lidocaine (after) | dextropropoxyphene, combinations excl. psycholeptics (after) | oxycodone (after) | fentanyl (after) |
| 553 | propofol (after) | fentanyl (after) | hydromorphone (after) | pethidine (after) |
| 554 | alfentanil (after) | pethidine (after) | opium (after) | oxycodone (after) |
| 555 | fentanyl (after) | oxycodone (after) | morphine (after) | hydromorphone (after) |
| 556 | denosumab (after) | hydromorphone (after) | lidocaine (after) | opium (after) |
| 557 | alendronic acid and colecalciferol (after) | opium (after) | propofol (after) | morphine (after) |
| 558 | zoledronic acid (after) | morphine (after) | alfentanil (after) | lidocaine (after) |
| 559 | risedronic acid (after) | lidocaine (after) | fentanyl (after) | propofol (after) |
| 560 | ibandronic acid (after) | propofol (after) | denosumab (after) | alfentanil (after) |
| 561 | dextropropoxyphene, combinations excl. psycholeptics (after) | alfentanil (after) | alendronic acid and colecalciferol (after) | fentanyl (after) |
| 562 | buprenorphine (after) | fentanyl (after) | zoledronic acid (after) | denosumab (after) |
| 563 | nalbuphine (after) | denosumab (after) | risedronic acid (after) | alendronic acid and colecalciferol (after) |
| 564 | phenytoin (after) | alendronic acid and colecalciferol (after) | buprenorphine (after) | zoledronic acid (after) |
| 565 | valproic acid (after) | zoledronic acid (after) | nalbuphine (after) | nalbuphine (after) |
| 566 | rufinamide (after) | nalbuphine (after) | tramadol and paracetamol (after) | tramadol and paracetamol (after) |
| 567 | oxcarbazepine (after) | tramadol and paracetamol (after) | phenytoin, combinations (after) | tramadol (after) |
| 568 | carbamazepine (after) | tramadol (after) | vigabatrin (after) | clonazepam (after) |
| 569 | clonazepam (after) | clonazepam (after) | valproic acid (after) | tiagabine (after) |
| 570 | fusidic acid (after) | tiagabine (after) | fosfomycin (after) | vigabatrin (after) |
| 571 | phenobarbital (after) | linezolid (after) | oxcarbazepine (after) | fosfomycin (after) |
| 572 | tramadol and paracetamol (after) | valproic acid (after) | carbamazepine (after) | rufinamide (after) |
| 573 | rizatriptan (after) | rufinamide (after) | clonazepam (after) | oxcarbazepine (after) |
| 574 | sumatriptan (after) | oxcarbazepine (after) | phenytoin (after) | carbamazepine (after) |
| 575 | ergotamine, combinations excl. psycholeptics (after) | carbamazepine (after) | tramadol (after) | phenytoin, combinations (after) |
| 576 | paracetamol (after) | phenytoin, combinations (after) | phenobarbital (after) | acetylsalicylic acid (after) |
| 577 | acetylsalicylic acid (after) | acetylsalicylic acid (after) | rizatriptan (after) | phenytoin (after) |
| 578 | tramadol (after) | phenytoin (after) | sumatriptan (after) | phenobarbital (after) |
| 579 | quetiapine (after) | phenobarbital (after) | ergotamine, combinations excl. psycholeptics (after) | rizatriptan (after) |
| 580 | amisulpride (after) | rizatriptan (after) | paracetamol (after) | sumatriptan (after) |
| 581 | mometasone (after) | sumatriptan (after) | acetylsalicylic acid (after) | ergotamine, combinations excl. psycholeptics (after) |
| 582 | modafinil (after) | ergotamine, combinations excl. psycholeptics (after) | sulpiride (after) | paracetamol (after) |
| 583 | flunarizine (after) | paracetamol (after) | lithium (after) | amisulpride (after) |
| 584 | cinnarizine (after) | amisulpride (after) | triamcinolone (after) | risperidone (after) |
| 585 | betahistine (after) | lithium (after) | modafinil (after) | triamcinolone (after) |
| 586 | Antivertigo preparations (after) | risperidone (after) | flunarizine (after) | atomoxetine (after) |
| 587 | cevimeline (after) | zotepine (after) | cinnarizine (after) | riluzole (after) |
| 588 | pilocarpine (after) | flunarizine (after) | betahistine (after) | flunarizine (after) |
| 589 | bethanechol (after) | cinnarizine (after) | Antivertigo preparations (after) | cinnarizine (after) |
| 590 | pyridostigmine (after) | betahistine (after) | cevimeline (after) | betahistine (after) |
| 591 | neostigmine (after) | Antivertigo preparations (after) | pilocarpine (after) | Antivertigo preparations (after) |
| 592 | Ginkgo folium (after) | cevimeline (after) | bethanechol (after) | cevimeline (after) |
| 593 | memantine (after) | pilocarpine (after) | pyridostigmine (after) | pilocarpine (after) |
| 594 | galantamine (after) | bethanechol (after) | neostigmine (after) | bethanechol (after) |
| 595 | rivastigmine (after) | pyridostigmine (after) | Ginkgo folium (after) | pyridostigmine (after) |
| 596 | donepezil (after) | neostigmine (after) | memantine (after) | neostigmine (after) |
| 597 | piracetam (after) | Ginkgo folium (after) | galantamine (after) | Ginkgo folium (after) |
| 598 | riluzole (after) | memantine (after) | rivastigmine (after) | memantine (after) |
| 599 | tetrabenazine (after) | galantamine (after) | donepezil (after) | galantamine (after) |
| 600 | metronidazole (after) | rivastigmine (after) | piracetam (after) | rivastigmine (after) |
| 601 | permethrin (after) | donepezil (after) | riluzole (after) | donepezil (after) |
| 602 | fluticasone (after) | piracetam (after) | tetrabenazine (after) | tetrabenazine (after) |
| 603 | budesonide (after) | riluzole (after) | metronidazole (after) | metronidazole (after) |
| 604 | beclometasone (after) | tetrabenazine (after) | oxymetazoline (after) | tinidazole (after) |
| 605 | azelastine (after) | metronidazole (after) | mometasone (after) | oxymetazoline (after) |
| 606 | cromoglicic acid (after) | oxymetazoline (after) | fluticasone (after) | mometasone (after) |
| 607 | oxymetazoline (after) | mometasone (after) | budesonide (after) | fluticasone (after) |
| 608 | lindane (after) | fluticasone (after) | beclometasone (after) | budesonide (after) |
| 609 | tinidazole (after) | budesonide (after) | azelastine (after) | beclometasone (after) |
| 610 | ivermectin (after) | beclometasone (after) | cromoglicic acid (after) | azelastine (after) |
| 611 | levamisole (after) | azelastine (after) | permethrin (after) | cromoglicic acid (after) |
| 612 | pyrantel (after) | cromoglicic acid (after) | tinidazole (after) | permethrin (after) |
| 613 | mebendazole (after) | permethrin (after) | lindane (after) | hydroxychloroquine (after) |
| 614 | praziquantel (after) | tinidazole (after) | ivermectin (after) | lindane (after) |
| 615 | hydroxychloroquine (after) | lindane (after) | pyrantel (after) | ivermectin (after) |
| 616 | atomoxetine (after) | ivermectin (after) | mebendazole (after) | levamisole (after) |
| 617 | methylphenidate (after) | pyrantel (after) | praziquantel (after) | pyrantel (after) |
| 618 | lithium (after) | mebendazole (after) | hydroxychloroquine (after) | mebendazole (after) |
| 619 | agomelatine (after) | praziquantel (after) | atomoxetine (after) | praziquantel (after) |
| 620 | estazolam (after) | hydroxychloroquine (after) | methylphenidate (after) | piracetam (after) |
| 621 | flurazepam (after) | atomoxetine (after) | risperidone (after) | modafinil (after) |
| 622 | mephenoxalone (after) | modafinil (after) | agomelatine (after) | zotepine (after) |
| 623 | buspirone (after) | methylphenidate (after) | triazolam (after) | methylphenidate (after) |
| 624 | hydroxyzine (after) | fludiazepam (after) | estazolam (after) | midazolam (after) |
| 625 | fludiazepam (after) | triazolam (after) | flurazepam (after) | triazolam (after) |
| 626 | alprazolam (after) | estazolam (after) | mephenoxalone (after) | estazolam (after) |
| 627 | clobazam (after) | flurazepam (after) | buspirone (after) | flurazepam (after) |
| 628 | lorazepam (after) | mephenoxalone (after) | hydroxyzine (after) | mephenoxalone (after) |
| 629 | chlordiazepoxide (after) | buspirone (after) | fludiazepam (after) | buspirone (after) |
| 630 | diazepam (after) | hydroxyzine (after) | alprazolam (after) | hydroxyzine (after) |
| 631 | paliperidone (after) | alprazolam (after) | clobazam (after) | fludiazepam (after) |
| 632 | aripiprazole (after) | zolpidem (after) | lorazepam (after) | alprazolam (after) |
| 633 | zotepine (after) | clobazam (after) | chlordiazepoxide (after) | clobazam (after) |
| 634 | risperidone (after) | lorazepam (after) | diazepam (after) | lorazepam (after) |
| 635 | triazolam (after) | chlordiazepoxide (after) | paliperidone (after) | chlordiazepoxide (after) |
| 636 | midazolam (after) | diazepam (after) | aripiprazole (after) | diazepam (after) |
| 637 | zolpidem (after) | paliperidone (after) | zotepine (after) | paliperidone (after) |
| 638 | sertraline (after) | aripiprazole (after) | midazolam (after) | aripiprazole (after) |
| 639 | duloxetine (after) | midazolam (after) | zolpidem (after) | zolpidem (after) |
| 640 | venlafaxine (after) | zaleplon (after) | zaleplon (after) | zaleplon (after) |
| 641 | mirtazapine (after) | agomelatine (after) | escitalopram (after) | dexmedetomidine (after) |
| 642 | trazodone (after) | escitalopram (after) | duloxetine (after) | moclobemide (after) |
| 643 | moclobemide (after) | duloxetine (after) | venlafaxine (after) | agomelatine (after) |
| 644 | escitalopram (after) | venlafaxine (after) | bupropion (after) | duloxetine (after) |
| 645 | paroxetine (after) | bupropion (after) | mirtazapine (after) | venlafaxine (after) |
| 646 | zaleplon (after) | mirtazapine (after) | trazodone (after) | bupropion (after) |
| 647 | fluoxetine (after) | trazodone (after) | moclobemide (after) | mirtazapine (after) |
| 648 | maprotiline (after) | moclobemide (after) | sertraline (after) | trazodone (after) |
| 649 | amitriptyline (after) | sertraline (after) | dexmedetomidine (after) | escitalopram (after) |
| 650 | clomipramine (after) | dexmedetomidine (after) | paroxetine (after) | imipramine (after) |
| 651 | imipramine (after) | paroxetine (after) | fluoxetine (after) | sertraline (after) |
| 652 | dexmedetomidine (after) | fluoxetine (after) | maprotiline (after) | paroxetine (after) |
| 653 | metronidazole (after) | maprotiline (after) | amitriptyline (after) | fluoxetine (after) |
| 654 | ceftazidime and beta-lactamase inhibitor (after) | amitriptyline (after) | clomipramine (after) | maprotiline (after) |
| 655 | colistin (after) | clomipramine (after) | imipramine (after) | amitriptyline (after) |
| 656 | tirofiban (after) | imipramine (after) | linezolid (after) | clomipramine (after) |
| 657 | dipyridamole (after) | daptomycin (after) | ceftazidime and beta-lactamase inhibitor (after) | linezolid (after) |
| 658 | acetylsalicylic acid (after) | ceftazidime and beta-lactamase inhibitor (after) | nitrofurantoin (after) | ceftazidime and beta-lactamase inhibitor (after) |
| 659 | ticlopidine (after) | fosfomycin (after) | trientine (after) | nitrofurantoin (after) |
| 660 | clopidogrel (after) | A16AX96 (after) | cilostazol (after) | A16AX95 (after) |
| 661 | tinzaparin (after) | selexipag (after) | prasugrel (after) | ticagrelor (after) |
| 662 | nadroparin (after) | ticagrelor (after) | treprostinil (after) | cilostazol (after) |
| 663 | enoxaparin (after) | cilostazol (after) | tirofiban (after) | prasugrel (after) |
| 664 | heparin (after) | prasugrel (after) | iloprost (after) | treprostinil (after) |
| 665 | warfarin (after) | treprostinil (after) | dipyridamole (after) | tirofiban (after) |
| 666 | A16AX96 (after) | tirofiban (after) | acetylsalicylic acid (after) | iloprost (after) |
| 667 | A16AX95 (after) | iloprost (after) | ticlopidine (after) | dipyridamole (after) |
| 668 | trientine (after) | dipyridamole (after) | clopidogrel (after) | acetylsalicylic acid (after) |
| 669 | miglustat (after) | acetylsalicylic acid (after) | tinzaparin (after) | ticlopidine (after) |
| 670 | zinc acetate (after) | ticlopidine (after) | nadroparin (after) | clopidogrel (after) |
| 671 | sodium phenylbutyrate (after) | clopidogrel (after) | enoxaparin (after) | tinzaparin (after) |
| 672 | iloprost (after) | tinzaparin (after) | heparin (after) | nadroparin (after) |
| 673 | treprostinil (after) | nadroparin (after) | warfarin (after) | enoxaparin (after) |
| 674 | vildagliptin (after) | enoxaparin (after) | A16AX96 (after) | heparin (after) |
| 675 | prasugrel (after) | heparin (after) | ticagrelor (after) | warfarin (after) |
| 676 | aprotinin (after) | combinations (after) | selexipag (after) | selexipag (after) |
| 677 | tranexamic acid (after) | streptokinase (after) | combinations (after) | combinations (after) |
| 678 | fondaparinux (after) | alteplase (after) | fondaparinux (after) | streptokinase (after) |
| 679 | edoxaban (after) | aprotinin (after) | coagulation factor VIII (after) | tranexamic acid (after) |
| 680 | apixaban (after) | coagulation factor IX (after) | Blood coagulation factors (after) | factor VIII inhibitor bypassing activity (after) |
| 681 | rivaroxaban (after) | factor VIII inhibitor bypassing activity (after) | phytomenadione (after) | coagulation factor VIII (after) |
| 682 | dabigatran etexilate (after) | coagulation factor VIII (after) | B02AB91 (after) | Blood coagulation factors (after) |
| 683 | drotrecogin alfa (activated) (after) | Blood coagulation factors (after) | aprotinin (after) | phytomenadione (after) |
| 684 | urokinase (after) | phytomenadione (after) | tranexamic acid (after) | B02AB91 (after) |
| 685 | alteplase (after) | B02AB91 (after) | edoxaban (after) | aprotinin (after) |
| 686 | streptokinase (after) | tranexamic acid (after) | streptokinase (after) | fondaparinux (after) |
| 687 | combinations (after) | urokinase (after) | apixaban (after) | alteplase (after) |
| 688 | selexipag (after) | fondaparinux (after) | rivaroxaban (after) | edoxaban (after) |
| 689 | ticagrelor (after) | edoxaban (after) | dabigatran etexilate (after) | apixaban (after) |
| 690 | cilostazol (after) | apixaban (after) | drotrecogin alfa (activated) (after) | rivaroxaban (after) |
| 691 | agalsidase beta (after) | rivaroxaban (after) | urokinase (after) | dabigatran etexilate (after) |
| 692 | agalsidase alfa (after) | dabigatran etexilate (after) | alteplase (after) | drotrecogin alfa (activated) (after) |
| 693 | imiglucerase (after) | drotrecogin alfa (activated) (after) | A16AX95 (after) | urokinase (after) |
| 694 | levocarnitine (after) | warfarin (after) | miglustat (after) | A16AX96 (after) |
| 695 | calcitriol (after) | A16AX95 (after) | coagulation factor IX (after) | trientine (after) |
| 696 | multivitamins and other minerals, incl. combinations (after) | romiplostim (after) | zinc acetate (after) | coagulation factor VIIa (after) |
| 697 | multivitamins and iron (after) | trientine (after) | pyridoxine (vit B6) (after) | miglustat (after) |
| 698 | mitiglinide (after) | pyridoxal phosphate (after) | ascorbic acid (vit C) (after) | tocopherol (vit E) (after) |
| 699 | nateglinide (after) | tocopherol (vit E) (after) | Vitamin B-complex, other combinations (after) | pyridoxine (vit B6) (after) |
| 700 | repaglinide (after) | pyridoxine (vit B6) (after) | Vitamin B1 in combination with vitamin B6 and/or vitamin B12 (after) | ascorbic acid (vit C) (after) |
| 701 | guar gum (after) | ascorbic acid (vit C) (after) | thiamine (vit B1) (after) | Vitamin B-complex, other combinations (after) |
| 702 | empagliflozin (after) | Vitamin B-complex, other combinations (after) | calcitriol (after) | Vitamin B1 in combination with vitamin B6 and/or vitamin B12 (after) |
| 703 | canagliflozin (after) | Vitamin B1 in combination with vitamin B6 and/or vitamin B12 (after) | multivitamins and other minerals, incl. combinations (after) | thiamine (vit B1) (after) |
| 704 | dapagliflozin (after) | thiamine (vit B1) (after) | multivitamins and iron (after) | calcitriol (after) |
| 705 | dulaglutide (after) | calcitriol (after) | mitiglinide (after) | multivitamins and other minerals, incl. combinations (after) |
| 706 | liraglutide (after) | multivitamins and other minerals, incl. combinations (after) | nateglinide (after) | multivitamins and iron (after) |
| 707 | exenatide (after) | multivitamins and iron (after) | repaglinide (after) | mitiglinide (after) |
| 708 | linagliptin (after) | mitiglinide (after) | guar gum (after) | nateglinide (after) |
| 709 | alogliptin (after) | nateglinide (after) | empagliflozin (after) | repaglinide (after) |
| 710 | thiamine (vit B1) (after) | repaglinide (after) | canagliflozin (after) | guar gum (after) |
| 711 | Vitamin B1 in combination with vitamin B6 and/or vitamin B12 (after) | guar gum (after) | dapagliflozin (after) | empagliflozin (after) |
| 712 | Vitamin B-complex, other combinations (after) | empagliflozin (after) | tocopherol (vit E) (after) | canagliflozin (after) |
| 713 | Calcium, combinations with vitamin D and/or other drugs (after) | calcium gluconate (after) | pyridoxal phosphate (after) | pyridoxal phosphate (after) |
| 714 | nandrolone (after) | calcium carbonate (after) | calcium gluconate (after) | calcium gluconate (after) |
| 715 | fluoride, combinations (after) | calcium (different salts in combination) (after) | fluoride, combinations (after) | calcium carbonate (after) |
| 716 | zinc gluconate (after) | levocarnitine (after) | sodium phenylbutyrate (after) | nandrolone (after) |
| 717 | potassium chloride, combinations (after) | miglustat (after) | agalsidase beta (after) | zinc acetate (after) |
| 718 | potassium gluconate (after) | zinc acetate (after) | agalsidase alfa (after) | sodium phenylbutyrate (after) |
| 719 | potassium chloride (after) | sodium phenylbutyrate (after) | imiglucerase (after) | agalsidase beta (after) |
| 720 | A12AA91 (after) | agalsidase beta (after) | levocarnitine (after) | agalsidase alfa (after) |
| 721 | ascorbic acid (vit C) (after) | agalsidase alfa (after) | nandrolone (after) | imiglucerase (after) |
| 722 | calcium (different salts in combination) (after) | imiglucerase (after) | zinc gluconate (after) | levocarnitine (after) |
| 723 | calcium carbonate (after) | nandrolone (after) | calcium carbonate (after) | fluoride, combinations (after) |
| 724 | calcium gluconate (after) | A12AA91 (after) | potassium chloride, combinations (after) | calcium (different salts in combination) (after) |
| 725 | pyridoxal phosphate (after) | fluoride, combinations (after) | potassium gluconate (after) | zinc gluconate (after) |
| 726 | tocopherol (vit E) (after) | zinc gluconate (after) | potassium chloride (after) | potassium chloride, combinations (after) |
| 727 | pyridoxine (vit B6) (after) | potassium chloride, combinations (after) | Calcium, combinations with vitamin D and/or other drugs (after) | potassium gluconate (after) |
| 728 | B02AB91 (after) | potassium gluconate (after) | A12AA91 (after) | potassium chloride (after) |
| 729 | phytomenadione (after) | potassium chloride (after) | calcium (different salts in combination) (after) | Calcium, combinations with vitamin D and/or other drugs (after) |
| 730 | Blood coagulation factors (after) | Calcium, combinations with vitamin D and/or other drugs (after) | factor VIII inhibitor bypassing activity (after) | A12AA91 (after) |
| 731 | sodium bicarbonate (after) | coagulation factor VIIa (after) | coagulation factor VIIa (after) | coagulation factor IX (after) |
| 732 | flecainide (after) | eltrombopag (after) | liraglutide (after) | romiplostim (after) |
| 733 | propafenone (after) | dapagliflozin (after) | calcium chloride (after) | dulaglutide (after) |
| 734 | mexiletine (after) | Vitamins (after) | isoprenaline (after) | electrolytes in combination with other drugs (after) |
| 735 | lidocaine (after) | dopamine (after) | etilefrine (after) | norepinephrine (after) |
| 736 | disopyramide (after) | norepinephrine (after) | dronedarone (after) | isoprenaline (after) |
| 737 | procainamide (after) | isoprenaline (after) | amiodarone (after) | etilefrine (after) |
| 738 | quinidine (after) | etilefrine (after) | flecainide (after) | dronedarone (after) |
| 739 | digoxin (after) | dronedarone (after) | propafenone (after) | amiodarone (after) |
| 740 | hemin (after) | amiodarone (after) | mexiletine (after) | flecainide (after) |
| 741 | Hemodialytics, concentrates (after) | flecainide (after) | lidocaine (after) | propafenone (after) |
| 742 | Vitamins (after) | propafenone (after) | disopyramide (after) | mexiletine (after) |
| 743 | electrolytes in combination with other drugs (after) | mexiletine (after) | procainamide (after) | lidocaine (after) |
| 744 | calcium chloride (after) | lidocaine (after) | quinidine (after) | disopyramide (after) |
| 745 | potassium phosphate, incl. combinations with other potassium salts (after) | disopyramide (after) | digoxin (after) | procainamide (after) |
| 746 | magnesium sulfate (after) | procainamide (after) | hemin (after) | quinidine (after) |
| 747 | amiodarone (after) | quinidine (after) | Hemodialytics, concentrates (after) | digoxin (after) |
| 748 | dronedarone (after) | digoxin (after) | Vitamins (after) | hemin (after) |
| 749 | etilefrine (after) | hemin (after) | norepinephrine (after) | Hemodialytics, concentrates (after) |
| 750 | glyceryl trinitrate (after) | phenylephrine (after) | dopamine (after) | dopamine (after) |
| 751 | adenosine (after) | dobutamine (after) | phenylephrine (after) | phenylephrine (after) |
| 752 | ubidecarenone (after) | midodrine (after) | alprostadil (after) | dobutamine (after) |
| 753 | alprostadil (after) | adenosine (after) | prazosin (after) | ubidecarenone (after) |
| 754 | nicorandil (after) | hydralazine (after) | clonidine (after) | doxazosin (after) |
| 755 | isosorbide mononitrate (after) | doxazosin (after) | methyldopa (racemic) (after) | prazosin (after) |
| 756 | isosorbide dinitrate (after) | prazosin (after) | ivabradine (after) | clonidine (after) |
| 757 | milrinone (after) | clonidine (after) | adenosine (after) | methyldopa (racemic) (after) |
| 758 | isoprenaline (after) | methyldopa (racemic) (after) | ubidecarenone (after) | ivabradine (after) |
| 759 | epinephrine (after) | ivabradine (after) | nicorandil (after) | adenosine (after) |
| 760 | midodrine (after) | ubidecarenone (after) | dobutamine (after) | alprostadil (after) |
| 761 | dobutamine (after) | epinephrine (after) | isosorbide mononitrate (after) | midodrine (after) |
| 762 | phenylephrine (after) | alprostadil (after) | isosorbide dinitrate (after) | nicorandil (after) |
| 763 | dopamine (after) | nicorandil (after) | glyceryl trinitrate (after) | isosorbide mononitrate (after) |
| 764 | norepinephrine (after) | isosorbide mononitrate (after) | milrinone (after) | isosorbide dinitrate (after) |
| 765 | sodium chloride (after) | isosorbide dinitrate (after) | epinephrine (after) | glyceryl trinitrate (after) |
| 766 | potassium chloride (after) | glyceryl trinitrate (after) | midodrine (after) | milrinone (after) |
| 767 | coagulation factor VIII (after) | milrinone (after) | electrolytes in combination with other drugs (after) | epinephrine (after) |
| 768 | Hypertonic solutions (after) | Hemodialytics, concentrates (after) | potassium phosphate, incl. combinations with other potassium salts (after) | Vitamins (after) |
| 769 | folic acid (after) | electrolytes in combination with other drugs (after) | romiplostim (after) | calcium chloride (after) |
| 770 | mecobalamin (after) | saccharated iron oxide (after) | magnesium sulfate (after) | eltrombopag (after) |
| 771 | cobamamide (after) | calcium chloride (after) | albumin (after) | potassium phosphate, incl. combinations with other potassium salts (after) |
| 772 | hydroxocobalamin (after) | gelatin agents (after) | methoxy polyethylene glycol-epoetin beta (after) | dextran (after) |
| 773 | various combinations (after) | dextran (after) | darbepoetin alfa (after) | albumin (after) |
| 774 | iron and multivitamins (after) | albumin (after) | erythropoietin (after) | methoxy polyethylene glycol-epoetin beta (after) |
| 775 | iron, multivitamins and folic acid (after) | methoxy polyethylene glycol-epoetin beta (after) | folic acid (after) | darbepoetin alfa (after) |
| 776 | Iron, parenteral preparations (after) | darbepoetin alfa (after) | mecobalamin (after) | erythropoietin (after) |
| 777 | ferric hydroxide (after) | erythropoietin (after) | cobamamide (after) | folic acid (after) |
| 778 | saccharated iron oxide (after) | folic acid (after) | hydroxocobalamin (after) | mecobalamin (after) |
| 779 | eltrombopag (after) | mecobalamin (after) | various combinations (after) | cobamamide (after) |
| 780 | romiplostim (after) | cobamamide (after) | iron and multivitamins (after) | hydroxocobalamin (after) |
| 781 | coagulation factor VIIa (after) | hydroxocobalamin (after) | iron, multivitamins and folic acid (after) | various combinations (after) |
| 782 | coagulation factor IX (after) | various combinations (after) | Iron, parenteral preparations (after) | iron and multivitamins (after) |
| 783 | factor VIII inhibitor bypassing activity (after) | iron and multivitamins (after) | ferric hydroxide (after) | iron, multivitamins and folic acid (after) |
| 784 | erythropoietin (after) | iron, multivitamins and folic acid (after) | saccharated iron oxide (after) | Iron, parenteral preparations (after) |
| 785 | darbepoetin alfa (after) | Iron, parenteral preparations (after) | eltrombopag (after) | ferric hydroxide (after) |
| 786 | methoxy polyethylene glycol-epoetin beta (after) | ferric hydroxide (after) | dextran (after) | saccharated iron oxide (after) |
| 787 | electrolytes (after) | hydroxyethylstarch (after) | gelatin agents (after) | gelatin agents (after) |
| 788 | combinations (after) | amino acids (after) | hydroxyethylstarch (after) | hydroxyethylstarch (after) |
| 789 | sodium chloride (after) | fat emulsions (after) | B05BC92 (after) | amino acids (after) |
| 790 | B05BC92 (after) | combinations (after) | sodium chloride (after) | sodium chloride (after) |
| 791 | mannitol (after) | potassium phosphate, incl. combinations with other potassium salts (after) | sodium bicarbonate (after) | magnesium sulfate (after) |
| 792 | Solutions producing osmotic diuresis (after) | magnesium sulfate (after) | potassium chloride (after) | sodium chloride (after) |
| 793 | electrolytes with carbohydrates (after) | sodium chloride (after) | Hypertonic solutions (after) | sodium bicarbonate (after) |
| 794 | combinations (after) | sodium bicarbonate (after) | combinations (after) | potassium chloride (after) |
| 795 | albumin (after) | potassium chloride (after) | sodium chloride (after) | Hypertonic solutions (after) |
| 796 | carbohydrates (after) | Hypertonic solutions (after) | mannitol (after) | combinations (after) |
| 797 | fat emulsions (after) | sodium chloride (after) | amino acids (after) | B05BC92 (after) |
| 798 | amino acids (after) | carbohydrates (after) | Solutions producing osmotic diuresis (after) | fat emulsions (after) |
| 799 | hydroxyethylstarch (after) | B05BC92 (after) | electrolytes with carbohydrates (after) | mannitol (after) |
| 800 | gelatin agents (after) | mannitol (after) | electrolytes (after) | Solutions producing osmotic diuresis (after) |
| 801 | dextran (after) | Solutions producing osmotic diuresis (after) | combinations (after) | electrolytes with carbohydrates (after) |
| 802 | saxagliptin (after) | electrolytes with carbohydrates (after) | carbohydrates (after) | electrolytes (after) |
| 803 | sitagliptin (after) | electrolytes (after) | fat emulsions (after) | combinations (after) |
| 804 | teicoplanin (after) | combinations (after) | dulaglutide (after) | carbohydrates (after) |
| 805 | pantoprazole (after) | canagliflozin (after) | exenatide (after) | dapagliflozin (after) |
| 806 | misoprostol (after) | dulaglutide (after) | hydralazine (after) | liraglutide (after) |
| 807 | famotidine (after) | nitrofurantoin (after) | aluminium hydroxide (after) | metronidazole (after) |
| 808 | ranitidine (after) | hydrotalcite (after) | dexlansoprazole (after) | ordinary salt combinations (after) |
| 809 | cimetidine (after) | alginic acid (after) | esomeprazole (after) | sucralfate (after) |
| 810 | Antacids, other combinations (after) | sucralfate (after) | rabeprazole (after) | dexlansoprazole (after) |
| 811 | Antacids with sodium bicarbonate (after) | dexlansoprazole (after) | lansoprazole (after) | esomeprazole (after) |
| 812 | Antacids with antispasmodics (after) | esomeprazole (after) | pantoprazole (after) | rabeprazole (after) |
| 813 | ordinary salt combinations and antiflatulents (after) | rabeprazole (after) | omeprazole (after) | lansoprazole (after) |
| 814 | hydrotalcite (after) | lansoprazole (after) | misoprostol (after) | pantoprazole (after) |
| 815 | ordinary salt combinations (after) | pantoprazole (after) | famotidine (after) | omeprazole (after) |
| 816 | aluminium hydroxide (after) | omeprazole (after) | ranitidine (after) | misoprostol (after) |
| 817 | Aluminium compounds (after) | misoprostol (after) | cimetidine (after) | famotidine (after) |
| 818 | magnesium oxide (after) | famotidine (after) | Antacids, other combinations (after) | ranitidine (after) |
| 819 | epinephrine (after) | ranitidine (after) | Antacids with sodium bicarbonate (after) | cimetidine (after) |
| 820 | A01AC91 (after) | cimetidine (after) | Antacids with antispasmodics (after) | Antacids, other combinations (after) |
| 821 | omeprazole (after) | Antacids, other combinations (after) | ordinary salt combinations and antiflatulents (after) | Antacids with sodium bicarbonate (after) |
| 822 | lansoprazole (after) | Antacids with sodium bicarbonate (after) | hydrotalcite (after) | Antacids with antispasmodics (after) |
| 823 | pioglitazone (after) | Antacids with antispasmodics (after) | sucralfate (after) | ordinary salt combinations and antiflatulents (after) |
| 824 | rabeprazole (after) | mebeverine (after) | alginic acid (after) | alginic acid (after) |
| 825 | butylscopolamine (after) | glycopyrronium bromide (after) | mebeverine (after) | mebeverine (after) |
| 826 | atropine (after) | propantheline (after) | atropine (after) | glycopyrronium bromide (after) |
| 827 | A03AX95 (after) | metoclopramide (after) | ondansetron (after) | butylscopolamine (after) |
| 828 | alverine (after) | tropisetron (after) | mosapride (after) | granisetron (after) |
| 829 | pinaverium (after) | granisetron (after) | domperidone (after) | ondansetron (after) |
| 830 | papaverine (after) | ondansetron (after) | cisapride (after) | mosapride (after) |
| 831 | mepenzolate (after) | mosapride (after) | metoclopramide (after) | domperidone (after) |
| 832 | otilonium bromide (after) | domperidone (after) | butylscopolamine (after) | cisapride (after) |
| 833 | propantheline (after) | cisapride (after) | A03AX95 (after) | metoclopramide (after) |
| 834 | glycopyrronium bromide (after) | butylscopolamine (after) | glycopyrronium bromide (after) | atropine (after) |
| 835 | mebeverine (after) | otilonium bromide (after) | alverine (after) | propantheline (after) |
| 836 | alginic acid (after) | atropine (after) | pinaverium (after) | A03AX95 (after) |
| 837 | sucralfate (after) | A03AX95 (after) | papaverine (after) | alverine (after) |
| 838 | dexlansoprazole (after) | alverine (after) | mepenzolate (after) | pinaverium (after) |
| 839 | esomeprazole (after) | pinaverium (after) | otilonium bromide (after) | papaverine (after) |
| 840 | hydrocortisone (after) | papaverine (after) | propantheline (after) | mepenzolate (after) |
| 841 | dexamethasone (after) | mepenzolate (after) | ordinary salt combinations (after) | otilonium bromide (after) |
| 842 | triamcinolone (after) | ordinary salt combinations and antiflatulents (after) | Aluminium compounds (after) | hydrotalcite (after) |
| 843 | chlorhexidine (after) | ordinary salt combinations (after) | tropisetron (after) | aluminium hydroxide (after) |
| 844 | Neurocognitive disorders (after) | palonosetron, combinations (after) | magnesium oxide (after) | palonosetron (after) |
| 845 | Neurocognitive disorders (before) | aluminium hydroxide (after) | Dissociative disorders (before) | Aluminium compounds (after) |
| 846 | Substance-related and addictive disorders (before) | Feeding and eating disorders (before) | Trauma- and stressor-related disorders (before) | Dissociative disorders (after) |
| 847 | Sexual dysfunctions (after) | Dissociative disorders (after) | Personality disorders (after) | Dissociative disorders (before) |
| 848 | Elimination disorders (after) | Dissociative disorders (before) | Personality disorders (before) | Personality disorders (after) |
| 849 | Elimination disorders (before) | Trauma- and stressor-related disorders (after) | Neurocognitive disorders (after) | Personality disorders (before) |
| 850 | Somatic symptom and related disorders (after) | Personality disorders (before) | Neurocognitive disorders (before) | Neurocognitive disorders (after) |
| 851 | Somatic symptom and related disorders (before) | Neurocognitive disorders (after) | Sexual dysfunctions (before) | Neurocognitive disorders (before) |
| 852 | Obsessive-compulsive and related disorders (before) | Neurocognitive disorders (before) | Somatic symptom and related disorders (before) | Elimination disorders (before) |
| 853 | Bipolar and related disorders (after) | Substance-related and addictive disorders (before) | Obsessive-compulsive and related disorders (before) | Somatic symptom and related disorders (before) |
| 854 | Bipolar and related disorders (before) | Elimination disorders (after) | Bipolar and related disorders (after) | Obsessive-compulsive and related disorders (before) |
| 855 | Disruptive, impulse-control, and conduct disorders (after) | Bipolar and related disorders (after) | Bipolar and related disorders (before) | Bipolar and related disorders (after) |
| 856 | Disruptive, impulse-control, and conduct disorders (before) | Bipolar and related disorders (before) | Disruptive, impulse-control, and conduct disorders (after) | Bipolar and related disorders (before) |
| 857 | Neurodevelopmental disorders (after) | Disruptive, impulse-control, and conduct disorders (after) | Disruptive, impulse-control, and conduct disorders (before) | Schizophrenia spectrum and other psychotic disorders (before) |
| 858 | Neurodevelopmental disorders (before) | Disruptive, impulse-control, and conduct disorders (before) | Neurodevelopmental disorders (after) | Disruptive, impulse-control, and conduct disorders (after) |
| 859 | Personality disorders (before) | Neurodevelopmental disorders (after) | Neurodevelopmental disorders (before) | Disruptive, impulse-control, and conduct disorders (before) |
| 860 | Trauma- and stressor-related disorders (before) | Neurodevelopmental disorders (before) | Dissociative disorders (after) | Neurodevelopmental disorders (before) |
| 861 | Trauma- and stressor-related disorders (after) | Feeding and eating disorders (after) | Feeding and eating disorders (before) | Feeding and eating disorders (before) |
| 862 | Rheumatic disease (after) | Gender dysporia (before) | Feeding and eating disorders (after) | Feeding and eating disorders (after) |
| 863 | Tumor (after) | Gender dysporia (after) | Heart (after) | Gender dysporia (before) |
| 864 | HIV (after) | triamcinolone (after) | epinephrine (after) | chlorhexidine (after) |
| 865 | HIV (before) | Aluminium compounds (after) | A01AC91 (after) | magnesium oxide (after) |
| 866 | Renal disease (after) | magnesium oxide (after) | hydrocortisone (after) | epinephrine (after) |
| 867 | Ulcer disease (after) | epinephrine (after) | dexamethasone (after) | A01AC91 (after) |
| 868 | Ulcer disease (before) | A01AC91 (after) | triamcinolone (after) | hydrocortisone (after) |
| 869 | Parahilic disorders (after) | hydrocortisone (after) | chlorhexidine (after) | dexamethasone (after) |
| 870 | Dissociative disorders (before) | dexamethasone (after) | HIV (after) | triamcinolone (after) |
| 871 | Parahilic disorders (before) | chlorhexidine (after) | Gender dysporia (before) | HIV (after) |
| 872 | Gender dysporia (after) | Parahilic disorders (before) | HIV (before) | Gender dysporia (after) |
| 873 | Gender dysporia (before) | HIV (after) | Ulcer disease (after) | HIV (before) |
| 874 | Feeding and eating disorders (after) | HIV (before) | Ulcer disease (before) | Ulcer disease (after) |
| 875 | Feeding and eating disorders (before) | Ulcer disease (after) | Parahilic disorders (after) | Ulcer disease (before) |
| 876 | Dissociative disorders (after) | Ulcer disease (before) | Parahilic disorders (before) | Rheumatic disease (after) |
| 877 | metoclopramide (after) | Rheumatic disease (after) | Gender dysporia (after) | Rheumatic disease (before) |
| 878 | cisapride (after) | Parahilic disorders (after) | granisetron (after) | Parahilic disorders (after) |
| 879 | domperidone (after) | palonosetron (after) | palonosetron (after) | tropisetron (after) |
| 880 | multienzymes (lipase, protease etc.) (after) | aprepitant (after) | linagliptin (after) | palonosetron, combinations (after) |
| 881 | metformin (after) | liraglutide (after) | insulin aspart (after) | exenatide (after) |
| 882 | insulin glargine and lixisenatide (after) | insulin (human) (after) | metformin and sulfonylureas (after) | insulin glulisine (after) |
| 883 | insulin degludec (after) | metformin and pioglitazone (after) | glimepiride (after) | metformin and rosiglitazone (after) |
| 884 | insulin detemir (after) | metformin and rosiglitazone (after) | gliclazide (after) | metformin and sulfonylureas (after) |
| 885 | insulin glargine (after) | metformin and sulfonylureas (after) | glipizide (after) | glimepiride (after) |
| 886 | insulin (human) (after) | glimepiride (after) | chlorpropamide (after) | gliclazide (after) |
| 887 | insulin aspart (after) | gliclazide (after) | metformin (after) | glipizide (after) |
| 888 | insulin lispro (after) | glipizide (after) | insulin glargine and lixisenatide (after) | chlorpropamide (after) |
| 889 | insulin (human) (after) | chlorpropamide (after) | insulin degludec (after) | metformin (after) |
| 890 | insulin (human) (after) | metformin (after) | insulin detemir (after) | insulin glargine and lixisenatide (after) |
| 891 | insulin glulisine (after) | insulin glargine and lixisenatide (after) | insulin glargine (after) | insulin degludec (after) |
| 892 | insulin aspart (after) | insulin degludec (after) | insulin (human) (after) | insulin detemir (after) |
| 893 | insulin lispro (after) | insulin detemir (after) | insulin aspart (after) | insulin glargine (after) |
| 894 | insulin (human) (after) | insulin glargine (after) | insulin lispro (after) | insulin (human) (after) |
| 895 | multienzymes and acid preparations (after) | insulin (human) (after) | insulin (human) (after) | insulin aspart (after) |
| 896 | chlorpropamide (after) | insulin aspart (after) | insulin (human) (after) | insulin lispro (after) |
| 897 | glipizide (after) | insulin lispro (after) | metformin and rosiglitazone (after) | insulin (human) (after) |
| 898 | gliclazide (after) | metformin and sitagliptin (after) | metformin and pioglitazone (after) | metformin and pioglitazone (after) |
| 899 | metformin and linagliptin (after) | metformin and vildagliptin (after) | metformin and sitagliptin (after) | metformin and sitagliptin (after) |
| 900 | rosiglitazone (after) | pioglitazone and alogliptin (after) | acarbose (after) | metformin and vildagliptin (after) |
| 901 | acarbose (after) | pioglitazone (after) | alogliptin (after) | rosiglitazone (after) |
| 902 | saxagliptin and dapagliflozin (after) | exenatide (after) | saxagliptin (after) | linagliptin (after) |
| 903 | metformin and empagliflozin (after) | linagliptin (after) | vildagliptin (after) | alogliptin (after) |
| 904 | linagliptin and empagliflozin (after) | alogliptin (after) | sitagliptin (after) | saxagliptin (after) |
| 905 | metformin and dapagliflozin (after) | saxagliptin (after) | pioglitazone (after) | vildagliptin (after) |
| 906 | metformin and saxagliptin (after) | vildagliptin (after) | rosiglitazone (after) | sitagliptin (after) |
| 907 | glimepiride (after) | sitagliptin (after) | saxagliptin and dapagliflozin (after) | pioglitazone (after) |
| 908 | pioglitazone and alogliptin (after) | rosiglitazone (after) | metformin and vildagliptin (after) | acarbose (after) |
| 909 | metformin and vildagliptin (after) | metformin and saxagliptin (after) | metformin and empagliflozin (after) | pioglitazone and alogliptin (after) |
| 910 | metformin and sitagliptin (after) | acarbose (after) | linagliptin and empagliflozin (after) | saxagliptin and dapagliflozin (after) |
| 911 | metformin and pioglitazone (after) | saxagliptin and dapagliflozin (after) | metformin and dapagliflozin (after) | metformin and empagliflozin (after) |
| 912 | metformin and rosiglitazone (after) | metformin and empagliflozin (after) | metformin and linagliptin (after) | linagliptin and empagliflozin (after) |
| 913 | metformin and sulfonylureas (after) | linagliptin and empagliflozin (after) | metformin and saxagliptin (after) | metformin and dapagliflozin (after) |
| 914 | tilactase (after) | metformin and dapagliflozin (after) | pioglitazone and alogliptin (after) | metformin and linagliptin (after) |
| 915 | racecadotril (after) | metformin and linagliptin (after) | insulin glulisine (after) | metformin and saxagliptin (after) |
| 916 | mosapride (after) | insulin (human) (after) | insulin lispro (after) | insulin (human) (after) |
| 917 | lactic acid producing organisms, combinations (after) | insulin glulisine (after) | palonosetron, combinations (after) | insulin aspart (after) |
| 918 | ispaghula (psylla seeds) (after) | chenodeoxycholic acid (after) | insulin (human) (after) | aprepitant (after) |
| 919 | contact laxatives in combination (after) | insulin aspart (after) | lactitol (after) | insulin lispro (after) |
| 920 | senna glycosides (after) | sodium phosphate (after) | lactulose (after) | macrogol (after) |
| 921 | bisacodyl (after) | macrogol (after) | mineral salts in combination (after) | lactitol (after) |
| 922 | silymarin (after) | lactitol (after) | sterculia, combinations (after) | lactulose (after) |
| 923 | Liver therapy (after) | lactulose (after) | polycarbophil calcium (after) | mineral salts in combination (after) |
| 924 | Other drugs for bile therapy (after) | mineral salts in combination (after) | ispaghula (psylla seeds) (after) | sterculia, combinations (after) |
| 925 | ursodeoxycholic acid (after) | sterculia, combinations (after) | contact laxatives in combination (after) | polycarbophil calcium (after) |
| 926 | chenodeoxycholic acid (after) | polycarbophil calcium (after) | senna glycosides (after) | ispaghula (psylla seeds) (after) |
| 927 | aprepitant (after) | ispaghula (psylla seeds) (after) | bisacodyl (after) | contact laxatives in combination (after) |
| 928 | palonosetron, combinations (after) | contact laxatives in combination (after) | silymarin (after) | senna glycosides (after) |
| 929 | palonosetron (after) | senna glycosides (after) | Liver therapy (after) | bisacodyl (after) |
| 930 | tropisetron (after) | bisacodyl (after) | Other drugs for bile therapy (after) | silymarin (after) |
| 931 | granisetron (after) | silymarin (after) | ursodeoxycholic acid (after) | Liver therapy (after) |
| 932 | ondansetron (after) | Liver therapy (after) | chenodeoxycholic acid (after) | Other drugs for bile therapy (after) |
| 933 | polycarbophil calcium (after) | Other drugs for bile therapy (after) | aprepitant (after) | ursodeoxycholic acid (after) |
| 934 | sterculia, combinations (after) | ursodeoxycholic acid (after) | macrogol (after) | chenodeoxycholic acid (after) |
| 935 | mineral salts in combination (after) | nystatin (after) | sodium phosphate (after) | sodium phosphate (after) |
| 936 | diosmectite (after) | vancomycin (after) | nystatin (after) | nystatin (after) |
| 937 | mesalazine (after) | fidaxomicin (after) | sulfasalazine (after) | vancomycin (after) |
| 938 | sulfasalazine (after) | lactic acid producing organisms, combinations (after) | multienzymes and acid preparations (after) | mesalazine (after) |
| 939 | budesonide (after) | insulin lispro (after) | tilactase (after) | insulin (human) (after) |
| 940 | loperamide (after) | insulin (human) (after) | multienzymes (lipase, protease etc.) (after) | multienzymes and acid preparations (after) |
| 941 | Oral rehydration salt formulations (after) | multienzymes and acid preparations (after) | racecadotril (after) | tilactase (after) |
| 942 | combinations (after) | tilactase (after) | lactic acid producing organisms, combinations (after) | multienzymes (lipase, protease etc.) (after) |
| 943 | medicinal charcoal (after) | multienzymes (lipase, protease etc.) (after) | mesalazine (after) | racecadotril (after) |
| 944 | lactulose (after) | racecadotril (after) | budesonide (after) | lactic acid producing organisms, combinations (after) |
| 945 | fidaxomicin (after) | mesalazine (after) | vancomycin (after) | sulfasalazine (after) |
| 946 | vancomycin (after) | medicinal charcoal (after) | loperamide (after) | fidaxomicin (after) |
| 947 | nystatin (after) | sulfasalazine (after) | Oral rehydration salt formulations (after) | budesonide (after) |
| 948 | sodium phosphate (after) | budesonide (after) | combinations (after) | loperamide (after) |
| 949 | macrogol (after) | loperamide (after) | diosmectite (after) | Oral rehydration salt formulations (after) |
| 950 | lactitol (after) | Oral rehydration salt formulations (after) | medicinal charcoal (after) | combinations (after) |
| 951 | ivabradine (after) | combinations (after) | fidaxomicin (after) | diosmectite (after) |
| 952 | methyldopa (racemic) (after) | diosmectite (after) | doxazosin (after) | medicinal charcoal (after) |
| 953 | clonidine (after) | minoxidil (after) | minoxidil (after) | hydralazine (after) |
| 954 | tamsulosin (after) | nitroprusside (after) | metronidazole (after) | minoxidil (after) |
| 955 | pentosan polysulfate sodium (after) | bosentan (after) | raloxifene (after) | nitroprusside (after) |
| 956 | phenazopyridine (after) | bazedoxifene (after) | silodosin (after) | raloxifene (after) |
| 957 | sildenafil (after) | tamsulosin and dutasteride (after) | terazosin (after) | silodosin (after) |
| 958 | alprostadil (after) | silodosin (after) | tamsulosin (after) | terazosin (after) |
| 959 | mirabegron (after) | terazosin (after) | alfuzosin (after) | tamsulosin (after) |
| 960 | solifenacin (after) | tamsulosin (after) | pentosan polysulfate sodium (after) | alfuzosin (after) |
| 961 | tolterodine (after) | alfuzosin (after) | phenazopyridine (after) | pentosan polysulfate sodium (after) |
| 962 | propiverine (after) | pentosan polysulfate sodium (after) | sildenafil (after) | phenazopyridine (after) |
| 963 | oxybutynin (after) | phenazopyridine (after) | alprostadil (after) | sildenafil (after) |
| 964 | flavoxate (after) | sildenafil (after) | mirabegron (after) | alprostadil (after) |
| 965 | Urinary concrement solvents (after) | alprostadil (after) | solifenacin (after) | mirabegron (after) |
| 966 | bazedoxifene (after) | mirabegron (after) | tolterodine (after) | solifenacin (after) |
| 967 | raloxifene (after) | solifenacin (after) | propiverine (after) | tolterodine (after) |
| 968 | gestrinone (after) | tolterodine (after) | oxybutynin (after) | propiverine (after) |
| 969 | danazol (after) | propiverine (after) | flavoxate (after) | oxybutynin (after) |
| 970 | alfuzosin (after) | oxybutynin (after) | Urinary concrement solvents (after) | flavoxate (after) |
| 971 | terazosin (after) | flavoxate (after) | tamsulosin and dutasteride (after) | Urinary concrement solvents (after) |
| 972 | prazosin (after) | finasteride (after) | finasteride (after) | tamsulosin and dutasteride (after) |
| 973 | silodosin (after) | dutasteride (after) | dutasteride (after) | finasteride (after) |
| 974 | fludrocortisone (after) | tetracosactide (after) | somatostatin (after) | dutasteride (after) |
| 975 | lanreotide (after) | octreotide (after) | methylprednisolone (after) | somatostatin (after) |
| 976 | octreotide (after) | prednisolone (after) | dexamethasone (after) | methylprednisolone (after) |
| 977 | somatostatin (after) | methylprednisolone (after) | Glucocorticoids (after) | dexamethasone (after) |
| 978 | gonadorelin (after) | dexamethasone (after) | fludrocortisone (after) | Glucocorticoids (after) |
| 979 | oxytocin (after) | Glucocorticoids (after) | lanreotide (after) | fludrocortisone (after) |
| 980 | terlipressin (after) | fludrocortisone (after) | octreotide (after) | lanreotide (after) |
| 981 | desmopressin (after) | lanreotide (after) | gonadorelin (after) | octreotide (after) |
| 982 | vasopressin (argipressin) (after) | somatostatin (after) | tetracosactide (after) | gonadorelin (after) |
| 983 | somatropin (after) | thyrotropin alfa (after) | oxytocin (after) | tetracosactide (after) |
| 984 | thyrotropin alfa (after) | gonadorelin (after) | terlipressin (after) | oxytocin (after) |
| 985 | tetracosactide (after) | oxytocin (after) | desmopressin (after) | terlipressin (after) |
| 986 | dutasteride (after) | terlipressin (after) | vasopressin (argipressin) (after) | desmopressin (after) |
| 987 | finasteride (after) | desmopressin (after) | somatropin (after) | vasopressin (argipressin) (after) |
| 988 | tamsulosin and dutasteride (after) | vasopressin (argipressin) (after) | thyrotropin alfa (after) | somatropin (after) |
| 989 | cyproterone and estrogen (after) | somatropin (after) | bazedoxifene (after) | thyrotropin alfa (after) |
| 990 | cyproterone (after) | Urinary concrement solvents (after) | gestrinone (after) | bazedoxifene (after) |
| 991 | clomifene (after) | raloxifene (after) | triamcinolone (after) | gestrinone (after) |
| 992 | chorionic gonadotrophin (after) | hydrocortisone (after) | danazol (after) | triamcinolone (after) |
| 993 | ritodrine (after) | gestrinone (after) | methyltestosterone (after) | danazol (after) |
| 994 | dinoprostone (after) | testosterone (after) | levonorgestrel (after) | methyltestosterone (after) |
| 995 | ergometrine (after) | methyltestosterone (after) | cabergoline (after) | levonorgestrel (after) |
| 996 | methylergometrine (after) | levonorgestrel (after) | bromocriptine (after) | cabergoline (after) |
| 997 | policresulen (after) | cabergoline (after) | ritodrine (after) | bromocriptine (after) |
| 998 | sertaconazole (after) | bromocriptine (after) | dinoprostone (after) | ritodrine (after) |
| 999 | fenticonazole (after) | ritodrine (after) | ergometrine (after) | dinoprostone (after) |
| 1000 | clotrimazole (after) | dinoprostone (after) | methylergometrine (after) | ergometrine (after) |
| 1001 | metronidazole (after) | ergometrine (after) | policresulen (after) | methylergometrine (after) |
| 1002 | nystatin (after) | methylergometrine (after) | sertaconazole (after) | policresulen (after) |
| 1003 | Antibiotics (after) | policresulen (after) | fenticonazole (after) | sertaconazole (after) |
| 1004 | dupilumab (after) | sertaconazole (after) | clotrimazole (after) | fenticonazole (after) |
| 1005 | tacrolimus (after) | fenticonazole (after) | metronidazole (after) | clotrimazole (after) |
| 1006 | Wart and anti-corn preparations (after) | clotrimazole (after) | nystatin (after) | metronidazole (after) |
| 1007 | isotretinoin (after) | metronidazole (after) | Antibiotics (after) | nystatin (after) |
| 1008 | bromocriptine (after) | nystatin (after) | testosterone (after) | Antibiotics (after) |
| 1009 | cabergoline (after) | estradiol (after) | estradiol (after) | testosterone (after) |
| 1010 | levonorgestrel (after) | estriol (after) | estriol (after) | estradiol (after) |
| 1011 | dienogest (after) | conjugated estrogens (after) | norethisterone and estrogen (after) | estriol (after) |
| 1012 | medroxyprogesterone and estrogen (after) | progesterone and estrogen (after) | cyproterone and estrogen (after) | norethisterone and estrogen (after) |
| 1013 | progesterone and estrogen (after) | danazol (after) | cyproterone (after) | cyproterone and estrogen (after) |
| 1014 | norethisterone and estrogen (after) | cyproterone and estrogen (after) | clomifene (after) | cyproterone (after) |
| 1015 | Androgen, progestogen and estrogen in combination (after) | cyproterone (after) | chorionic gonadotrophin (after) | clomifene (after) |
| 1016 | testosterone and estrogen (after) | clomifene (after) | medroxyprogesterone and estrogen (after) | chorionic gonadotrophin (after) |
| 1017 | norethisterone (after) | chorionic gonadotrophin (after) | progesterone and estrogen (after) | medroxyprogesterone and estrogen (after) |
| 1018 | dydrogesterone (after) | medroxyprogesterone and estrogen (after) | Androgen, progestogen and estrogen in combination (after) | progesterone and estrogen (after) |
| 1019 | methyltestosterone (after) | norethisterone and estrogen (after) | conjugated estrogens (after) | Androgen, progestogen and estrogen in combination (after) |
| 1020 | progesterone (after) | medroxyprogesterone (after) | testosterone and estrogen (after) | conjugated estrogens (after) |
| 1021 | medroxyprogesterone (after) | Androgen, progestogen and estrogen in combination (after) | norethisterone (after) | testosterone and estrogen (after) |
| 1022 | conjugated estrogens (after) | testosterone and estrogen (after) | dienogest (after) | norethisterone (after) |
| 1023 | estriol (after) | norethisterone (after) | dydrogesterone (after) | dienogest (after) |
| 1024 | estradiol (after) | dienogest (after) | progesterone (after) | dydrogesterone (after) |
| 1025 | testosterone (after) | dydrogesterone (after) | medroxyprogesterone (after) | progesterone (after) |
| 1026 | Glucocorticoids (after) | progesterone (after) | prednisolone (after) | medroxyprogesterone (after) |
| 1027 | dexamethasone (after) | triamcinolone (after) | hydrocortisone (after) | prednisolone (after) |
| 1028 | methylprednisolone (after) | cortisone (after) | tacrolimus (after) | hydrocortisone (after) |
| 1029 | cefaclor (after) | ambrisentan (after) | ceftazidime (after) | bosentan (after) |
| 1030 | ceftaroline fosamil (after) | ceftriaxone (after) | clarithromycin (after) | ceftazidime (after) |
| 1031 | imipenem and cilastatin (after) | azithromycin (after) | erythromycin (after) | clarithromycin (after) |
| 1032 | doripenem (after) | clarithromycin (after) | sulfamethoxazole and trimethoprim (after) | erythromycin (after) |
| 1033 | ertapenem (after) | erythromycin (after) | ceftaroline fosamil (after) | sulfamethoxazole and trimethoprim (after) |
| 1034 | meropenem (after) | sulfamethoxazole and trimethoprim (after) | imipenem and cilastatin (after) | ceftaroline fosamil (after) |
| 1035 | aztreonam (after) | ceftaroline fosamil (after) | doripenem (after) | imipenem and cilastatin (after) |
| 1036 | cefpirome (after) | imipenem and cilastatin (after) | ertapenem (after) | doripenem (after) |
| 1037 | cefepime (after) | doripenem (after) | meropenem (after) | ertapenem (after) |
| 1038 | cefoperazone and beta-lactamase inhibitor (after) | ertapenem (after) | aztreonam (after) | meropenem (after) |
| 1039 | ceftibuten (after) | meropenem (after) | cefpirome (after) | aztreonam (after) |
| 1040 | cefixime (after) | aztreonam (after) | cefepime (after) | cefpirome (after) |
| 1041 | ceftizoxime (after) | cefpirome (after) | cefoperazone and beta-lactamase inhibitor (after) | cefepime (after) |
| 1042 | ceftriaxone (after) | cefepime (after) | ceftibuten (after) | cefoperazone and beta-lactamase inhibitor (after) |
| 1043 | ceftazidime (after) | cefoperazone and beta-lactamase inhibitor (after) | cefixime (after) | ceftibuten (after) |
| 1044 | cefotaxime (after) | ceftibuten (after) | ceftizoxime (after) | cefixime (after) |
| 1045 | sulfamethoxazole and trimethoprim (after) | cefixime (after) | azithromycin (after) | ceftizoxime (after) |
| 1046 | erythromycin (after) | clindamycin (after) | clindamycin (after) | azithromycin (after) |
| 1047 | clarithromycin (after) | streptomycin (after) | streptomycin (after) | clindamycin (after) |
| 1048 | isepamicin (after) | gentamicin (after) | moxifloxacin (after) | streptomycin (after) |
| 1049 | vancomycin (after) | gemifloxacin (after) | fusidic acid (after) | moxifloxacin (after) |
| 1050 | nalidixic acid (after) | metronidazole (after) | colistin (after) | fusidic acid (after) |
| 1051 | gemifloxacin (after) | fusidic acid (after) | teicoplanin (after) | colistin (after) |
| 1052 | moxifloxacin (after) | colistin (after) | vancomycin (after) | teicoplanin (after) |
| 1053 | levofloxacin (after) | teicoplanin (after) | nalidixic acid (after) | vancomycin (after) |
| 1054 | ciprofloxacin (after) | vancomycin (after) | gemifloxacin (after) | nalidixic acid (after) |
| 1055 | netilmicin (after) | nalidixic acid (after) | levofloxacin (after) | gemifloxacin (after) |
| 1056 | azithromycin (after) | moxifloxacin (after) | gentamicin (after) | levofloxacin (after) |
| 1057 | amikacin (after) | kanamycin (after) | ciprofloxacin (after) | gentamicin (after) |
| 1058 | neomycin (after) | levofloxacin (after) | isepamicin (after) | ciprofloxacin (after) |
| 1059 | kanamycin (after) | ciprofloxacin (after) | netilmicin (after) | isepamicin (after) |
| 1060 | gentamicin (after) | isepamicin (after) | amikacin (after) | netilmicin (after) |
| 1061 | streptomycin (after) | netilmicin (after) | neomycin (after) | amikacin (after) |
| 1062 | clindamycin (after) | amikacin (after) | kanamycin (after) | neomycin (after) |
| 1063 | flomoxef (after) | neomycin (after) | ceftriaxone (after) | kanamycin (after) |
| 1064 | cefuroxime (after) | ceftizoxime (after) | cefotaxime (after) | ceftriaxone (after) |
| 1065 | prednisolone (after) | ceftazidime (after) | cortisone (after) | cefotaxime (after) |
| 1066 | cefoxitin (after) | levothyroxine sodium (after) | flomoxef (after) | cortisone (after) |
| 1067 | tigecycline (after) | cefotaxime (after) | amoxicillin (after) | flomoxef (after) |
| 1068 | minocycline (after) | piperacillin (after) | ampicillin (after) | amoxicillin (after) |
| 1069 | tetracycline (after) | amoxicillin (after) | chloramphenicol (after) | ampicillin (after) |
| 1070 | oxytetracycline (after) | ampicillin (after) | tigecycline (after) | chloramphenicol (after) |
| 1071 | doxycycline (after) | chloramphenicol (after) | minocycline (after) | tigecycline (after) |
| 1072 | calcitonin (salmon synthetic) (after) | tigecycline (after) | tetracycline (after) | minocycline (after) |
| 1073 | teriparatide (after) | minocycline (after) | oxytetracycline (after) | tetracycline (after) |
| 1074 | glucagon (after) | tetracycline (after) | doxycycline (after) | oxytetracycline (after) |
| 1075 | thiamazole (after) | oxytetracycline (after) | calcitonin (salmon synthetic) (after) | doxycycline (after) |
| 1076 | carbimazole (after) | doxycycline (after) | teriparatide (after) | calcitonin (salmon synthetic) (after) |
| 1077 | propylthiouracil (after) | calcitonin (salmon synthetic) (after) | glucagon (after) | teriparatide (after) |
| 1078 | levothyroxine sodium (after) | teriparatide (after) | thiamazole (after) | glucagon (after) |
| 1079 | cortisone (after) | glucagon (after) | carbimazole (after) | thiamazole (after) |
| 1080 | hydrocortisone (after) | thiamazole (after) | propylthiouracil (after) | carbimazole (after) |
| 1081 | triamcinolone (after) | carbimazole (after) | levothyroxine sodium (after) | propylthiouracil (after) |
| 1082 | chloramphenicol (after) | propylthiouracil (after) | piperacillin (after) | levothyroxine sodium (after) |
| 1083 | ampicillin (after) | benzylpenicillin (after) | benzylpenicillin (after) | piperacillin (after) |
| 1084 | amoxicillin (after) | phenoxymethylpenicillin (after) | phenoxymethylpenicillin (after) | benzylpenicillin (after) |
| 1085 | amoxicillin and beta-lactamase inhibitor (after) | benzathine benzylpenicillin (after) | cefalotin (after) | phenoxymethylpenicillin (after) |
| 1086 | cefradine (after) | cefazolin (after) | cefaclor (after) | cefalotin (after) |
| 1087 | cefadroxil (after) | flomoxef (after) | cefuroxime (after) | cefaclor (after) |
| 1088 | cefazolin (after) | cefaclor (after) | cefoxitin (after) | cefuroxime (after) |
| 1089 | cefalotin (after) | cefuroxime (after) | cefradine (after) | cefoxitin (after) |
| 1090 | cefalexin (after) | cefoxitin (after) | cefadroxil (after) | cefradine (after) |
| 1091 | piperacillin and beta-lactamase inhibitor (after) | cefradine (after) | cefazolin (after) | cefadroxil (after) |
| 1092 | ampicillin and beta-lactamase inhibitor (after) | cefadroxil (after) | cefalexin (after) | cefazolin (after) |
| 1093 | piperacillin (after) | cefalotin (after) | benzathine benzylpenicillin (after) | cefalexin (after) |
| 1094 | sulbactam (after) | dicloxacillin (after) | piperacillin and beta-lactamase inhibitor (after) | benzathine benzylpenicillin (after) |
| 1095 | oxacillin (after) | cefalexin (after) | amoxicillin and beta-lactamase inhibitor (after) | piperacillin and beta-lactamase inhibitor (after) |
| 1096 | dicloxacillin (after) | piperacillin and beta-lactamase inhibitor (after) | ampicillin and beta-lactamase inhibitor (after) | amoxicillin and beta-lactamase inhibitor (after) |
| 1097 | benzathine benzylpenicillin (after) | amoxicillin and beta-lactamase inhibitor (after) | sulbactam (after) | ampicillin and beta-lactamase inhibitor (after) |
| 1098 | phenoxymethylpenicillin (after) | ampicillin and beta-lactamase inhibitor (after) | oxacillin (after) | sulbactam (after) |
| 1099 | benzylpenicillin (after) | sulbactam (after) | dicloxacillin (after) | oxacillin (after) |
| 1100 | azelaic acid (after) | oxacillin (after) | dupilumab (after) | dicloxacillin (after) |
| 1101 | clindamycin (after) | Antibiotics (after) | Wart and anti-corn preparations (after) | dupilumab (after) |
| 1102 | benzoyl peroxide (after) | dupilumab (after) | nitroprusside (after) | tacrolimus (after) |
| 1103 | bisoprolol (after) | tacrolimus (after) | carvedilol (after) | Wart and anti-corn preparations (after) |
| 1104 | fosinopril (after) | felodipine (after) | irbesartan (after) | amlodipine (after) |
| 1105 | ramipril (after) | telmisartan (after) | valsartan (after) | candesartan (after) |
| 1106 | enalapril (after) | candesartan (after) | losartan (after) | irbesartan (after) |
| 1107 | captopril (after) | irbesartan (after) | ACE inhibitors and calcium channel blockers (after) | valsartan (after) |
| 1108 | diltiazem (after) | valsartan (after) | fosinopril (after) | losartan (after) |
| 1109 | verapamil (after) | losartan (after) | ramipril (after) | ACE inhibitors and calcium channel blockers (after) |
| 1110 | lercanidipine (after) | ACE inhibitors and calcium channel blockers (after) | enalapril (after) | fosinopril (after) |
| 1111 | nimodipine (after) | fosinopril (after) | captopril (after) | ramipril (after) |
| 1112 | nifedipine (after) | ramipril (after) | diltiazem (after) | enalapril (after) |
| 1113 | nicardipine (after) | enalapril (after) | verapamil (after) | captopril (after) |
| 1114 | felodipine (after) | captopril (after) | lercanidipine (after) | diltiazem (after) |
| 1115 | amlodipine (after) | diltiazem (after) | nimodipine (after) | verapamil (after) |
| 1116 | carvedilol (after) | verapamil (after) | nifedipine (after) | lercanidipine (after) |
| 1117 | labetalol (after) | lercanidipine (after) | nicardipine (after) | nimodipine (after) |
| 1118 | nebivolol (after) | nimodipine (after) | felodipine (after) | nifedipine (after) |
| 1119 | ACE inhibitors and calcium channel blockers (after) | nifedipine (after) | candesartan (after) | nicardipine (after) |
| 1120 | losartan (after) | olmesartan medoxomil (after) | telmisartan (after) | telmisartan (after) |
| 1121 | valsartan (after) | azilsartan medoxomil (after) | olmesartan medoxomil (after) | olmesartan medoxomil (after) |
| 1122 | candesartan and diuretics (after) | losartan and diuretics (after) | telmisartan and amlodipine (after) | azilsartan medoxomil (after) |
| 1123 | valsartan, amlodipine and hydrochlorothiazide (after) | valsartan, amlodipine and hydrochlorothiazide (after) | simvastatin (after) | candesartan and amlodipine (after) |
| 1124 | candesartan and amlodipine (after) | atorvastatin (after) | aliskiren (after) | fluvastatin (after) |
| 1125 | telmisartan and amlodipine (after) | fluvastatin (after) | valsartan and sacubitril (after) | simvastatin (after) |
| 1126 | olmesartan medoxomil and amlodipine (after) | simvastatin (after) | olmesartan medoxomil, amlodipine and hydrochlorothiazide (after) | aliskiren (after) |
| 1127 | valsartan and amlodipine (after) | aliskiren (after) | valsartan, amlodipine and hydrochlorothiazide (after) | valsartan and sacubitril (after) |
| 1128 | telmisartan and diuretics (after) | valsartan and sacubitril (after) | candesartan and amlodipine (after) | olmesartan medoxomil, amlodipine and hydrochlorothiazide (after) |
| 1129 | irbesartan and diuretics (after) | olmesartan medoxomil, amlodipine and hydrochlorothiazide (after) | olmesartan medoxomil and amlodipine (after) | valsartan, amlodipine and hydrochlorothiazide (after) |
| 1130 | irbesartan (after) | candesartan and amlodipine (after) | azilsartan medoxomil (after) | telmisartan and amlodipine (after) |
| 1131 | valsartan and diuretics (after) | valsartan and diuretics (after) | valsartan and amlodipine (after) | losartan and diuretics (after) |
| 1132 | losartan and diuretics (after) | telmisartan and amlodipine (after) | telmisartan and diuretics (after) | olmesartan medoxomil and amlodipine (after) |
| 1133 | azilsartan medoxomil (after) | olmesartan medoxomil and amlodipine (after) | candesartan and diuretics (after) | valsartan and amlodipine (after) |
| 1134 | olmesartan medoxomil (after) | valsartan and amlodipine (after) | irbesartan and diuretics (after) | telmisartan and diuretics (after) |
| 1135 | telmisartan (after) | telmisartan and diuretics (after) | valsartan and diuretics (after) | candesartan and diuretics (after) |
| 1136 | candesartan (after) | candesartan and diuretics (after) | losartan and diuretics (after) | irbesartan and diuretics (after) |
| 1137 | esmolol (after) | irbesartan and diuretics (after) | amlodipine (after) | valsartan and diuretics (after) |
| 1138 | atenolol (after) | nicardipine (after) | labetalol (after) | felodipine (after) |
| 1139 | valsartan and sacubitril (after) | amlodipine (after) | atorvastatin (after) | carvedilol (after) |
| 1140 | metoprolol (after) | Wart and anti-corn preparations (after) | nebivolol (after) | isotretinoin (after) |
| 1141 | hydrochlorothiazide and potassium-sparing agents (after) | carvedilol (after) | xantinol nicotinate (after) | labetalol (after) |
| 1142 | eplerenone (after) | ergoloid mesylates (after) | nicotinic acid (after) | pentoxifylline (after) |
| 1143 | spironolactone (after) | pentoxifylline (after) | phentolamine (after) | xantinol nicotinate (after) |
| 1144 | bumetanide (after) | xantinol nicotinate (after) | tolvaptan (after) | nicotinic acid (after) |
| 1145 | furosemide (after) | nicotinic acid (after) | hydrochlorothiazide and potassium-sparing agents (after) | phentolamine (after) |
| 1146 | indapamide (after) | phentolamine (after) | eplerenone (after) | tolvaptan (after) |
| 1147 | Thiazides, plain (after) | tolvaptan (after) | spironolactone (after) | hydrochlorothiazide and potassium-sparing agents (after) |
| 1148 | riociguat (after) | hydrochlorothiazide and potassium-sparing agents (after) | bumetanide (after) | eplerenone (after) |
| 1149 | macitentan (after) | eplerenone (after) | furosemide (after) | spironolactone (after) |
| 1150 | ambrisentan (after) | spironolactone (after) | indapamide (after) | bumetanide (after) |
| 1151 | bosentan (after) | bumetanide (after) | Thiazides, plain (after) | furosemide (after) |
| 1152 | nitroprusside (after) | furosemide (after) | riociguat (after) | indapamide (after) |
| 1153 | minoxidil (after) | indapamide (after) | macitentan (after) | Thiazides, plain (after) |
| 1154 | hydralazine (after) | Thiazides, plain (after) | ambrisentan (after) | riociguat (after) |
| 1155 | doxazosin (after) | riociguat (after) | bosentan (after) | macitentan (after) |
| 1156 | tolvaptan (after) | macitentan (after) | pentoxifylline (after) | ambrisentan (after) |
| 1157 | phentolamine (after) | nicergoline (after) | ergoloid mesylates (after) | ergoloid mesylates (after) |
| 1158 | nicotinic acid (after) | phenoxybenzamine (after) | nicergoline (after) | nicergoline (after) |
| 1159 | cinchocaine (after) | hydrocortisone (after) | propranolol (after) | phenoxybenzamine (after) |
| 1160 | nadolol (after) | nadolol (after) | esmolol (after) | sotalol (after) |
| 1161 | sotalol (after) | labetalol (after) | bisoprolol (after) | nebivolol (after) |
| 1162 | propranolol (after) | nebivolol (after) | atenolol (after) | esmolol (after) |
| 1163 | C05CX93 (after) | esmolol (after) | metoprolol (after) | bisoprolol (after) |
| 1164 | heparinoid, combinations (after) | bisoprolol (after) | nadolol (after) | atenolol (after) |
| 1165 | organo-heparinoid (after) | atenolol (after) | sotalol (after) | metoprolol (after) |
| 1166 | Local anesthetics (after) | metoprolol (after) | C05CX93 (after) | nadolol (after) |
| 1167 | xantinol nicotinate (after) | sotalol (after) | phenoxybenzamine (after) | propranolol (after) |
| 1168 | betamethasone (after) | betamethasone (after) | heparinoid, combinations (after) | hydrocortisone (after) |
| 1169 | hydrocortisone (after) | propranolol (after) | organo-heparinoid (after) | C05CX93 (after) |
| 1170 | phenoxybenzamine (after) | C05CX93 (after) | cinchocaine (after) | heparinoid, combinations (after) |
| 1171 | nicergoline (after) | heparinoid, combinations (after) | Local anesthetics (after) | organo-heparinoid (after) |
| 1172 | ergoloid mesylates (after) | organo-heparinoid (after) | betamethasone (after) | cinchocaine (after) |
| 1173 | pentoxifylline (after) | cinchocaine (after) | hydrocortisone (after) | Local anesthetics (after) |
| 1174 | olmesartan medoxomil, amlodipine and hydrochlorothiazide (after) | Local anesthetics (after) | fluvastatin (after) | betamethasone (after) |
| 1175 | aliskiren (after) | rosuvastatin (after) | rosuvastatin (after) | atorvastatin (after) |
| 1176 | adapalene (after) | pitavastatin (after) | isotretinoin (after) | rosuvastatin (after) |
| 1177 | calcipotriol, combinations (after) | gemfibrozil (after) | fusidic acid (after) | pitavastatin (after) |
| 1178 | triamcinolone (after) | fenofibrate (after) | fluocinonide (after) | gemfibrozil (after) |
| 1179 | clobetasone (after) | mometasone (after) | diflucortolone (after) | fluocinonide (after) |
| 1180 | hydrocortisone (after) | fluocinonide (after) | fluocinolone acetonide (after) | diflucortolone (after) |
| 1181 | metronidazole (after) | diflucortolone (after) | betamethasone (after) | fluocinolone acetonide (after) |
| 1182 | podophyllotoxin (after) | fluocinolone acetonide (after) | triamcinolone (after) | betamethasone (after) |
| 1183 | aciclovir (after) | betamethasone (after) | clobetasone (after) | triamcinolone (after) |
| 1184 | tromantadine (after) | triamcinolone (after) | hydrocortisone (after) | clobetasone (after) |
| 1185 | silver sulfadiazine, combinations (after) | clobetasone (after) | metronidazole (after) | hydrocortisone (after) |
| 1186 | silver sulfadiazine (after) | hydrocortisone (after) | podophyllotoxin (after) | metronidazole (after) |
| 1187 | mupirocin (after) | metronidazole (after) | aciclovir (after) | podophyllotoxin (after) |
| 1188 | gentamicin (after) | podophyllotoxin (after) | tromantadine (after) | aciclovir (after) |
| 1189 | neomycin (after) | aciclovir (after) | silver sulfadiazine, combinations (after) | tromantadine (after) |
| 1190 | fusidic acid (after) | tromantadine (after) | silver sulfadiazine (after) | silver sulfadiazine, combinations (after) |
| 1191 | acitretin (after) | silver sulfadiazine, combinations (after) | mupirocin (after) | silver sulfadiazine (after) |
| 1192 | methoxsalen (after) | silver sulfadiazine (after) | gentamicin (after) | mupirocin (after) |
| 1193 | betamethasone (after) | mupirocin (after) | mometasone (after) | gentamicin (after) |
| 1194 | fluocinolone acetonide (after) | fluticasone (after) | fluticasone (after) | mometasone (after) |
| 1195 | diflucortolone (after) | clobetasol (after) | clobetasol (after) | fluticasone (after) |
| 1196 | flumetasone (after) | hydrocortisone and antibiotics (after) | cetrimide (after) | clobetasol (after) |
| 1197 | tretinoin (after) | sulfur (after) | azelaic acid (after) | cetrimide (after) |
| 1198 | sulfur (after) | isotretinoin (after) | clindamycin (after) | azelaic acid (after) |
| 1199 | cetrimide (after) | azelaic acid (after) | benzoyl peroxide (after) | clindamycin (after) |
| 1200 | povidone-iodine (after) | clindamycin (after) | adapalene (after) | benzoyl peroxide (after) |
| 1201 | nitrofural (after) | benzoyl peroxide (after) | tretinoin (after) | adapalene (after) |
| 1202 | mometasone (after) | adapalene (after) | sulfur (after) | tretinoin (after) |
| 1203 | hydrocortisone (after) | tretinoin (after) | povidone-iodine (after) | sulfur (after) |
| 1204 | fluocinonide (after) | cetrimide (after) | hydrocortisone and antibiotics (after) | povidone-iodine (after) |
| 1205 | fluocinolone acetonide and antibiotics (after) | betamethasone and antibiotics (after) | nitrofural (after) | hydrocortisone and antibiotics (after) |
| 1206 | betamethasone and antibiotics (after) | povidone-iodine (after) | mometasone (after) | nitrofural (after) |
| 1207 | hydrocortisone and antibiotics (after) | nitrofural (after) | flumetasone (after) | mometasone (after) |
| 1208 | clobetasol (after) | mometasone (after) | hydrocortisone (after) | flumetasone (after) |
| 1209 | fluticasone (after) | flumetasone (after) | fluocinolone acetonide and antibiotics (after) | hydrocortisone (after) |
| 1210 | mometasone (after) | hydrocortisone (after) | betamethasone and antibiotics (after) | fluocinolone acetonide and antibiotics (after) |
| 1211 | trioxysalen (after) | fluocinolone acetonide and antibiotics (after) | neomycin (after) | betamethasone and antibiotics (after) |
| 1212 | tazarotene (after) | gentamicin (after) | acitretin (after) | neomycin (after) |
| 1213 | simvastatin (after) | neomycin (after) | pitavastatin (after) | fusidic acid (after) |
| 1214 | calcitriol (after) | fusidic acid (after) | methoxsalen (after) | acitretin (after) |
| 1215 | ketoconazole (after) | clotrimazole (after) | ciclopirox (after) | atorvastatin and amlodipine (after) |
| 1216 | clotrimazole (after) | combinations (after) | sertaconazole (after) | ciclopirox (after) |
| 1217 | atorvastatin and amlodipine (after) | ciclopirox (after) | oxiconazole (after) | sertaconazole (after) |
| 1218 | simvastatin and ezetimibe (after) | sertaconazole (after) | sulconazole (after) | oxiconazole (after) |
| 1219 | lovastatin and nicotinic acid (after) | oxiconazole (after) | ketoconazole (after) | sulconazole (after) |
| 1220 | alirocumab (after) | sulconazole (after) | clotrimazole (after) | ketoconazole (after) |
| 1221 | evolocumab (after) | ketoconazole (after) | atorvastatin and amlodipine (after) | clotrimazole (after) |
| 1222 | ezetimibe (after) | atorvastatin and amlodipine (after) | simvastatin and ezetimibe (after) | simvastatin and ezetimibe (after) |
| 1223 | colestyramine (after) | butenafine (after) | lovastatin and nicotinic acid (after) | naftifine (after) |
| 1224 | fenofibrate (after) | simvastatin and ezetimibe (after) | alirocumab (after) | lovastatin and nicotinic acid (after) |
| 1225 | gemfibrozil (after) | lovastatin and nicotinic acid (after) | evolocumab (after) | alirocumab (after) |
| 1226 | pitavastatin (after) | alirocumab (after) | ezetimibe (after) | evolocumab (after) |
| 1227 | rosuvastatin (after) | evolocumab (after) | colestyramine (after) | ezetimibe (after) |
| 1228 | atorvastatin (after) | ezetimibe (after) | fenofibrate (after) | colestyramine (after) |
| 1229 | fluvastatin (after) | colestyramine (after) | gemfibrozil (after) | fenofibrate (after) |
| 1230 | sulconazole (after) | naftifine (after) | combinations (after) | combinations (after) |
| 1231 | oxiconazole (after) | griseofulvin (after) | naftifine (after) | butenafine (after) |
| 1232 | sertaconazole (after) | acitretin (after) | butenafine (after) | methoxsalen (after) |
| 1233 | cadexomer iodine (after) | Tars (after) | D04AX91 (after) | D04AX91 (after) |
| 1234 | calcipotriol (after) | methoxsalen (after) | trioxysalen (after) | trioxysalen (after) |
| 1235 | Tars (after) | trioxysalen (after) | calcipotriol, combinations (after) | calcipotriol, combinations (after) |
| 1236 | D04AX91 (after) | calcipotriol, combinations (after) | tazarotene (after) | tazarotene (after) |
| 1237 | lidocaine (after) | tazarotene (after) | calcitriol (after) | calcitriol (after) |
| 1238 | diphenhydramine (after) | calcitriol (after) | calcipotriol (after) | calcipotriol (after) |
| 1239 | Centella asiatica herba (after) | calcipotriol (after) | Tars (after) | Tars (after) |
| 1240 | carbamide (after) | D04AX91 (after) | lidocaine (after) | lidocaine (after) |
| 1241 | ciclopirox (after) | terbinafine (after) | griseofulvin (after) | griseofulvin (after) |
| 1242 | Zinc products (after) | lidocaine (after) | diphenhydramine (after) | diphenhydramine (after) |
| 1243 | terbinafine (after) | diphenhydramine (after) | Centella asiatica herba (after) | Centella asiatica herba (after) |
| 1244 | griseofulvin (after) | Centella asiatica herba (after) | cadexomer iodine (after) | cadexomer iodine (after) |
| 1245 | butenafine (after) | cadexomer iodine (after) | carbamide (after) | carbamide (after) |
| 1246 | naftifine (after) | carbamide (after) | Zinc products (after) | Zinc products (after) |
| 1247 | combinations (after) | Zinc products (after) | terbinafine (after) | terbinafine (after) |
| 1248 | radium (223 Ra) dichloride (after) | radium (223 Ra) dichloride (after) | radium (223 Ra) dichloride (after) | radium (223 Ra) dichloride (after) |

**Supplementary Figures 1**. Shapley additive explanations method for selecting the top 10 features in extreme gradient boost, 30-day

| 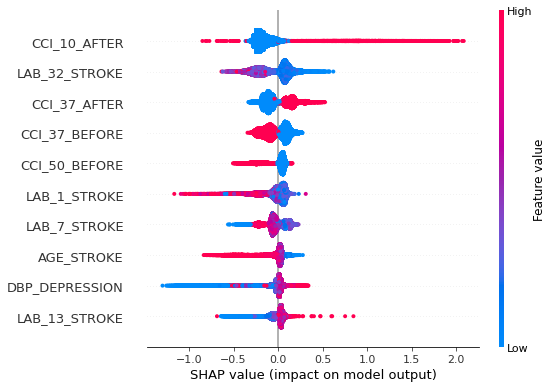 | 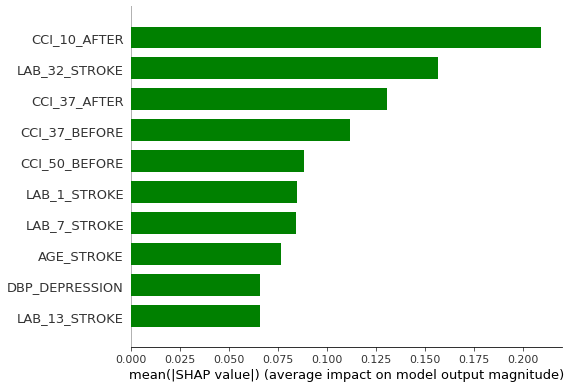 |
| --- | --- |

CCI_10_AFTER = Sleep-wake disorders (after), LAB_32_STROKE = Blood urea nitrogen (during), CCI_37_AFTER = Hypertension (after), CCI_37_BEFORE = Hypertension (before), CCI_50_BEFORE = Bleed (before), LAB_1_STROKE = White blood cell (during), LAB_7_STROKE = Mean corpuscular hemoglobin concentration (during), AGE_STROKE = Age (during), DBP_DEPRESSION = DBP (after), LAB_13_STROKE = White blood cell-lymphocyte (during).

**Supplementary Figures 2.** Shapley additive explanations method for selecting the top 10 features in extreme gradient boost, 90-day

| 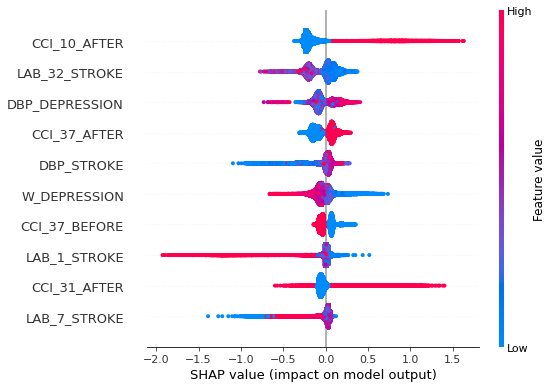 | 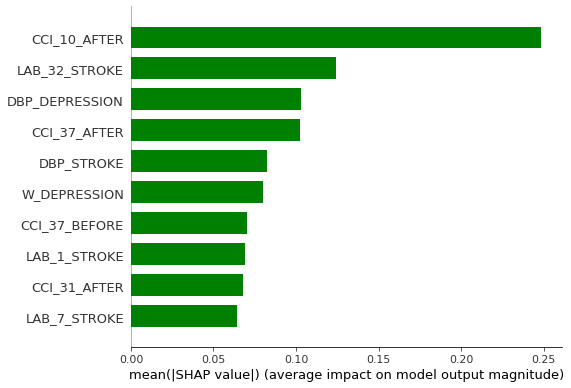 |
| --- | --- |

CCI_10_AFTER = Sleep-wake disorders (after), LAB_32_STROKE = Blood urea nitrogen (during), DBP_DEPRESSION = Diastolic blood pressure (after), CCI_37_AFTER = Hypertension (after), DBP_STROKE = Diastolic blood pressure (during), W_DEPRESSION = Weight (after), CCI_37_BEFORE = Hypertension (before), LAB_1_STROKE = White blood cell (during), CCI_31_AFTER = Hemiplegia (after), LAB_7_STROKE = Mean corpuscular hemoglobin concentration (during).

**Supplementary Figures 3.** Shapley additive explanations method for selecting the top 10 features in extreme gradient boost, 180-day

| 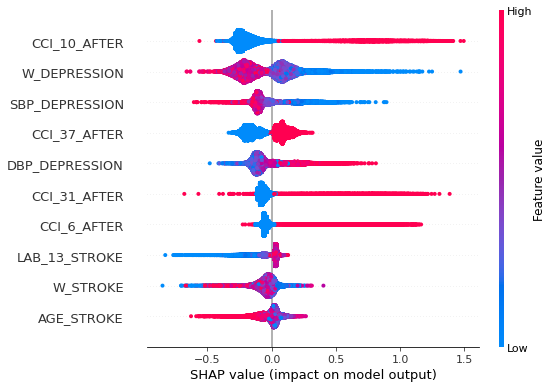 | 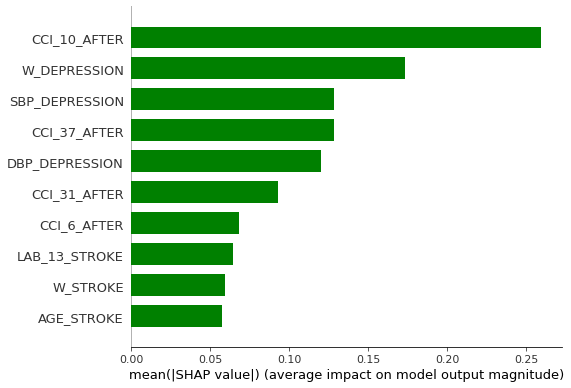 |
| --- | --- |

CCI_10_AFTER = Sleep-wake disorders (after), W_DEPRESSION = Weight (after), SBP_DEPRESSION = Systolic blood pressure (after), CCI_37_AFTER = Hypertension (after), DBP_DEPRESSION = Diastolic blood pressure (after), CCI_31_AFTER = Hemiplegia (after), CCI_6_AFTER = Anxiety disorders (after), LAB_13_STROKE = White blood cell-lymphocyte (during), W_STROKE = Weight (during), AGE_STROKE = Age (during).

**Supplementary Figures 4.** Shapley additive explanations method for selecting the top 10 features in extreme gradient boost, 365-day

| 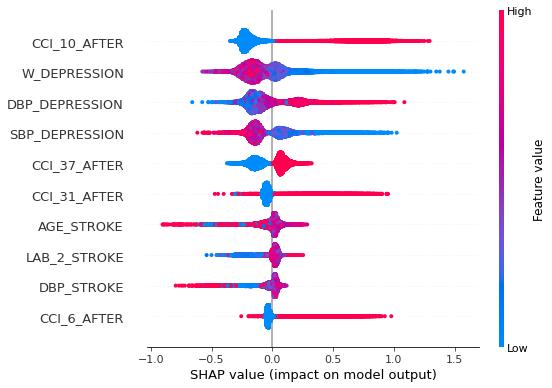 | 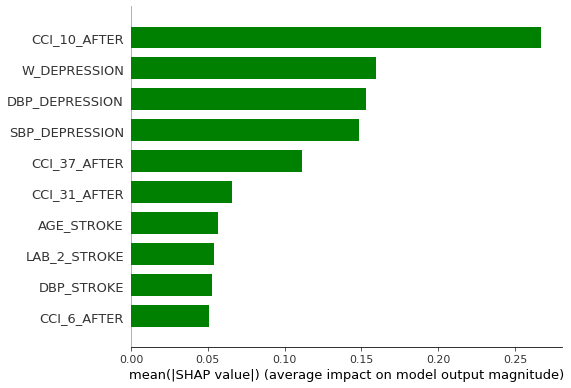 |
| --- | --- |

CCI_10_AFTER = Sleep-wake disorders (after), W_DEPRESSION = Weight (after), DBP_DEPRESSION = Diastolic blood pressure (after), SBP_DEPRESSION = SBP (after), CCI_37_AFTER = Hypertension (after), CCI_31_AFTER = Hemiplegia (after), AGE_STROKE = Age (during), LAB_2_STROKE = Red blood cell (during), DBP_STROKE = DBP (during), CCI_6_AFTER = Anxiety disorders (after).
